# Epstein–Barr virus-induced gene 3 commits human mesenchymal stem cells to differentiate into chondrocytes via endoplasmic reticulum stress sensor

**DOI:** 10.1371/journal.pone.0279584

**Published:** 2022-12-22

**Authors:** Tong Zhang, Kaoru Yamagata, Shigeru Iwata, Koshiro Sonomoto, Gulzhan Trimova, Anh Phuong Nguyen, He Hao, Yu Shan, Mai-Phuong Nguyen, Shingo Nakayamada, Yoshiya Tanaka

**Affiliations:** 1 First Department of Internal Medicine, School of Medicine, University of Occupational and Environmental Health, Kitakyushu, Fukuoka, Japan; 2 Department of Clinical Subjects, High School of Medicine, Faculty of Medicine and Health Care, Al-Farabi Kazakh National University, Almaty, Kazakhstan; 3 Endocrinology & Diabetes Department, Bach Mai Hospital, Hanoi, Vietnam; 4 Department of Internal Medicine, Affiliated Cancer Hospital of Zhengzhou University, Henan Cancer Hospital, Zhengzhou, China; Faculty of Medicine, University of Belgrade, SERBIA

## Abstract

Mesenchymal stem cells (MSC) can differentiate into chondrocytes. Epstein–Barr virus-induced gene 3 (EBI3) is differentially expressed during chondrogenic differentiation and can be produced by MSC. EBI3 is also a subunit of interleukin (IL)-27 and IL-35, and it accumulates in the endoplasmic reticulum (ER) when its partners, such as IL-27 p28 and IL-35 p35, are insufficient. ER stress induced by protein accumulation is responsible for chondrogenic differentiation. However, the role of EBI3 and its relevance to the ER stress in chondrogenic differentiation of MSC have never been addressed. Here, we demonstrate that EBI3 protein is expressed in the early stage of chondrogenic differentiation of MSC. Additionally, knockdown, overexpression, or induction of EBI3 through IL-1β inhibits chondrogenesis. We show that EBI3 localizes and accumulates in the ER of MSC after overexpression or induction by IL-1β and TNF-α, whereas ER stress inhibitor 4-phenylbutyric acid decreases its accumulation in MSC. Moreover, EBI3 modulates ER stress sensor inositol-requiring enzyme 1 α (IRE1α) after induced by IL-1β, and MSC-like cells coexpress EBI3 and IRE1α in rheumatoid arthritis (RA) synovial tissue. Altogether, these data demonstrate that intracellular EBI3 commits to chondrogenic differentiation by regulating ER stress sensor IRE1α.

## Introduction

Mesenchymal stem cells (MSCs) are multipotent stem cells that are easily accessible in multiple tissues such as bone marrow, adipose tissue, and other mesodermal tissues. Under certain conditions, MSCs differentiate into several cell lineages that include chondrocytes, osteoblasts, and osteocytes [1]. We have reported that activation of Interleukin (IL)-6 signaling contributes to chondrogenic differentiation of MSCs *in vitro* and partial repair of damaged articular cartilage in antigen-induced arthritis rats as a model of rheumatoid arthritis (RA) *in vivo* [2, 3]. However, it is generally believed that MSCs localized in cartilage tissue *in vivo* do not differentiate easily into chondrocytes because of the lack of blood vessels and it is difficult for damaged cartilage tissue to completely repair. Therefore, elucidating the mechanism of efficient differentiation of MSCs into chondrocytes may lead to an optimized treatment for articular cartilage.

Many autocrine signaling events play critical roles in the enhancement or maintenance of MSC functions such as cell proliferation, differentiation, migration, and immunoregulation [4–9]. MSCs also have anti-inflammatory and immunosuppressive activities through paracrine factors such as TGF-β1 [10–12]. In terms of immunosuppression, it has been reported that MSCs highly express and secrete Epstein–Barr virus-induced gene 3 (EBI3) which is a subunit of anti-inflammatory and anti-immunosuppressive cytokines IL-27 and IL-35 [13]. Furthermore, *EBI3* is differentially expressed during chondrogenic differentiation in a mouse micromass culture system examined by microarray analyses [14]. However, the role of EBI3 in chondrogenesis remains unclear.

EBI3 heterodimerizes with IL-27 p28 or IL-35 p35 to form immunoregulatory cytokines IL-27 or IL-35, respectively [15]. Intracellular EBI3 undergoes N-linked glycosylation, a post-translational modification, which occurs in the endoplasmic reticulum (ER) [16]. The ER is a reticulated organelle in which proteins undergo proper folding. About 30% of proteins synthesized in normal cells are misfolded and parts of these proteins are refolded to their correct structures. However, other misfolded proteins accumulate in the ER in some cases and result in ER stress followed by cellular dysfunction [17, 18]. Therefore, after non-glycosylated EBI3 proteins translocate to the ER, some of them will localize in a misfolded state. EBI3 also associates with a molecular chaperon on the ER membrane, namely Calnexin, and accumulates in the ER when the levels of its counterparts, such as IL-27 p28 and IL-35 p35, are low [19, 20]. Interestingly, intracellular EBI3 accumulated in murine macrophages inhibits caspase-3-mediated apoptosis after treated with virulent Mycobacterium tuberculosis [21]. Moreover, EBI3 localized in the ER plays a critical role in augmenting the levels of IL-23Rα protein in cooperation with Calnexin in murine T cells [22]. Thus, EBI3 plays crucial roles not only extracellularly, but also intracellularly.

Additionally, *EBI3* is a target gene for nuclear factor kappa B (NF-κB) in human intestinal endothelial cells [23] and proinflammatory cytokines, such as IL-1β, TNF-α, and IL-18, which are upstream of NF-κB, induce expression of *EBI3* in human macrophage cell line KG1 [24]. Rheumatoid arthritis (RA) is a systemic autoimmune and inflammatory disease characterized by synovitis that leads to progressive cartilage defects. Proinflammatory cytokines such as IL-1β and TNF-α induce matrix metalloproteinases (MMPs) that destroy the cartilage matrix, followed by cartilage disorder in RA [25]. In addition, EBI3 is shown to be upregulated in RA synovium under inflammatory conditions [26]. Based on the evidence mentioned above, EBI3 might localize and accumulate in the ER especially under inflammatory conditions.

For chondrocytes to secrete large amounts of cartilage matrix, it is necessary to efficiently transport secreted molecules from the ER to the Golgi apparatus or cell membrane. Therefore, it is believed that the ER is more active and functional in chondrocytes. Interestingly, ER stress triggered by the accumulation of unfolded or misfolded proteins plays a crucial role in the chondrogenic differentiation of MSCs [27, 28]. ER stress induces the unfolded protein response (UPR) through the activation of ER stress sensors that include activating transcription factor 6 (ATF6), inositol-requiring enzyme 1 (IRE1), and protein kinase RNA-like endoplasmic reticulum kinase (PERK) [29–31]. Whereas overexpression of ATF6 enhances chondrogenesis within 3 days, silencing ATF6 suppresses chondrogenesis [32]. Low levels of splicing variant X-box-binding protein 1 (XBP1s), downstream of IRE1, suppresses chondrogenic differentiation of MSCs [33]. Proper activation of PERK is indispensable to maintain chondrocyte homeostasis [34]. However, downregulation of ATF4, a transcriptional factor induced after activation of PERK, suppressed the expression of chondrogenic markers, such as *SOX9* and *COL2A1*, in murine ATDC5 chondrogenic cells [35]. These studies indicate that ER stress sensors play critical roles in transmitting their signals to nuclei and cytoplasm, which leads to chondrogenesis of MSCs. A series of chemical chaperones that function in the ER, which include 4-phenylbutyric acid (4-PBA) and tauroursodeoxycholic acid (TUDCA), enhance proper protein folding and alleviate ER stress [36–43]. IL-35 is highly secreted into the blood of *apolipoprotein E*-deficient mice treated with 4-PBA [44]. This result indicates that 4-PBA decreases the levels of misfolded EBI3 in the ER and leads to high secretion of EBI3 as a counterpart of IL-35 p35 followed by the generation of IL-35.

In this study, we focused on EBI3, which plays a functional role in the ER, and elucidated its role in chondrogenic differentiation of MSCs related to ER stress.

## Materials and methods

### Cell culture

Human bone marrow-derived MSCs were purchased from Lonza and used at passages 2–4. Cell surface markers CD29, CD44, CD105, and CD166 were positive, while CD14, CD34, and CD45 were negative as shown by the manufacturer. The cells were cultured at 37°C with 5% CO_2_ in MSC growth medium (C-28009, Takara, Japan) to 80% confluence to prevent spontaneous differentiation.

### Antibodies and reagents

Antibodies were used for immunohistochemistry (IHC), immunofluorescence (IF), immunocytochemistry (ICC), immunoprecipitation (IP) and immunoblotting (IB). Information on the antibodies and reagents is provided in S1 Table.

### Chondrogenic culture

Chondrogenesis of MSCs was induced as described previously [3]. Briefly, after human MSCs at 80% confluence were trypsinized, 2–3×10^5^ cells/well were seeded in a low-adhesion U-bottom 96-well plate (Corning) that were centrifuged at 400×*g* for 5 minutes. The cells were cultured in a commercial chondrogenic medium (C-28012, Takara, Japan) that was replaced every 3 days. The cells were stimulated with cytokines (S1 Table), which included recombinant human IL-1β, IL-6, soluble IL-6 Receptor (sIL-6R), TNF-α, and IL-17A, at 10 ng/ml during chondrogenesis. Cell pellets were collected at the indicated time points for analyses.

### Western blotting

Western blotting was carried out as described previously [45]. Briefly, MSC pellets or monolayered cells at the indicated time points were sonicated (on/off cycle time: 30 sec/30 sec; cycle number: 5) in TNE lysis buffer, which consisted of 50 mM Tris (pH 8.0), 150 mM NaCl, 1% Nonidet P40, and a protease inhibitor cocktail (A-0014-20, ITSI Biosciences, Johnstown, PA), and centrifuged at 12,000×*g* for 30 min at 4°C. The supernatant was used as a whole cell lysate. Proteins (10 μg) were separated in a 4%–20% and 10% SDS-PAGE Tris-glycine gel and then transferred onto a 0.2 μm nitrocellulose membrane (GE Healthcare, Chicago, IL). Immunoblotting was performed with primary antibodies followed by the appropriate secondary antibody. β-Actin was used as loading control. To quantify band intensities, densitometric analyses were performed using ImageJ software. The relative value of each band was calculated as the intensity of the target band divided by that of the loading control.

### Detection of extracellular EBI3

Detection of EBI3 in a culture supernatant was performed as described previously [46]. Briefly, supernatants of cell pellets were harvested and concentrated using a Microcon (MRCPRT010, Millipore, Germany) in accordance with the manufacturer’s instructions. EBI3 levels in culture supernatants were determined by WB as described above. Proteins in the gel were stained with Coomassie brilliant blue R-350 (GE Healthcare, Chicago, IL) in accordance with the manufacturer’s instructions.

### Histological examination

Immunostaining was performed as described previously [3]. Briefly, cell pellets were harvested at 21 days of chondrogenic culture, fixed overnight in 10% buffered formalin, and prepared for embedding in paraffin. To detect matrix proteoglycans, 4-μm-thick sections were stained with a 0.1% Safranin O (S-O) solution (Muto Pure Chemicals, Japan) for 30 seconds and then counterstained with hematoxylin. Immunohistochemistry was carried out as described previously [2] with slight modifications. The sections were deparaffinized, hydrated, and incubated for 15 minutes in 0.4 mg/ml proteinase K (Dako, Santa Clara, CA). The sections were then incubated in blocking solution (Dako) for 30 min, followed by incubation with an anti-type II collagen antibody in Can get Signal immunostain solution A (NKB-501, Toyobo, Japan) overnight at 4°C. The sections were rinsed with PBS and then incubated with Simple stain rat MAX-PO (R) for 30 min. Antigens were visualized using a 3,3ʹ-diaminobenzidine tetrahydrochloride substrate (Dako) and then counterstained with hematoxylin.

Patients with RA (n = 3) and patients with osteoarthritis (OA; n = 3), who met the diagnostic criteria, consented to provide synovium samples. This study was conducted with approval of the ethics committee of the University of Occupational and Environmental Health. For IF microscopy of synovium tissues, sections were deparaffinized, hydrated, incubated with proteinase K, blocked, and then incubated for 2 hours with anti-CD271, anti-CD105, anti-EBI3, or anti-IRE1α antibodies in Can Get Signal immunostain solution A, followed by incubation for 1 hour with anti-goat, anti-mouse, or anti-rabbit secondary antibodies, respectively. The sections were rinsed with PBS, coverslipped, and examined under a BIOREVO BZ-X800 fluorescence microscope (Nikon).

### Ethics declarations

The Ethics Committee of Medical Research, University of Occupational and Environmental Health, Japan, reviewed and approved this study (approval number: H28-067). A signed informed consent was obtained from all subjects in accordance with the Declaration of Helsinki and its subsequent modifications.

### Immunocytochemistry (ICC)

ICC of MSCs was performed as described previously [46] with slight modifications. The cells were fixed in 1% paraformaldehyde (Wako, Japan) for 20 minutes, washed with PBS, and then permeabilized in PBS with 0.5% (v/v) Triton X-100 for 5 minutes. They were blocked in PBS with 10% (v/v) fetal bovine serum for 30 minutes and then incubated overnight at 4°C with anti-Calnexin and anti-EBI3 antibodies in Can Get Signal immunostain solution A. Sections were then washed with PBS and incubated for 1 hour with Rhodamine-labeled anti-mouse and FITC-labeled anti-rabbit secondary antibodies, and then incubated with DAPI for 30 minutes. They were then rinsed with PBS, coverslipped, and examined under the BIOREVO BZ-X800 fluorescence microscope.

### Reverse transcription (RT)-quantitative PCR

RT-qPCR was performed as described previously [2] with slight modifications. Chondrogenic cell pellets and monolayer-cultured MSCs were lysed in RLT buffer (Qiagen). Total RNA was purified using an RNeasy Mini kit (Qiagen) and first-strand cDNA was synthesized using a high-capacity cDNA reverse transcription kit in accordance with the manufacturers’ instructions. Real-time PCR was performed in a StepOne Plus system (Applied Biosystems). The primer/probe pairs used in TaqMan Gene Expression Assays (Applied Biosystems) are shown in S2 Table. The relative quantities of transcript were analyzed using the 2^-ΔΔCt^ method and then normalized to that of *GAPDH*.

### Transfection of MSCs with siRNA

*EBI3* siRNAs (s19760 and s19762) were purchased from Invitrogen. Transfection was performed as described previously [47] with slight modifications. Briefly, MSCs were seeded at 1×10^4^ cells/cm^2^ on a 12-well plate or culture flask (Easy Flask 75; Nunc) in MSC growth medium. The cells were treated with transfection reagents that contained 20 or 120 pmol siRNAs and 6 or 20 μl Lipofectamine^®^ RNAiMAX (13778075, Invitrogen) diluted in 100 or 500 μl Opti-MEM (31985062, Invitrogen), respectively, and then incubated at 37°C for 48 hours prior to assays.

### Transfection of MSCs with plasmid DNA

The transfection was performed as described previously [47] with slight modifications. pEF6-V5-*hEBI3* (MJS_002) was purchased from Addgene (#72490). Empty pEF6-V5 (V96120, Thermo Fisher Scientific, Waltham, MA) was used for the mock control. MSCs were seeded at 1×10^4^ cells/cm^2^ on a 12-well plastic plate or culture flask in MSC growth medium. Transfection reagents that contained 4 or 16 μl Lipofectamine^®^ 3000, 4 μl or 16 μl P3000™ (L3000-008, Invitrogen, Waltham, MA) and 2 or 8 μg pEF6-V5-*hEBI3* or pEF6-V5 in a final volume of 100 or 500 μl Opti-MEM were added to each well or flask with seeded MSCs.

### Immunoprecipitation

For checking the association between EBI3 and p62, protein was collected at day2 of chondrogenesis. Incubate EBI3 antibody with samples overnight at 4°C, after that add 50 μl protein A agarose into it and incubate for 2 hours. After wash and elute the complex, WB were performed using antibodies described above. For checking the association between EBI3 and IRE1α, MSCs were transfected with pEF6-EBI3-V5 plasmid DNA and protein was collected. Add anti-V5-tag pAb-Agarose into 100 μg protein in 100 μl of lysis buffer containing protease inhibitors and incubate for 2 hours at 4°C. After washing and elution, WB were performed as mentioned before.

### Statistical analysis

All quantitative data are expressed as the mean±SD. Differences between two groups were tested for statistical significance by the Student’s unpaired two-tailed *t*-test. To compare more than three groups, analysis of variance (ANOVA) was used. If the ANOVA result was significant, Dunnett’s multiple comparison test was used as a post hoc test. Statistical analyses were performed using GraphPad Prism version 8.00 software. *P*-values less than 0.05 were considered significant.

## Results

### MSCs produce EBI3 in cells during chondrogenic differentiation

**Fig 1:**
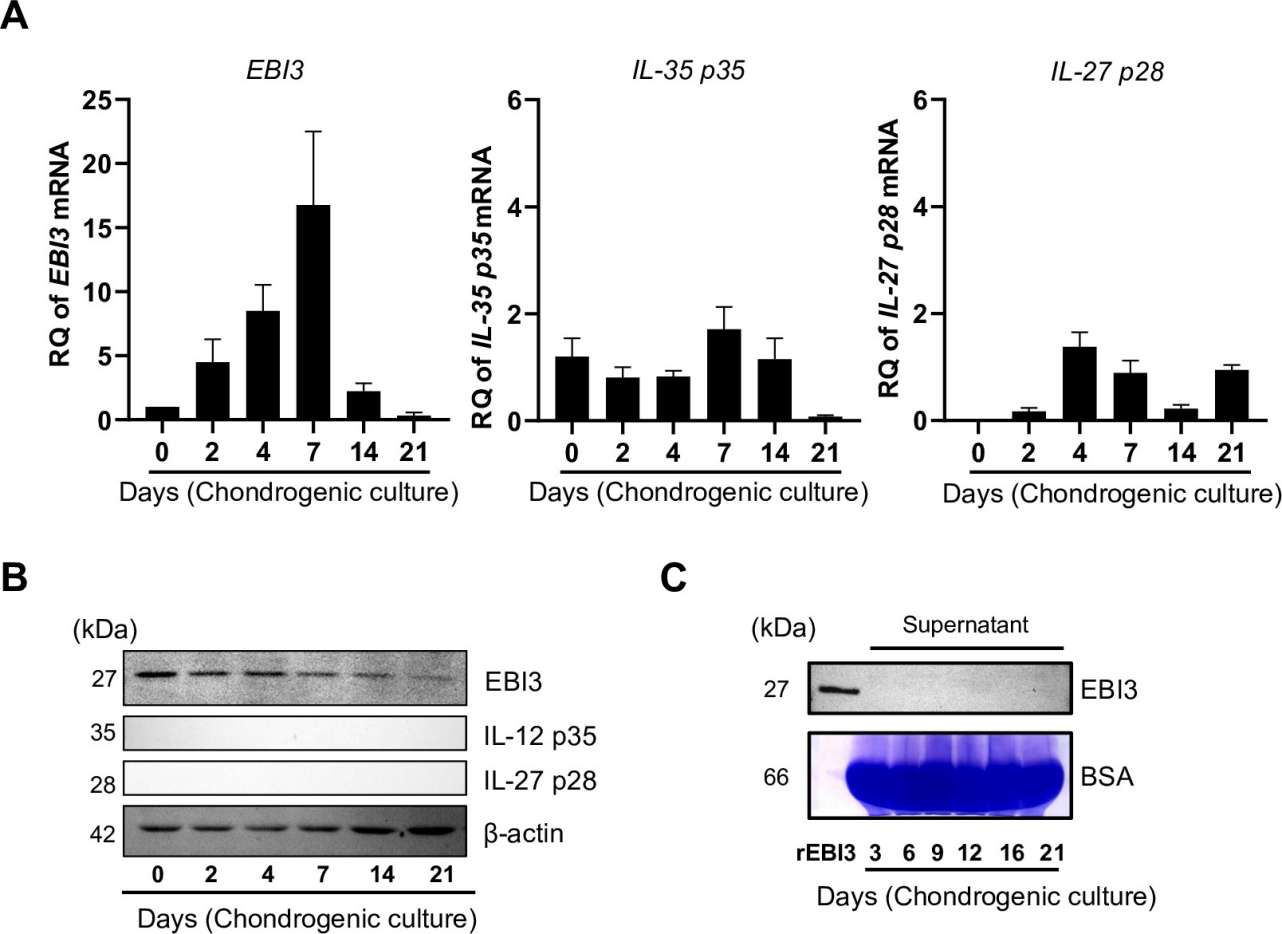
MSCs produce EBI3 in cells during chondrogenic differentiation. **(A-C)** Human MSCs were cultured as aggregates in chondrogenic medium. **(A)** *EBI3*, *IL-35 p35* and *IL-27 p28* mRNA levels were evaluated by RT-qPCR at the indicated time points. All quantitative data are expressed as the mean ± SD (each n = 3). **(B)** EBI3, IL-35 p35, and IL-27 p28 protein levels in chondrogenic MSCs were detected at the indicated time points by Western blotting. β-actin was used as a loading control. Results are representative of 3 independent experiments with similar findings. **(C)** EBI3 protein levels in culture supernatants during chondrogenesis were detected by Western blotting. BSA was detected by CBB staining and used as loading control. Results are representative of 3 independent experiments with similar findings.

Reportedly, MSCs produce EBI3 constitutively. Therefore, we initially examined the expression of EBI3 during chondrogenesis. mRNA expression of *EBI3* increased gradually with a peak on day 7 during the early stage of chondrogenesis, although its expression level had decreased from day 7 (Fig 1A). Non-glycosylated EBI3 protein was also produced during chondrogenesis (Fig 1B), while *IL-27 p28* and *IL-35 p35* mRNAs were detected at low levels, although none of the proteins were detected (Fig 1A and 1B). Moreover, no EBI3 was detected in the supernatants of chondrogenic cultures (Fig 1C). Since commercial EBI3 antibodies which can specifically detect glycosylated EBI3 are not produced, we used recombinant EBI3 as non-glycosylated positive control. Extracellular EBI3 mainly plays functional roles as the subunit of IL-27 and IL-35. In contrast, functional role of intracellular EBI3 has not been well studied. Since we found EBI3 was accumulated in the early of chondrogenesis of MSCs, intracellular EBI3 might have a unique function that differs from that of extracellular EBI3. Notably, the tendency was inconsistent in EBI3 expression levels between mRNA and protein (Fig 1A and 1B) during chondrogenesis. As shown in Fig 1C, there was less EBI3 in the culture supernatant, indicating that EBI3 was not secreted. p62 is an autophagosome cargo protein that targets other proteins that bind to it for selective autophagy. Therefore, we checked the expression level of p62 during chondrogenesis. We found p62 decreased gradually in early chondrogenesis (S7A Fig). To make sure of the association between EBI3 and p62, we performed Coomassie brilliant blue (CBB) and immunoprecipitation (IP). These assays showed EBI3 physically associated with p62 (S7B Fig).

### Endogenous EBI3 enhances chondrogenic differentiation of MSCs

**Fig 2:**
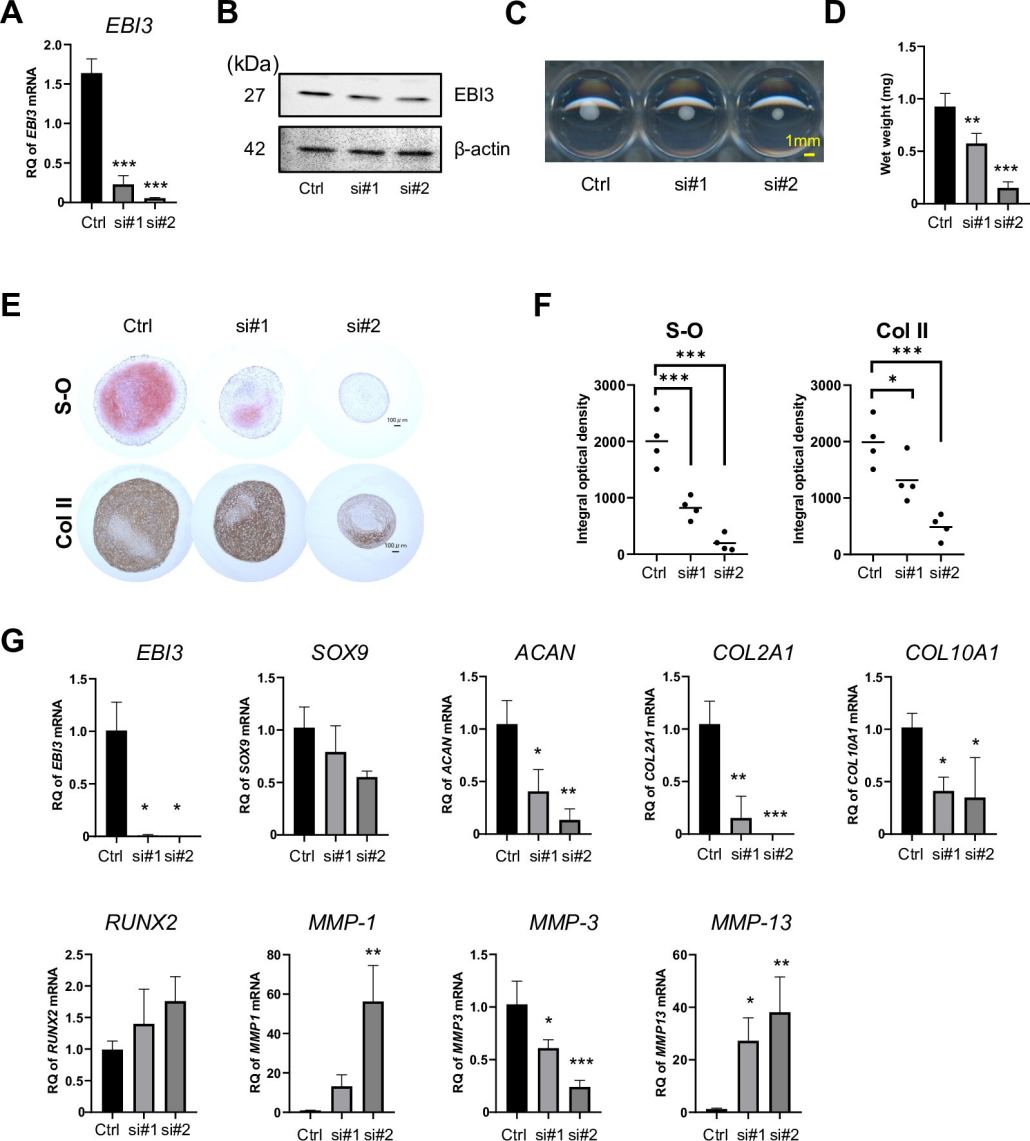
Endogenous EBI3 enhances chondrogenic differentiation of MSCs. **(A-G)** MSCs were transfected with *control* siRNA or 2 different *EBI3* siRNAs (si#1 and si#2). **(A)** *EBI3* mRNA levels were evaluated by RT-qPCR at 48 hrs after transfection. **(B)** EBI3 protein levels were evaluated by western blotting at 48 hrs after transfection. β-actin was used as a loading control. **(C-G)** MSCs were cultured as pellets in chondrogenic medium for 21 days after transfection. **(C)** Photographs of the pellets. Scale bar, 1 mm. **(D)** The wet weight of the pellets. **(E)** The pellets cultured were stained with Safranin-O (S-O) or anti-type II collagen (Col II) antibody. Scale bar, 100 μm. **(C and E)** Results are representative of 4 independent experiments with similar findings. **(F)** Densitometric analysis of the staining in **(E)** was performed and shown by integral optical density (IOD). **(G)** The mRNA levels of the indicated genes in transfected pellets were determined by RT-qPCR. **(A, D, F, and G)** Quantified data are expressed as the mean ± SD (each n = 3 in **A;** n = 4 in D; n = 4 in **F**; n = 3 in **G**) with similar findings. * = P < 0.05; ** = P <0.01; *** = P <0.001 by Dunnett’s multiple comparison test.

To examine role of EBI3 in chondrogenic differentiation, we suppressed expression of EBI3 by transfection of two siRNAs (#1 and #2) with different sequences into MSCs. Knockdown efficacy was examined by RT-qPCR (Fig 2A) and Western blotting (Fig 2B) at 48 hours post-transfection. The results indicated that both *EBI3* siRNAs significantly suppressed expression of the EBI3 protein and *EBI3* gene in MSCs.

After transfection for 2 days, MSCs were subjected to pellet culture in chondrogenic medium for 21 days. The size and wet weight of cell pellets transfected with *EBI3*#1 and #2 siRNAs were significantly smaller and lighter, respectively, than those of cell pellets transfected with *control* siRNA (Fig 2C and 2D). Furthermore, the volumes of the cartilage matrix in MSCs transfected with *EBI3#1* and *#2* siRNAs were decreased as detected by Safranin O (S-O) staining and immunostaining with an anti-type II collagen (Col Ⅱ) antibody (Fig 2E and 2F). We next evaluated the expression of chondrogenic marker genes, such as *SOX9*, *ACAN*, *COL2A1*, and *COL10A1*, and chondrocyte-suppressive genes such as *RUNX2*, *MMP-1*, *MMP-3*, and *MMP-13* (Fig 2G). The results revealed that knocking down *EBI3* downregulated chondrogenic marker gene expression such as *ACAN*, *COL2A1*, and *COL10A1*. Moreover, knocking down *EBI3* significantly decreased *MMP-3* expression and increased *MMP-13* expression. *EBI3#2* siRNAs significantly increased the expression of *MMP-1*. The effect of knockdown of *EBI3* by siRNA sustained during chondrogenesis (Fig 2G and S11A Fig).

### EBI3 overexpression in MSCs inhibits chondrogenic differentiation

**Fig 3:**
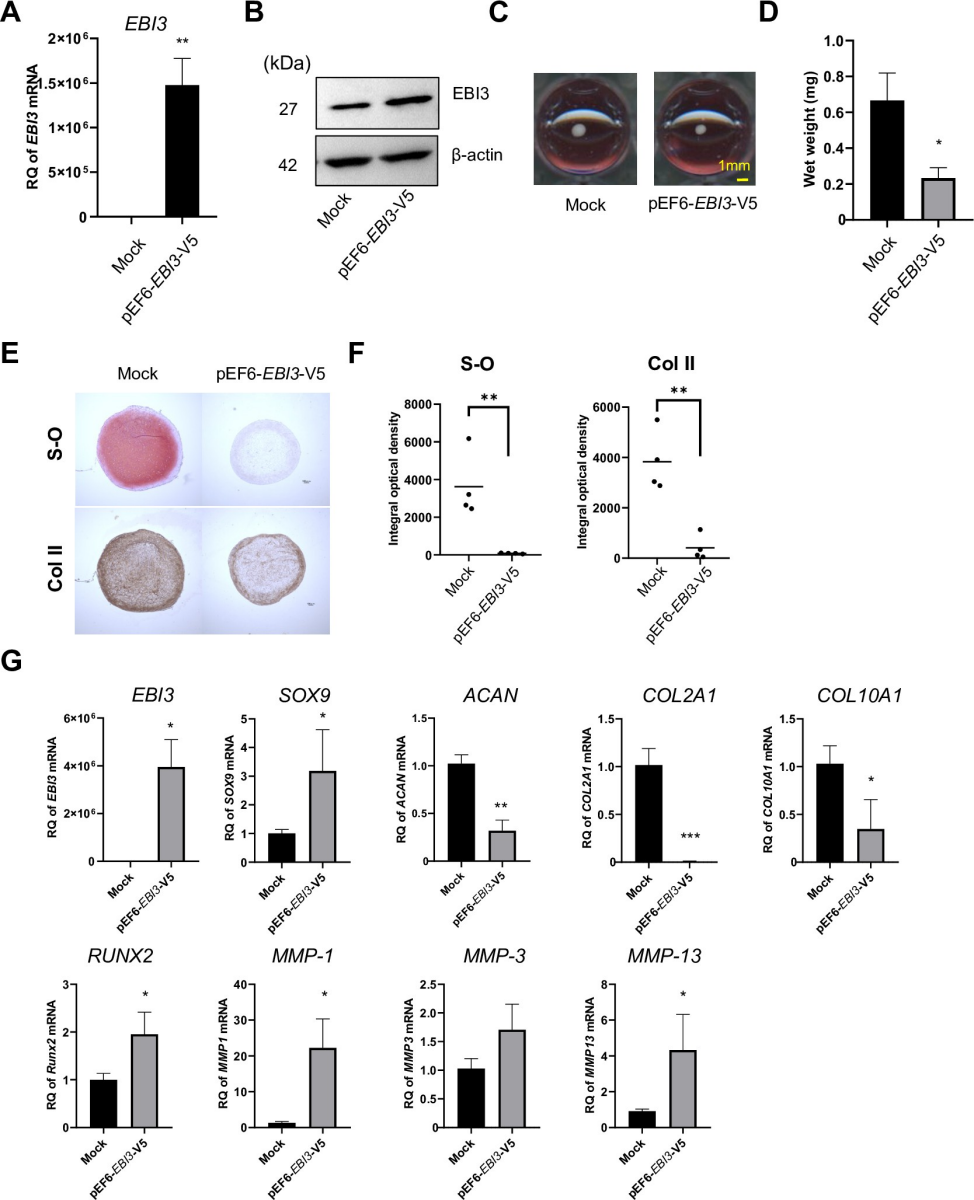
EBI3 overexpression in MSCs inhibits chondrogenic differentiation. (A-G) MSCs were transiently transfected with pEF6-*EBI3*-V5 plasmid DNA. (A) *EBI3* mRNA levels were evaluated by RT-qPCR at 24 hrs after transfection. (B) EBI3 protein levels were evaluated by western blotting at 72 hrs after transfection. β-actin was used as a loading control. (C-G) MSCs were cultured as pellets in chondrogenic medium for 21 days after transfection with pEF6-*EBI3*-V5 or empty vector. (C) Photographs of the pellets. Scale bar, 1mm. (D) Wet weight of the pellets. (E) Aggregates were stained with Safranin O (S-O) or anti-type II collagen (Col II) antibody. Scale bar, 100 μm. Results are representative of 4 independent experiments with similar findings. (F) Densitometric analysis of the stained of (E) was performed and shown by integral optical density (IOD). (G) The mRNA levels of the indicated genes between transfected aggregates were determined by RT-qPCR on day 21. (A, D, F, and G) Quantified data are expressed as the mean ± SD with similar findings (each n = 3 in A, D, and G; n = 4 in F). * = P < 0.05; ** = P <0.01; *** = P <0.001 by Student’s unpaired 2-tailed t-test.

Because knocking down EBI3 inhibited chondrogenesis, we hypothesized that overexpression of EBI3 may promote chondrogenesis. We transfected MSCs with pEF6-*EBI3*-V5 plasmid DNA or pEF6-V5 (mock) and measured mRNA and protein expression after 24 and 72 hours (Fig 3A and 3B). After transfection with pEF6-*EBI3*-V5 or pEF6-V5, MSCs were subjected to pellet culture in chondrogenic medium for 21 days. The size and wet weight of pellets in pEF6-*EBI3*-V5 group were significantly decreased compared to those in the mock control (Fig 3C and 3D). The cartilage matrix was reduced as shown by Safranin O staining and immunostaining with an anti-type II collagen antibody in the pEF6-*EBI3*-V5 group compared to the mock control (Fig 3E and 3F). Expression of *ACAN*, *COL2A1*, and *COL10A1* genes, but not *SOX9*, was significantly decreased in the pEF-*EBI3*-V5 group compared to the mock control. *RUNX2*, *MMP-1*, and *MMP-13* expression was significantly upregulated in pEF6-*EBI3*-V5 group compared to the mock control (Fig 3G). The effect of overexpression of *EBI3* by plasmid DNA persisted during chondrogenesis (Fig 3G and S11B Fig).

### IL-1β up-regulates EBI3 and inhibits chondrogenic differentiation of MSCs

**Fig 4:**
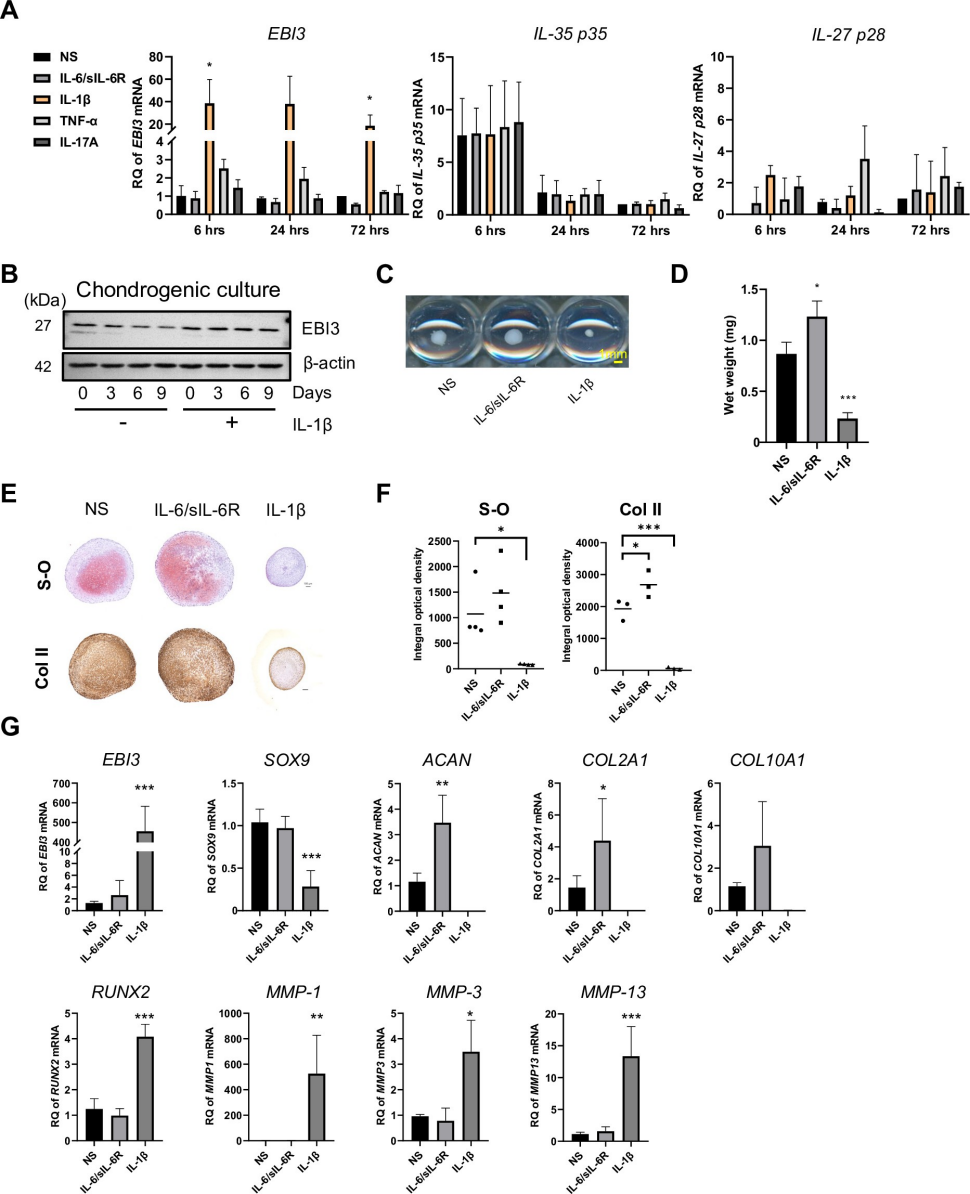
IL-1β up-regulates EBI3 and inhibits chondrogenic differentiation of MSCs. **(A-G)** MSCs were cultured as cell pellets in chondrogenic medium. **(A)** The mRNA levels of the indicated genes were determined by RT-qPCR at the indicated time points. **(B)** EBI3 protein levels in chondrogenic MSCs treated with or without IL-1β were determined at the indicated time points by Western blotting. β-actin was used as a loading control. Results are representative of 3 independent experiments with similar findings. **(C-G)** MSCs were cultured with no stimulation (NS), IL-6/sIL-6R or IL-1β for 21 days. **(C)** Photographs of the pellets. Scale bar, 1mm. **(D)** Wet weight of the pellets. **(E)** Pellets were stained with Safranin O (S-O) or anti-type II collagen (Col II) antibody. Scale bar, 100 μm. Results are representative of 4 independent experiments with similar findings. **(F)** Densitometric analysis of **(E)** was performed to show integral optical density (IOD). **(G)** The mRNA levels of the indicated genes in cell pellets were determined by RT-qPCR on day 21. **(A, D, F, G)** Quantified data are expressed as the mean ± SD (each n = 2 in **A**; n = 3 in **D**; n = 4 in **F**; n = 4 in **G**). * = P<0.05; ** = P<0.01; *** = P<0.001 by Dunnett’s multiple comparison test.

To investigate the role of endogenous EBI3 in chondrogenesis, we first applied proinflammatory cytokines to stimulate MSCs in a monolayer. We found IL-1β and TNF-α, but not IL-6/sIL-6R or IL-17A, enhanced expression of the *EBI3* gene (S1A Fig). IL-1β and TNF-α induced mRNA expression of EBI3 in a dose-dependent manner at up to 3 and 100 ng/ml, respectively (S1B Fig). Next, to investigate the involvement of endogenous EBI3 induced by proinflammatory cytokines during chondrogenic differentiation, we stimulated MSCs with a series of cytokines. IL-1β, but not IL-6/sIL-6R, TNF-α, or IL-17A, induced expression of the *EBI3* gene (Fig 4A). Induction of expression of IL-35 p35 or IL-27 p28 by those proinflammatory cytokines was not found. In contrast to the gradual decrease in EBI3 protein expression during chondrogenic differentiation, IL-1β stimulation maintained intracellular EBI3 expression (Fig 4B). MSCs treated with IL-6/sIL-6R or IL-1β were subjected to pellet culture in chondrogenic medium for 21 days. The size and wet weight of pellets were significantly reduced in the IL-1β group, but not in the IL-6/sIL-6R group (Fig 4C and 4D). Accumulation of cartilage matrix was decreased in MSCs treated with IL-1β, but not IL-6/sIL-6R compared to the unstimulated group (Fig 4E and 4F). Additionally, expression of the *EBI3* gene at day 21 significantly increased in MSCs treated with IL-1β, but not IL-6/sIL-6R. Moreover, IL-1β downregulated chondrogenic genes, which included *SOX9*, *ACAN*, *COL2A1*, and *COL10A1*, and upregulated chondrogenic repressor genes that included *RNUX2*, *MMP-1*, *MMP-3*, and *MMP-13*. IL-6/sIL-6R mainly induced expression of *ACAN and COL2A1* genes (Fig 4G).

### EBI3 proteins localize and accumulate in the endoplasmic reticulum of MSCs and modulate ER stress sensor IRE1α after induced by IL-1β

**Fig 5:**
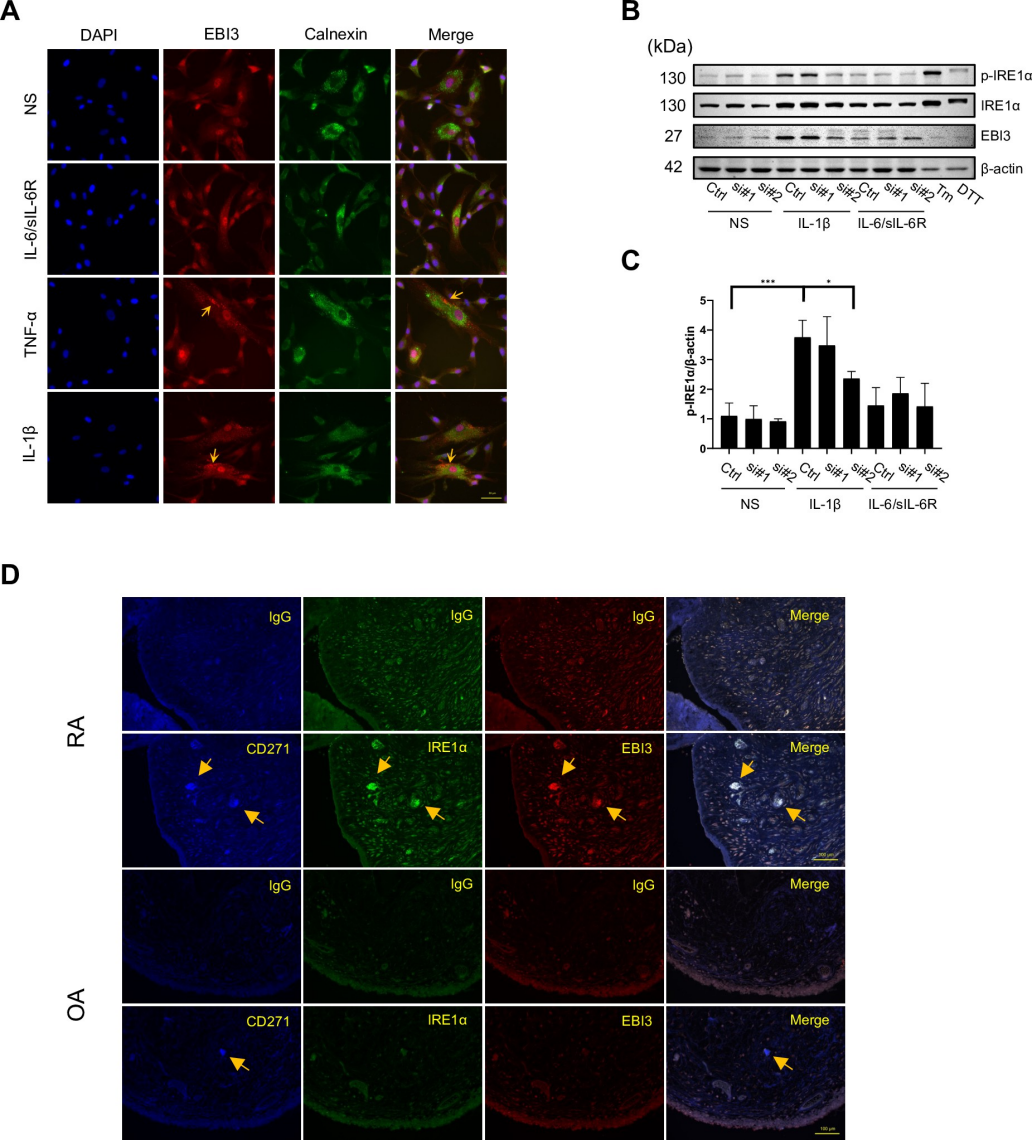
EBI3 proteins localize in the ER of MSCs and modulate IRE1α under inflammatory conditions. **(A-C)** MSCs were cultured in a monolayer in growth medium. **(A)** Human MSCs were stimulated with IL-6/sIL-6R (100 ng/ml), TNF-α (100 ng/ml) or IL-1β (3 ng/ml). The immunocytochemistry detected EBI3 and Calnexin. Nucleus was stained by DAPI (5 μg/ml). Images are representative of 3 independent experiments with similar findings. Scale bar, 50 μm. **(B)** Human MSCs were transfected with *control* siRNA or 2 different *EBI3* siRNAs (si#1 and si#2) for 48 hrs. After that growth medium was refreshed, IL-1β and IL-6/sIL-6R were then added for 36 hrs. MSCs treated with Tunicamycin (Tm) and DTT were used as positive control of ER stress. Whole-cell lysates were analyzed for each ER stress sensors and EBI3 by Western blotting. **(C)** Densitometric analysis of the Western blotting in **B** was performed, and the data were normalized to β-actin. Quantified data are expressed as the mean± SD (each n = 3). * = P < 0.05; *** = P <0.001 by Dunnett’s multiple comparison test. **(D)** Synovial specimens from RA patients (n = 3) and OA patients (n = 3) were stained by immunofluorescence using specific antibodies against CD271, EBI3, and IRE1α. Results are representative of 3 independent experiments with similar findings. Scale bar, 100 μm.

EBI3 needs to be post-translationally modified in the ER, which produces a glycosylated protein. To examine the localization of EBI3 in MSCs, immunofluorescence staining was performed after MSCs were stimulated with IL-6/sIL-6R, IL-1β, and TNF-α in a monolayer. Calnexin was used as an ER marker. The results showed EBI3 was induced by IL-1β and TNF-α, and it partially localized in the ER (Fig 5A). We also transfected MSCs with the pEF6-*EBI3*-V5 plasmid and found that EBI3 protein localized in the ER (S2 Fig). We further analyzed ER stress sensor IRE1α to evaluate the relationship between EBI3 and ER stress *in vitro*. After MSCs were treated with IL-1β, p-IRE1α was upregulated, while IL-6/sIL-6R was not. p-IRE1α was significantly downregulated in MSCs which underwent knockdown of *EBI3* by *EBI3* siRNA#2, but not #1 (Fig 5B and 5C). Dithiothreitol (DTT) and tunicamycin (Tm) were used as positive controls to identify ER stress sensors (S3 Fig). We also examined ER stress sensors p-IRE1α, p-PERK, and ATF6p50 during chondrogenesis (S5 Fig). We found that EBI3 and p-IRE1α were coexpressed within day 4. We then treated MSCs with ER stress inhibitors, such as 4-PBA and TUDCA, to confirm the localization of EBI3. Treatment of MSCs with 4-PBA significantly decreased the protein levels of intracellular EBI3 (S4A and S4B Fig). In contrast, 4-PBA induced expression of the *EBI3* gene in MSCs (S4C Fig) and meanwhile decreased the secretion of EBI3 in the supernatant (S4D Fig). We also checked the upstream of EBI3 gene after cells were treated with 4-PBA, and found 4-PBA could activate phosphorylation of NF-κB (S9A and S9B Fig), suggesting that 4-PBA may induce the gene expression of EBI3 by activation of NF-κB. The decrease of p62 suggests that the reduction of EBI3 in MSCs and supernatant may be caused by the augment of protein degradation. In addition, we treated MSCs with ER stress inducers, such as DTT and Tm. Tm, but not DTT significantly enhanced the accumulation of EBI3 as detected by Western blot (S8 Fig). To examine the expression of EBI3 and its relevance to ER stress in MSCs under inflammatory conditions *in vivo*, we firstly stained synovial tissue from RA and osteoarthritis (OA) patients with antibodies to detect CD271 (a promising marker for MSC), CD105 (a marker used for MSC isolation), and EBI3 (S6 Fig). CD271^+^CD105^+^ MSC-like cells expressed EBI3 in RA synovial tissue. Conversely, the abovementioned cells were not found in OA synovial tissue. Then we stained synovial tissue with antibodies to detect CD271, IRE1α, and EBI3 (Fig 5D). CD271, IRE1α, and EBI3 were coexpressed in RA, but not in OA, synovial tissue. Collectively, EBI3 was expressed in CD271^+^CD105^+^ MSC-like cells of RA synovium tissue and colocalized with IRE1α. These results indicated that EBI3 was upregulated and might induce ER stress in RA synovium tissue.

## Discussion

Various cytokines contribute to the differentiation of MSCs. Recently, another group found human bone marrow-derived MSCs highly produce EBI3 even without any stimulations. EBI3 is also a subunit of anti-inflammatory and immunosuppressive cytokines such as IL-27 and IL-35, which suggest MSCs may play a suppressive role in the onset and progression of immune disorders by secreting EBI3. Meanwhile, mouse EBI3 was reported differentially expressed during chondrogenesis by microarray analyses. Taken together, it is necessary to elucidate functional roles of human EBI3 in the regulation of inflammation, immunoregulation, and chondrogenesis of MSCs.

EBI3 heterodimerizes with its counterparts IL-27p28 and IL-35p35 to form IL-27 and IL-35, respectively. If these counterparts are insufficient, EBI3 cannot form a dimer with them, and thus EBI3 tends to accumulate in an immature form in the ER associated with the molecular chaperone calnexin and EBI3-associated protein (also named p62) [19, 20]. Interestingly, the inconsistent tendency in EBI3 expression level between mRNA and protein was found in Fig 1A and 1B. Protein secretion or degradation might be responsible for this phenomenon. As shown in Fig 1C, there is less EBI3 in the culture supernatant, meaning EBI3 was not secreted. p62, also named EBI3-associated protein (EBIAP), is an autophagosome cargo protein that targets other proteins that bind to it for selective autophagy. As shown in S7A Fig, p62 gradually decreased in early chondrogenesis which means protein degradation was switched on and gradually enhanced accompanied by chondrogenesis. More importantly, degradation of EBI3 may indeed need cargo p62. Further study is needed to clarify the role of the association between EBI3 and p62. As shown in Figs 1C and S2, EBI3 was not detected in the culture supernatant of MSCs undergoing chondrogenesis and overexpressed EBI3 was detected in the ER, which suggested that EBI3 protein had accumulated in MSCs. Intracellular EBI3, which localizes in the ER, plays functional roles in CD4^+^ T cells under inflammatory conditions. It cooperates with calnexin to enhance IL-23Rα protein expression [22]. Importantly, as a novel function of EBI3 localized in MSCs, we found that EBI3 regulated the differentiation of MSCs into chondrocytes.

First, knocking down EBI3 was optimal to elucidate the physiological functions of endogenous EBI3. As shown in Fig 2C–2G, suppression of EBI3 expression inhibited chondrocyte differentiation, which suggested that endogenous EBI3 promoted the differentiation of MSCs into chondrocytes. Knocking down EBI3 downregulated *MMP-3* and upregulated *MMP-1 and -13* that encode cartilage MMPs, which inhibited chondrocyte differentiation (Fig 2G). Second, the EBI3-overexpressing system is a good model to examine the functions of pathologically expressed EBI3. Overexpression of EBI3 suppressed chondrogenic differentiation of MSCs (Fig 3C–3G), and significantly increased expression of *MMP-1* and *-13*, but not *MMP-3* (Fig 3G). In this model, the expression level of *SOX9* increased. Considering that SOX9 has a suppressive effect on chondrogenic differentiation in the late stage of chondrogenesis [48, 49], high levels of SOX9 induced by EBI3-overexpression might suppress chondrogenesis. Further studies are needed to determine whether the genetic interaction between EBI3 and SOX9 exists. Third, stimulation with pro-inflammatory cytokines is an appropriate approach to induce endogenous EBI3 and investigate the environment that mimics RA pathology. As shown in Fig 4C–4G, stimulation with proinflammatory cytokine IL-1β, but not IL-6/sIL-6R, suppressed chondrocyte differentiation. While *SOX9* was downregulated, *EBI3* and *MMP-1*, *-3*, and *-13* were upregulated. Considering that overexpression of EBI3 inhibited chondrogenesis of MSCs (Fig 3C–3G), high expression of EBI3 in MSCs treated with IL-1β may also directly contribute to inhibition of chondrocyte differentiation (Fig 4B and 4G). It is well known that IL-1β inhibits chondrogenesis by repressing SOX9 expression [50], while overexpression of EBI3 upregulated *SOX9* gene expression (Fig 3G). Whether high levels of EBI3 induced by IL-1β during chondrogenesis weaken the inhibition effect of IL-1β requires further studies.

The direct and functional target of EBI3 in regulating chondrocyte differentiation as mentioned above was unclear. We focused on localization of EBI3 in the ER and examined the relationship between EBI3 and ER stress. The unfolded protein response (UPR) is activated to avoid ER stress. The ER stress sensor (also named UPR sensor) IRE1α induces inflammasome activation and IL-1β production [51–53]. As shown in Fig 5B, both p-IRE1α and total IRE1α were increased by IL-1β, but not IL-6/sIL-6R. Therefore, Il-1β activated IRE1α, and meanwhile, IRE1α induces the IL-1β production which suggests a feedback loop between IL-1β and IRE1α. IL-1β induces ER stress markers, which include binding-immunoglobulin protein (BiP), C/EBP homologous protein (CHOP), growth arrest and DNA damage-inducible protein (GADD34), ATF4, XBP1s, and phospho-eukaryotic Initiation Factor 2 (p-eIF2), in the human pancreatic cancer cell line MIA PaCa-2 [54], which indicates that IL-1β is an important trigger to activate a wide range of ER stress markers. Furthermore, as shown in Fig 5B and 5C, decreased expression of EBI3 suppressed IL-1β-induced phosphorylation of IRE1α. This result suggests that EBI3 is required for IL-1β to activate IRE1α. Further investigation of the relationship between EBI3 and other ER stress markers, such as BiP, CHOP, and XBP1s is needed. Considering that the 3D pellet culture system for chondrogenic differentiation might be unsuitable for immunofluorescence and unstable in protein expression profiles. To solve this problem, we performed a series of experiments with MSCs treated with growth medium and cultured in 2D, then we studied cellular localization of EBI3 and ER stress sensors in MSCs.

ER stress inhibitor, 4-PBA, reduced EBI3 through upregulating protein degradation. ER stress inducer, Tm, but not DTT significantly enhanced the accumulation of EBI3. Tm, a naturally occurring antibiotic, induces ER stress in cells by inhibiting the first step in the biosynthesis of N-linked glycans in the proteins resulting in many misfolded proteins. EBI3 is an N-linked glycoprotein. So, it’s reasonable that Tm can induce the accumulation of EBI3 by inhibiting its glycosylation. While DTT is a powerful reducing agent that induces acute ER stress by disrupting the redox conditions required to form disulfide bridges in proteins which means DTT mostly affects the association between EBI3 and other proteins to form disulfide bonds connected dimers such as IL-27p28/EBI3 and IL-35p35/EBI3. Therefore, DTT may markedly increase EBI3 when EBI3 is induced by IL-1β.

It has been reported that fibroblast-like synoviocytes (FLSs) in RA patients are partially derived from bone marrow-derived MSCs [55]. Additionally, both EBI3 and IRE1α are expressed at high levels in RA FLSs [26, 56]. Considering that proinflammatory conditions such as IL-1β and TNF-α, which are responsible for RA pathology, induced the accumulation of EBI3 in ER (Fig 5A), and IL-1β activated IRE1α by upregulating EBI3 (Fig 5B). Thus, it is reasonable that both EBI3 and IRE1α are highly expressed in MSC-like cells localized in the synovium of RA patients (Fig 5C). Reportedly, IRE1α is involved in the induction and pathogenesis of autoimmune diseases [57], thus EBI3 which is upstream of IRE1α may also participate in the pathophysiology of autoimmune diseases. We performed immunoprecipitation (IP) after MSCs were transfected with V5-tagged EBI3 plasmid DNA. As shown in S10 Fig, IRE1α did not associate with ectopic EBI3, indicating that EBI3 doesn’t regulate IRE1α by directly binding to it. In addition, one group reported that EBI3 plays a pivotal role in the activity of chaperone Calnexin in ER [22]. Indeed, another chaperone named binding-immunoglobulin protein (BiP) modulates the activation of IRE1α through binding to unfolded or misfolded protein. Therefore, further study to elucidate the relationship between EBI3 and chaperones, such as Calnexin and BiP is necessary. In conclusion, elevated EBI3 may be a potential biomarker for MSC under inflammatory conditions such as RA and it may also be a therapeutic target for IRE1α related autoimmune disease.

A limitation of our study is that the sample size of synovial tissue obtained from RA and OA patients was small. Further evaluation using cartilage tissue from RA and OA patients as well as healthy donors will be necessary to delineate the role of intracellular EBI3 expressed by MSC-like cells in cartilage homeostasis.

## Conclusion

EBI3 maintains ER stress under physiological conditions and promotes chondrocyte differentiation. However, high expression of EBI3 may excessively activate IRE1α, thereby inhibiting chondrocyte differentiation. The latter may be partially responsible for the suppression of chondrogenesis in RA patients.

## Supporting information

S1 Fig IL-1β or TNF-α induces gene expression of *EBI3* in MSCs in a dose-dependent manner. **(A-B)** MSCs were stimulated with a series of pro-inflammatory cytokines when cultured in growth medium. **(A)** The mRNA levels of *EBI3*, *IL-27 p28*, and *IL-35 p35* were measured by RT-qPCR at the indicated time point. **(B)** *EBI3* mRNA levels were measured by RT-qPCR after stimulation of MSCs with IL-1β and TNF-α at indicated concentrations. Quantified data are expressed as mean ± SD (n = 3 in A; n = 2 in B). * = P < 0.05; ** = P<0.01; *** = P<0.001 (Dunnett’s multiple comparison test). N.D. = Not determined.
(TIF)

S2 Fig Transiently expressed EBI3 localizes and accumulates in the endoplasmic reticulum of MSCs. MSCs were cultured in monolayer, then transfected with pEF6-*EBI3*-V5 or empty vector for 72 hrs. Immunocytochemistry was performed to stain EBI3 and calnexin. Nuclei were stained with DAPI. Results are representative of 3 independent experiments with similar findings. Scale bar, 20 μm.
(TIF)

S3 Fig MSCs treated with DTT and Tunicamycin express ER stress sensors. MSCs were cultured in monolayer in growth medium. After treatment of MSCs with DTT (5 mM) and Tunicamycin (5 μg/ml), whole-cell lysates were collected at each time point and underwent Western blotting to detect ATF6p50, p-IRE1α, IRE1α, p-PERK, PERK, β-actin. Results are representative of 3 independent experiments with similar findings.
(TIF)

S4 Fig ER stress inhibitor, 4-PBA, reduces the accumulation of EBI3 in MSCs. **(A-D)** MSCs were cultured in a monolayer in growth medium and pretreated with 4-PBA or TUDCA for 6 hrs. After that IL-1β were added, and cells were cultured for 36 hrs. **(A)** Whole-cell lysates were analyzed for EBI3 by Western blotting. **(B)** Densitometric analysis of **A** was performed, and the data were normalized to β-actin. **(C)** Total RNA was collected after the indicated treatment. *EBI3* mRNA levels were measured by RT-qPCR. Quantified data are expressed as the mean ± SD (each n = 3). * = P < 0.05; ** = P < 0.01 by Student’s unpaired 2-tailed t-test. **(D)** Supernatants were analyzed for EBI3 by CBB and Western blotting. Results are representative of 3 independent experiments with similar findings.
(TIF)

S5 Fig ER stress sensors and EBI3 are expressed during chondrogenic differentiation of MSCs. MSCs were cultured in chondrogenic medium in pellet form. At each time, whole-cell lysates were prepared at the indicated times and underwent Western blotting to detect EBI3, p-IRE1α, IRE1α, p-PERK, PERK, ATF6p50, and β-actin. β-actin was used as a loading control. Results are representative of 3 independent experiments with similar findings.
(TIF)

S6 Fig CD271^+^CD105^+^ MSC-like cells expressed EBI3 in RA synovial tissue. Synovial specimens from RA patients (n = 3) and OA patients (n = 3) were stained by immunofluorescence using specific antibodies against CD271, CD105, and EBI3. Results are representative of 3 independent experiments with similar findings. Scale bar, 100 μm.
(TIF)

S7 Fig p62 associated with EBI3 in early stage of chondrogenesis. MSCs were pellet-culured in chondrogenic medium and protein was collected at indicated time points. **(A)** Western blotting was performed to detect p62 and β-actin. **(B)** Protein was immunoprecipitated by EBI3 antibody and protein A agarose. Western blotting and CBB were performed to detect p62. Results are representative of 2 independent experiments with similar findings.
(TIF)

S8 Fig Tunicamycin but not DTT induced the accumulation of EBI3 in human MSCs. MSCs were cultured in monolayer in growth medium. After treatment of MSCs with DTT (5 mM) and Tunicamycin (5 μg/ml) for 9 hours, whole-cell lysates were collected and underwent Western blotting to detect EBI3 and β-actin. Results are representative of 4 independent experiments with similar findings. **(B)** Densitometric analysis of (**A)** was performed, and the data were normalized to β-actin. Quantified data are expressed as the mean ± SD (n = 4). * = P<0.05 by Student’s unpaired 2-tailed t-test.
(TIF)

S9 Fig NF-κB was activated by 4-PBA and might contribute to the upregulation of EBI3. MSCs were cultured in a monolayer in growth medium and pretreated with 4-PBA for 6 hrs. After that IL-1β were added, and cells were cultured for 36 hrs. **(A)** Whole-cell lysates were analyzed for phosphorylation of NF-κB, NF-κB, EBI3, and p62 by Western blotting. **(B)** Densitometric analysis of **A** was performed, and the data were normalized to NF-κB and β-actin. Quantified data are expressed as the mean ± SD (n = 2). * = P<0.05 by Dunnett’s multiple comparison test.
(TIF)

S10 Fig IRE1α was not associated with ectopic EBI3. MSCs were cultured in a monolayer in growth medium and transfected with pEF6-EBI3-V5 plasmid DNA for 48 hrs. After that IL-1β were added, and cells were cultured for 24 hrs. Whole-cell lysates were immunoprecipitated by anti-V5-tag-pAb-agarose and were analyzed for IRE1α and EBI3 by Western blotting. Results are representative of 3 independent experiments with similar findings.
(TIF)

S11 Fig Knocking down and overexpression of EBI3 by siRNA and plasmid DNA have a constant effect during chondrogenesis of human MSCs. (A) MSCs were transfected with control siRNA or 2 different EBI3 siRNAs (si#1 and si#2) and pellet- cultured. (B) MSCs were transfected with empty or pEF6-EBI3-V5 plasmid DNA and pellet-cultured. The EBI3 mRNA levels in transfected pellets were determined by RT-qPCR at the indicated time point. Quantified data are expressed as the mean ± SD (each n = 2~3).
(TIF)

S12 Fig Schematic cartoon showing the potential function of EBI3 during chondrogenesis in human MSCs under different conditions. In normal condition, EBI3 exists in the early stage and maintain the ER stress. While low ER stress caused by EBI3 knockdown or excessive ER stress induced by overexpression of EBI3 result in the inhibition of chondrogenesis. In RA condition, high expression of EBI3 induced by inflammatory cytokines contributes to excessive ER stress and suppression of chondrogenesis.
(TIF)

S1 Table Key resources table. (DOCX)

S2 Table TaqMan gene expression assay (applied biosystems) primer/probe pairs for quantitative reverse transcription (RT-q) PCR. (DOCX)

S1 File Raw data of Fig 1. (ZIP)

S2 File Raw data of Fig 2. (ZIP)

S3 File Raw data of Fig 3. (ZIP)

S4 File Raw data of Fig 4. (ZIP)

S5 File Raw data of Fig 5. (ZIP)

S6 File Raw data of all supplementary figures. (ZIP)
