# Supplementary figures and images for: Epstein–Barr virus-induced gene 3 commits human mesenchymal stem cells to differentiate into chondrocytes via endoplasmic reticulum stress sensor
Source: PLoS One. 2022 Dec 22;17(12):e0279584. doi: 10.1371/journal.pone.0279584 (PMC9778607; doi:10.1371/journal.pone.0279584)

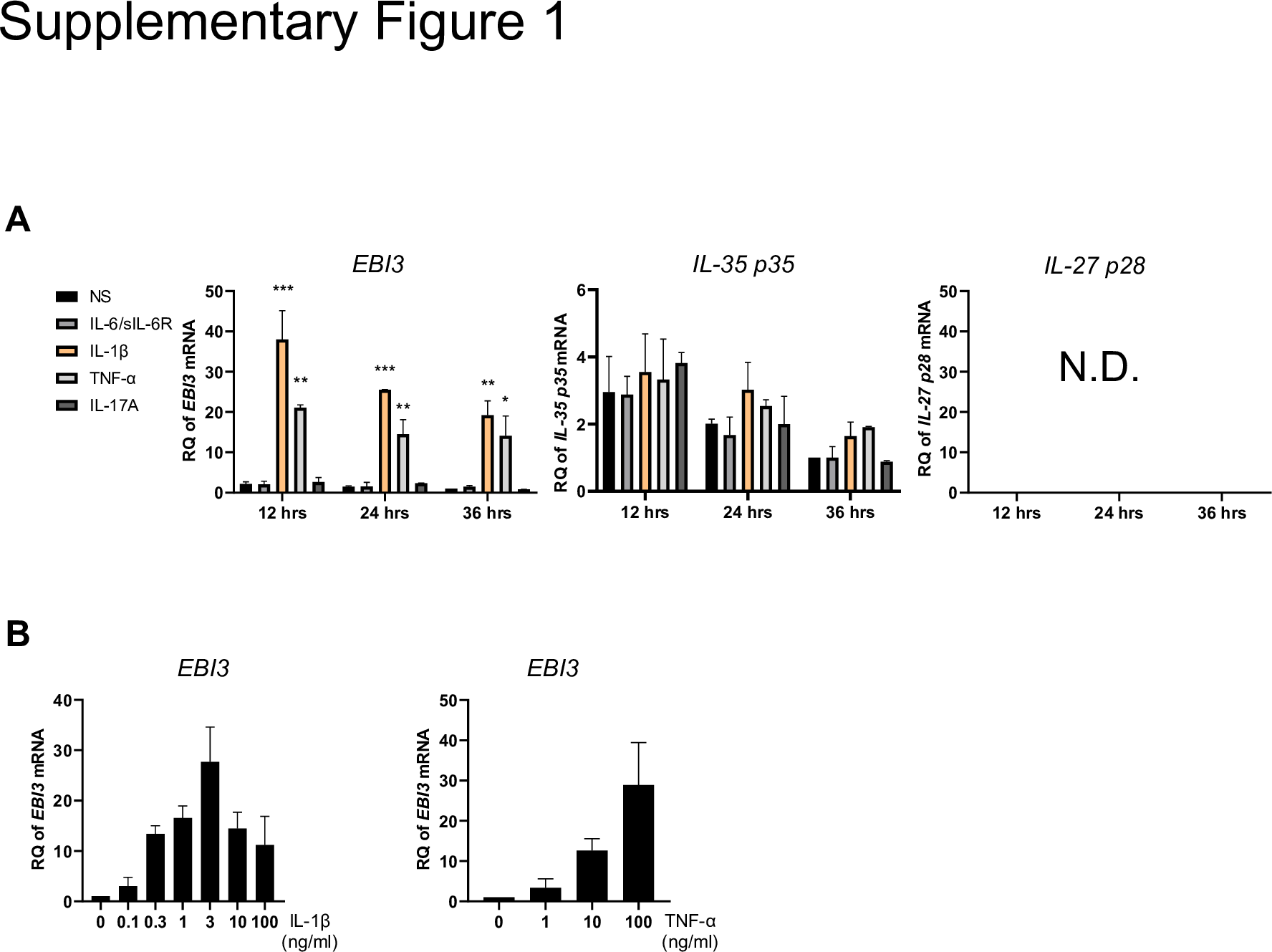

Supplement: S1 Fig — (A-B) MSCs were stimulated with a series of pro-inflammatory cytokines when cultured in growth medium. (A) The mRNA levels of EBI3, IL-27 p28, and IL-35 p35 were measured by RT-qPCR at the indicated time point. (B) EBI3 mRNA levels were measured by RT-qPCR after stimulation of MSCs with IL-1β and TNF-α at indicated concentrations. Quantified data are expressed as mean ± SD (n = 3 in A; n = 2 in B). * = P < 0.05; ** = P<0.01; *** = P<0.001 (Dunnett’s multiple comparison test). N.D. = Not determined. (TIF) [file pone.0279584.s001.tif]

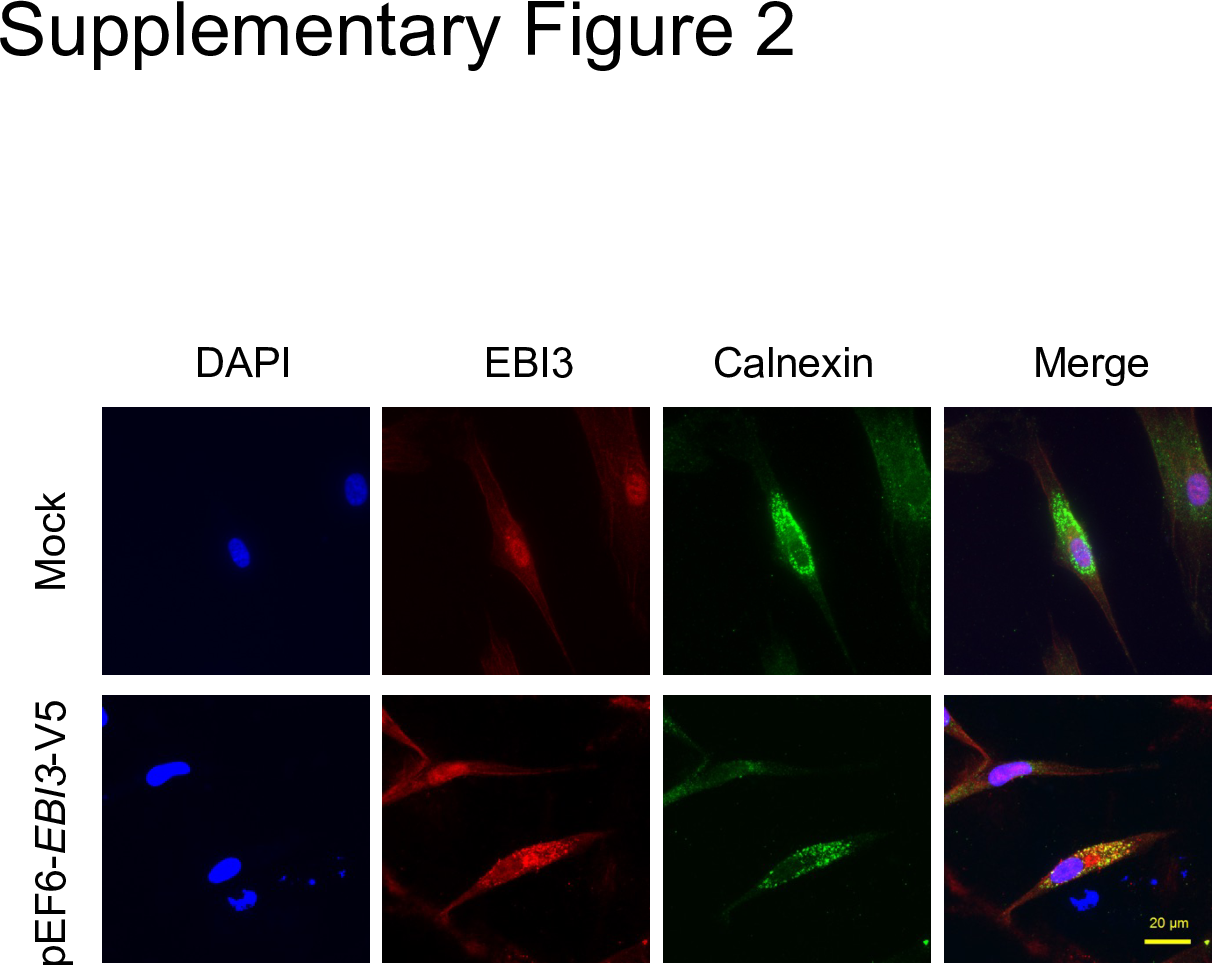

Supplement: S2 Fig — MSCs were cultured in monolayer, then transfected with pEF6-EBI3-V5 or empty vector for 72 hrs. Immunocytochemistry was performed to stain EBI3 and calnexin. Nuclei were stained with DAPI. Results are representative of 3 independent experiments with similar findings. Scale bar, 20 μm. (TIF) [file pone.0279584.s002.tif]

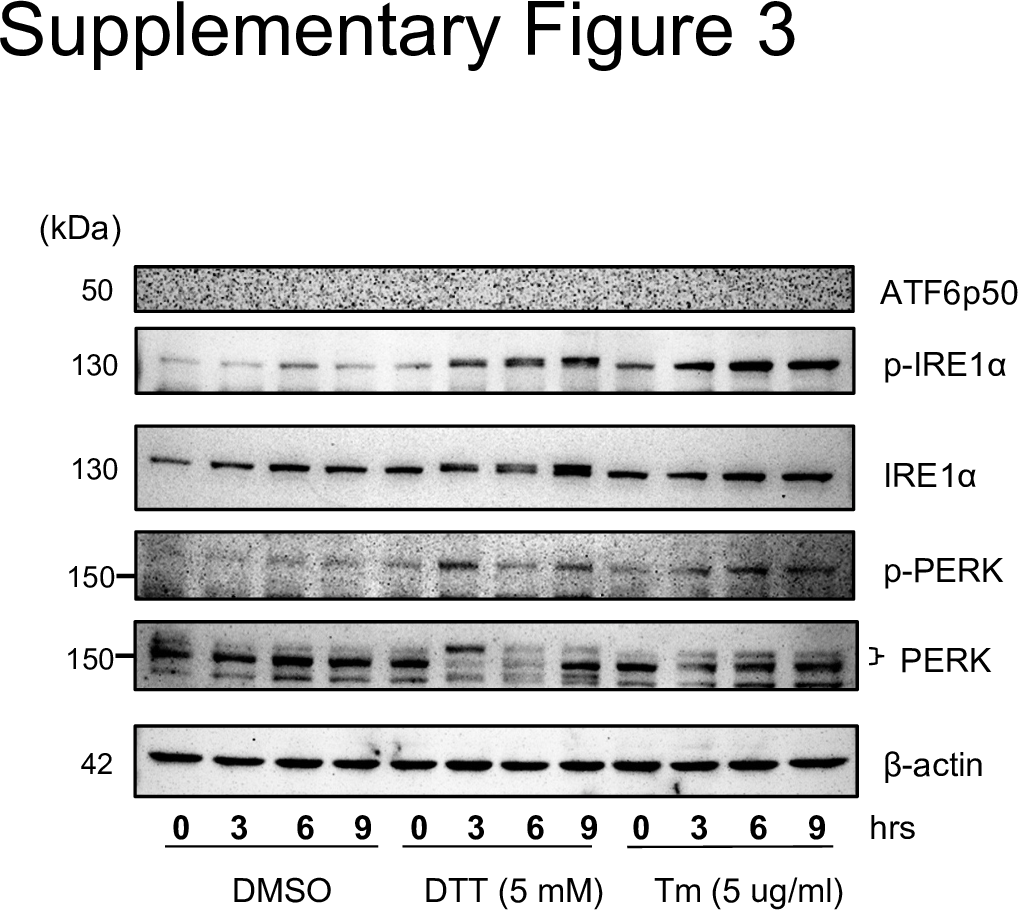

Supplement: S3 Fig — MSCs were cultured in monolayer in growth medium. After treatment of MSCs with DTT (5 mM) and Tunicamycin (5 μg/ml), whole-cell lysates were collected at each time point and underwent Western blotting to detect ATF6p50, p-IRE1α, IRE1α, p-PERK, PERK, β-actin. Results are representative of 3 independent experiments with similar findings. (TIF) [file pone.0279584.s003.tif]

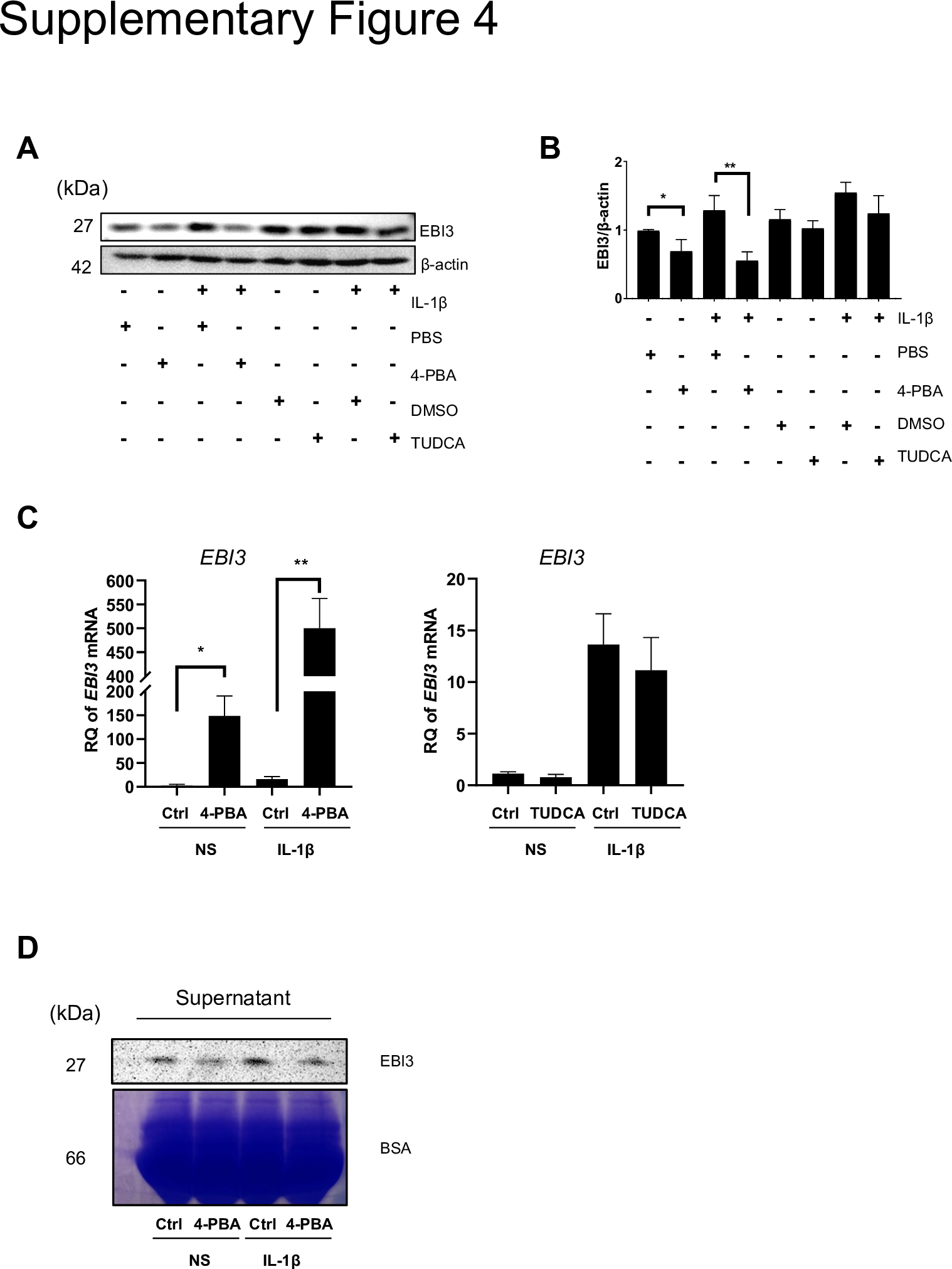

Supplement: S4 Fig — (A-D) MSCs were cultured in a monolayer in growth medium and pretreated with 4-PBA or TUDCA for 6 hrs. After that IL-1β were added, and cells were cultured for 36 hrs. (A) Whole-cell lysates were analyzed for EBI3 by Western blotting. (B) Densitometric analysis of A was performed, and the data were normalized to β-actin. (C) Total RNA was collected after the indicated treatment. EBI3 mRNA levels were measured by RT-qPCR. Quantified data are expressed as the mean ± SD (each n = 3). * = P < 0.05; ** = P < 0.01 by Student’s unpaired 2-tailed t-test. (D) Supernatants were analyzed for EBI3 by CBB and Western blotting. Results are representative of 3 independent experiments with similar findings. (TIF) [file pone.0279584.s004.tif]

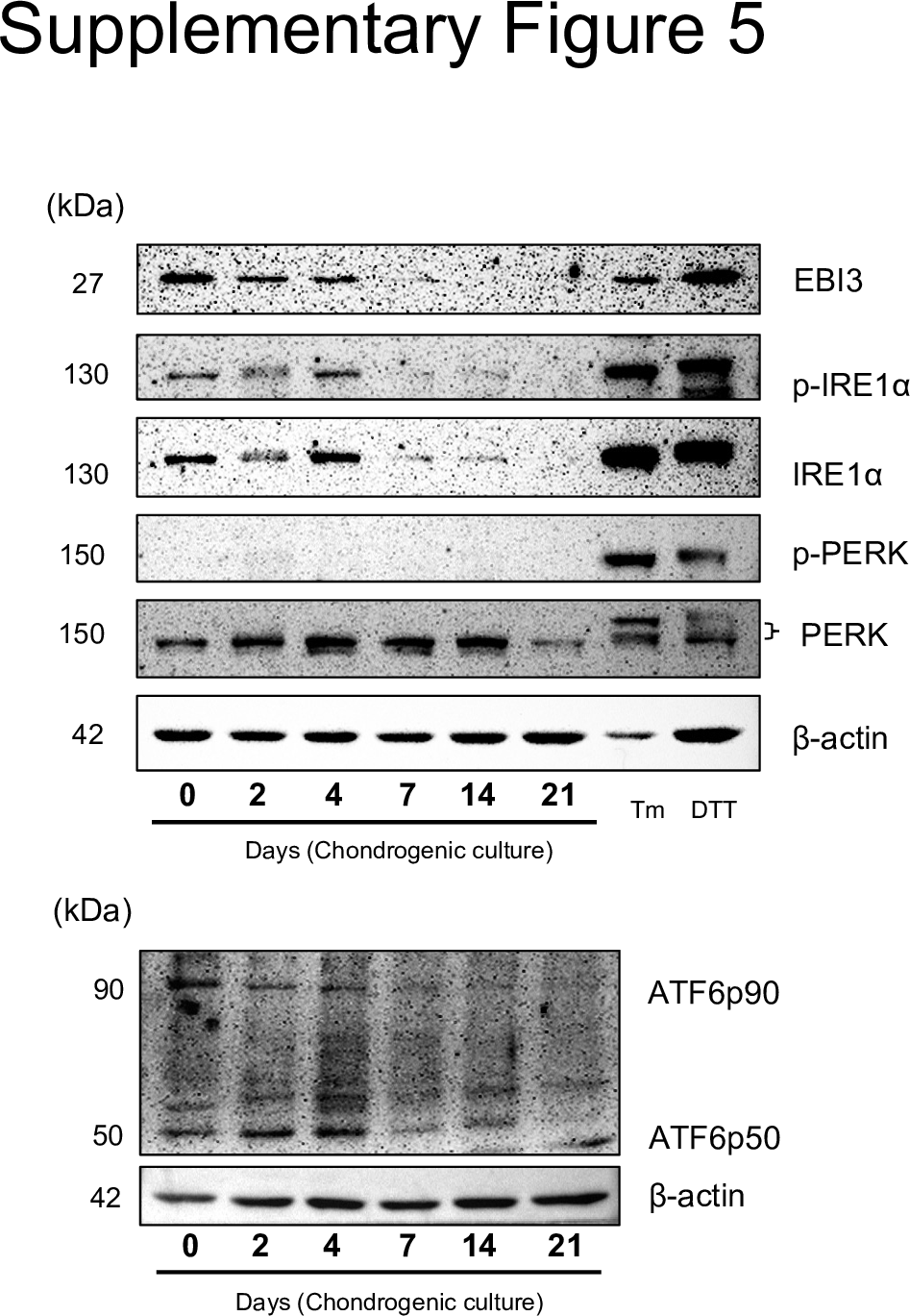

Supplement: S5 Fig — MSCs were cultured in chondrogenic medium in pellet form. At each time, whole-cell lysates were prepared at the indicated times and underwent Western blotting to detect EBI3, p-IRE1α, IRE1α, p-PERK, PERK, ATF6p50, and β-actin. β-actin was used as a loading control. Results are representative of 3 independent experiments with similar findings. (TIF) [file pone.0279584.s005.tif]

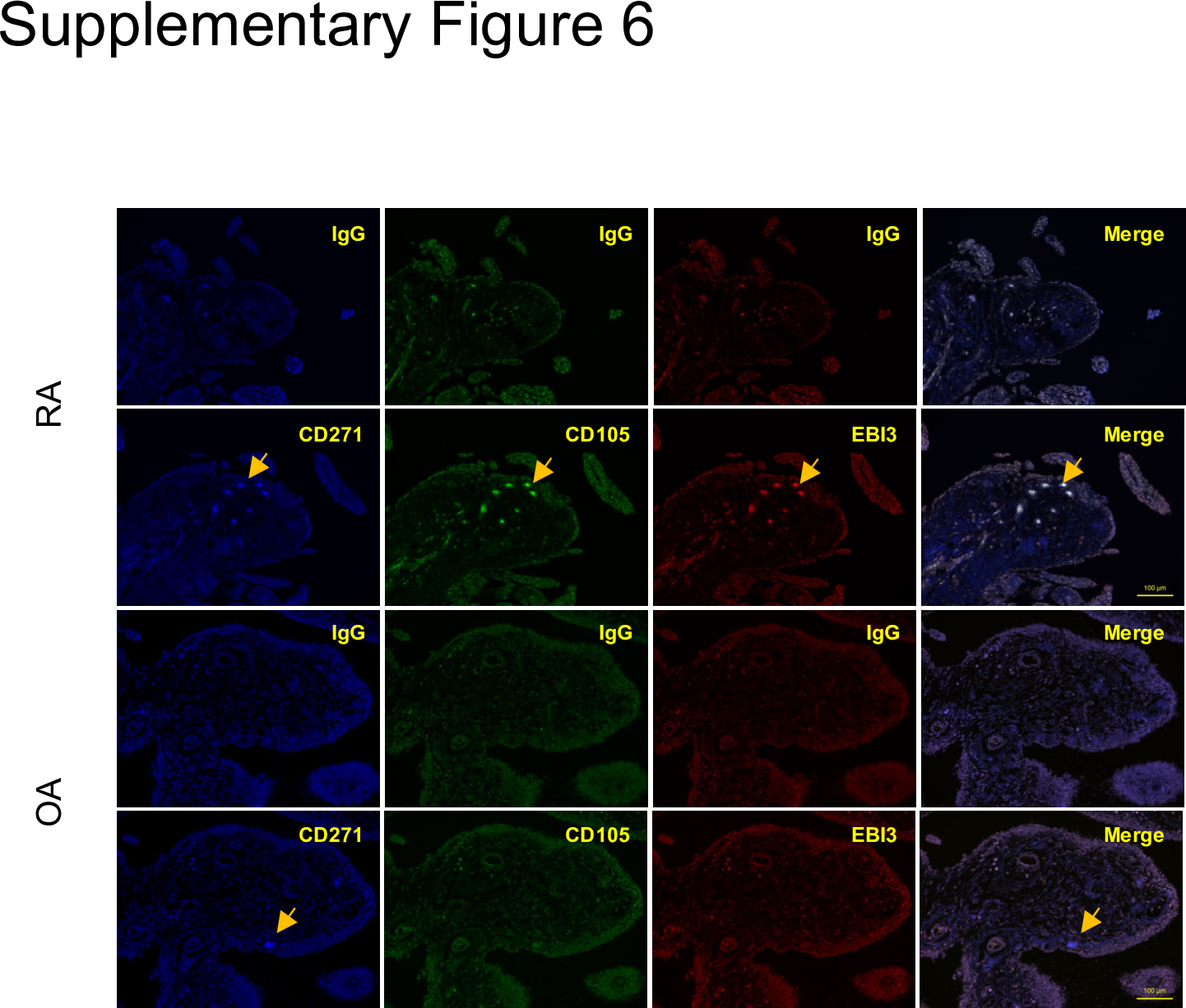

Supplement: S6 Fig — Synovial specimens from RA patients (n = 3) and OA patients (n = 3) were stained by immunofluorescence using specific antibodies against CD271, CD105, and EBI3. Results are representative of 3 independent experiments with similar findings. Scale bar, 100 μm. (TIF) [file pone.0279584.s006.tif]

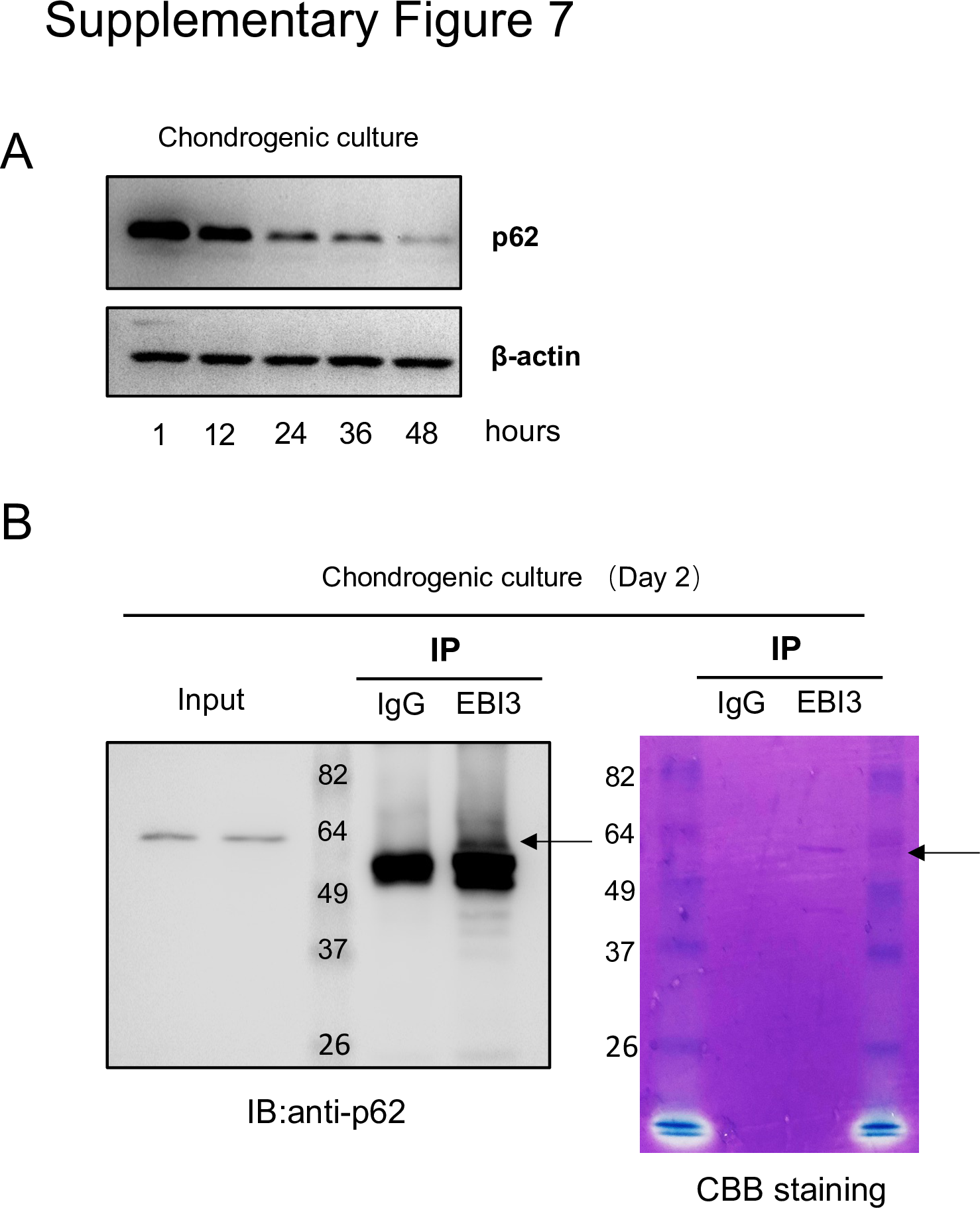

Supplement: S7 Fig — MSCs were pellet-culured in chondrogenic medium and protein was collected at indicated time points. (A) Western blotting was performed to detect p62 and β-actin. (B) Protein was immunoprecipitated by EBI3 antibody and protein A agarose. Western blotting and CBB were performed to detect p62. Results are representative of 2 independent experiments with similar findings. (TIF) [file pone.0279584.s007.tif]

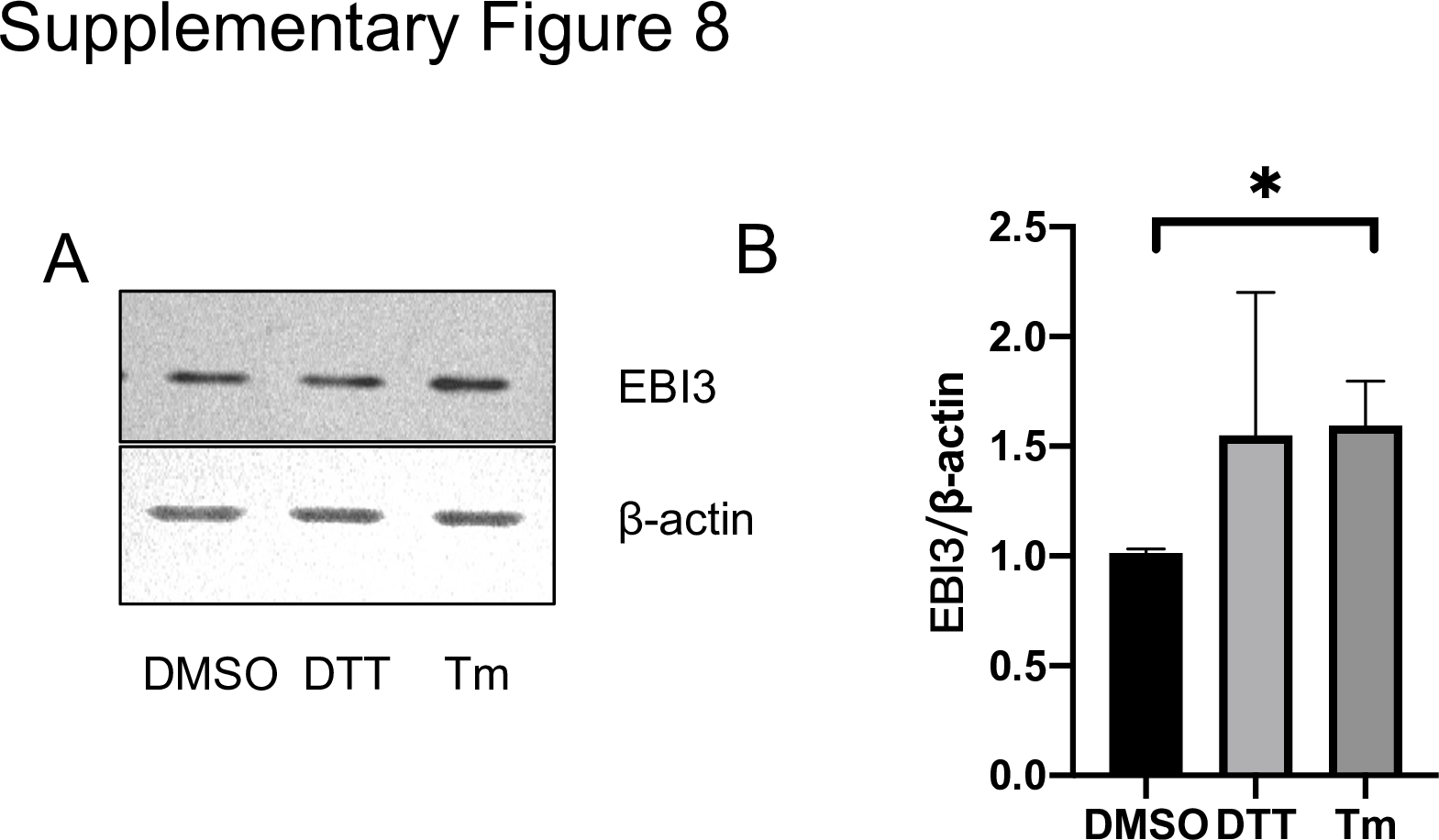

Supplement: S8 Fig — MSCs were cultured in monolayer in growth medium. After treatment of MSCs with DTT (5 mM) and Tunicamycin (5 μg/ml) for 9 hours, whole-cell lysates were collected and underwent Western blotting to detect EBI3 and β-actin. Results are representative of 4 independent experiments with similar findings. (B) Densitometric analysis of (A) was performed, and the data were normalized to β-actin. Quantified data are expressed as the mean ± SD (n = 4). * = P<0.05 by Student’s unpaired 2-tailed t-test. (TIF) [file pone.0279584.s008.tif]

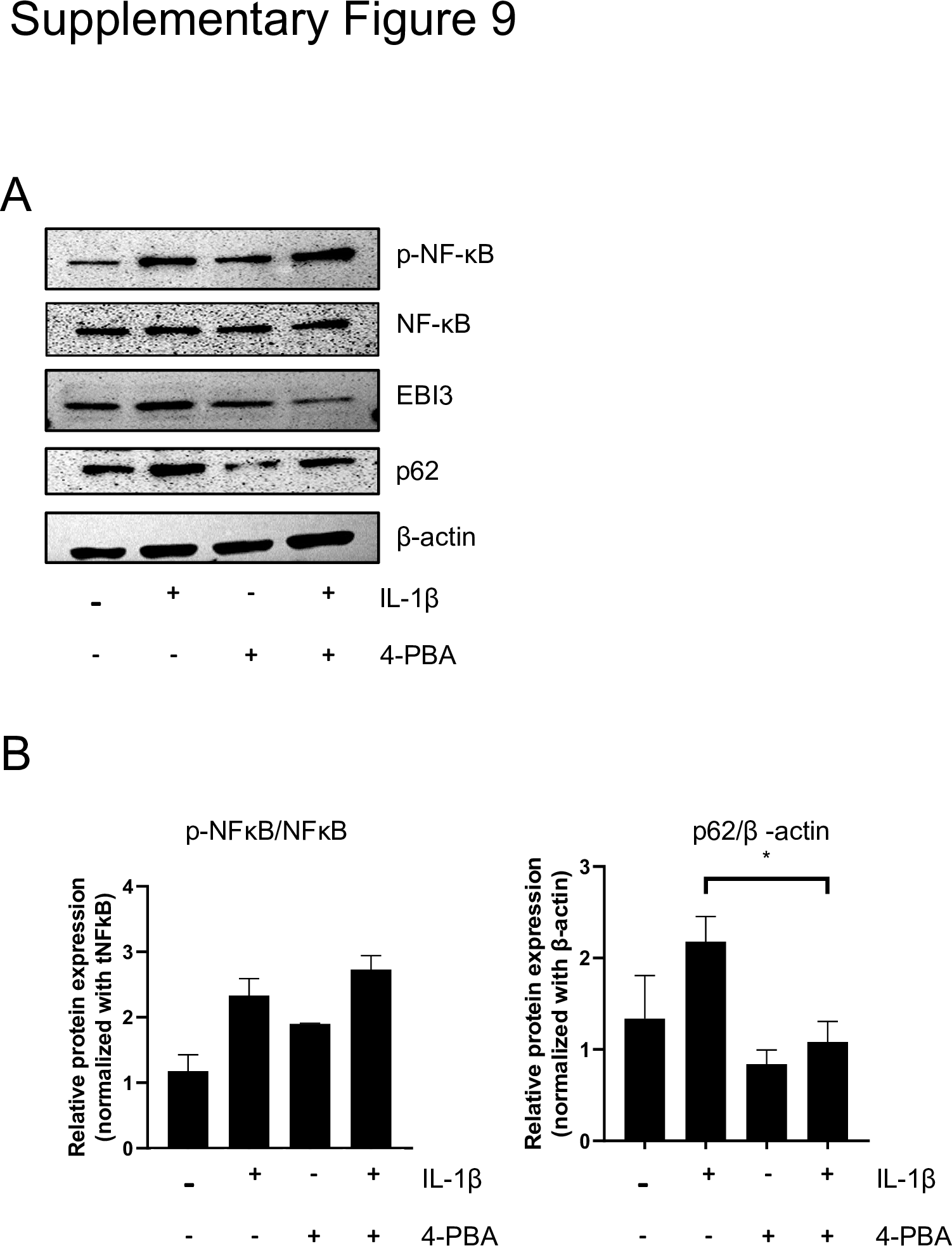

Supplement: S9 Fig — MSCs were cultured in a monolayer in growth medium and pretreated with 4-PBA for 6 hrs. After that IL-1β were added, and cells were cultured for 36 hrs. (A) Whole-cell lysates were analyzed for phosphorylation of NF-κB, NF-κB, EBI3, and p62 by Western blotting. (B) Densitometric analysis of A was performed, and the data were normalized to NF-κB and β-actin. Quantified data are expressed as the mean ± SD (n = 2). * = P<0.05 by Dunnett’s multiple comparison test. (TIF) [file pone.0279584.s009.tif]

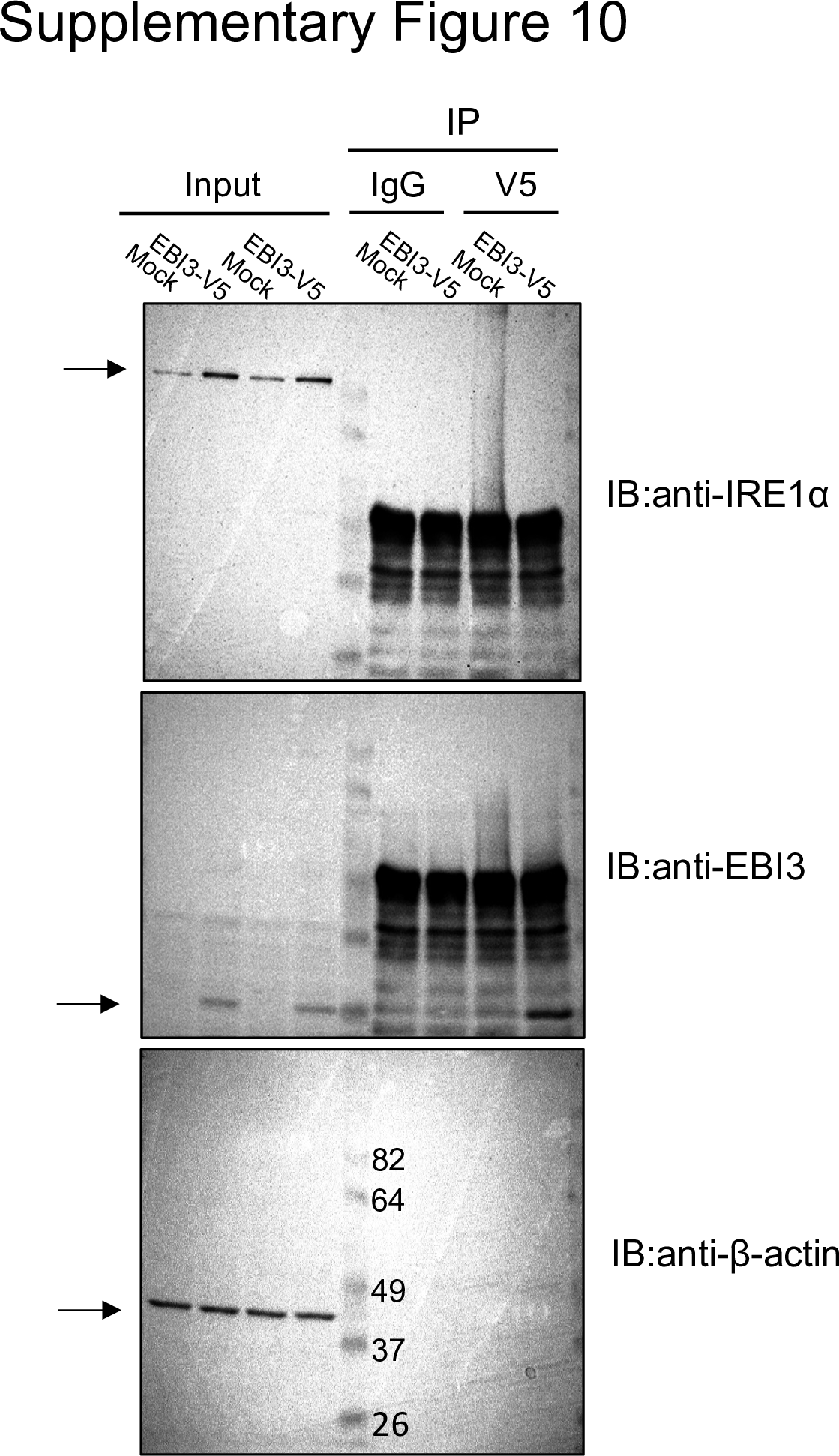

Supplement: S10 Fig — MSCs were cultured in a monolayer in growth medium and transfected with pEF6-EBI3-V5 plasmid DNA for 48 hrs. After that IL-1β were added, and cells were cultured for 24 hrs. Whole-cell lysates were immunoprecipitated by anti-V5-tag-pAb-agarose and were analyzed for IRE1α and EBI3 by Western blotting. Results are representative of 3 independent experiments with similar findings. (TIF) [file pone.0279584.s010.tif]

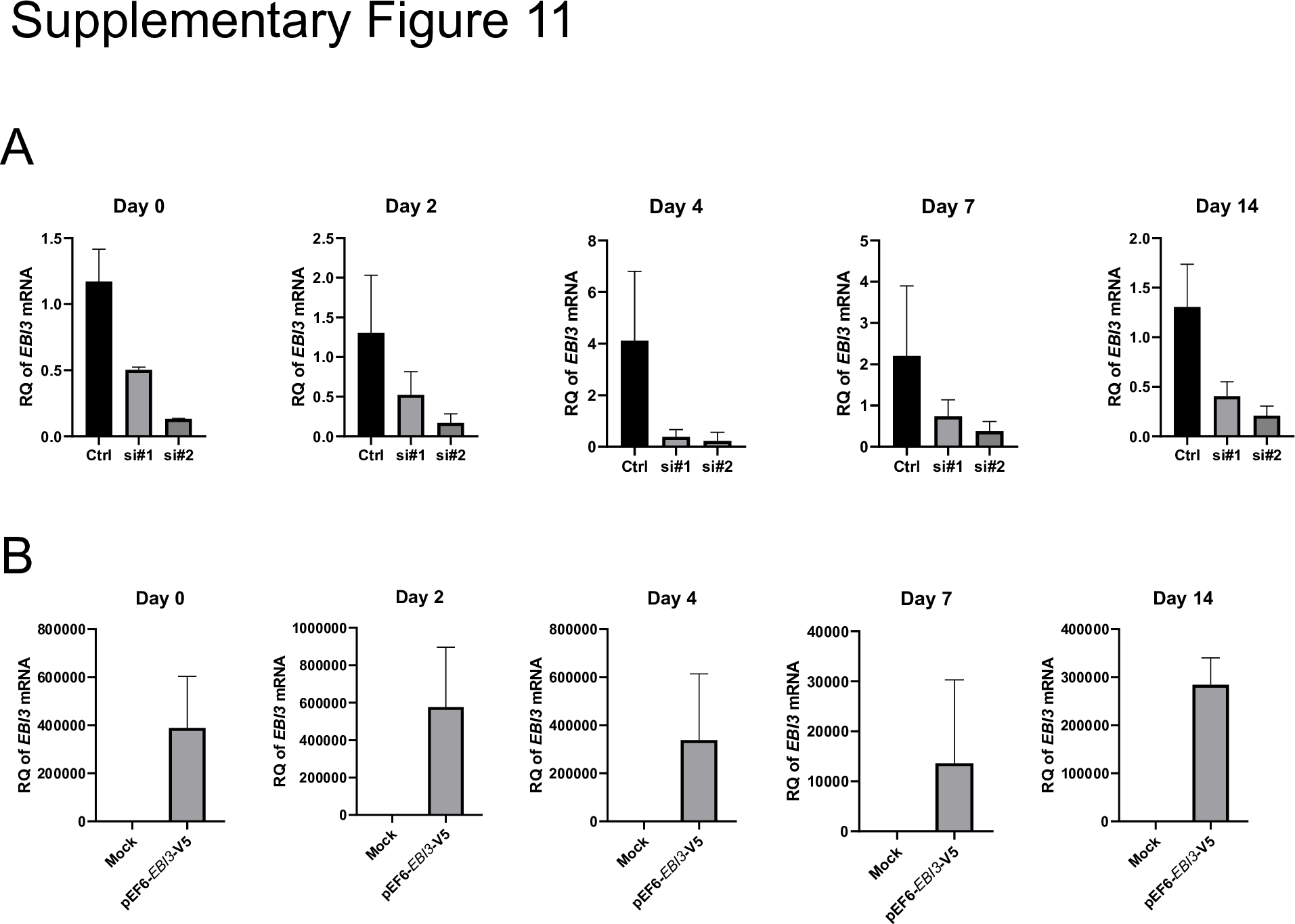

Supplement: S11 Fig — (A) MSCs were transfected with control siRNA or 2 different EBI3 siRNAs (si#1 and si#2) and pellet- cultured. (B) MSCs were transfected with empty or pEF6-EBI3-V5 plasmid DNA and pellet-cultured. The EBI3 mRNA levels in transfected pellets were determined by RT-qPCR at the indicated time point. Quantified data are expressed as the mean ± SD (each n = 2~3). (TIF) [file pone.0279584.s011.tif]

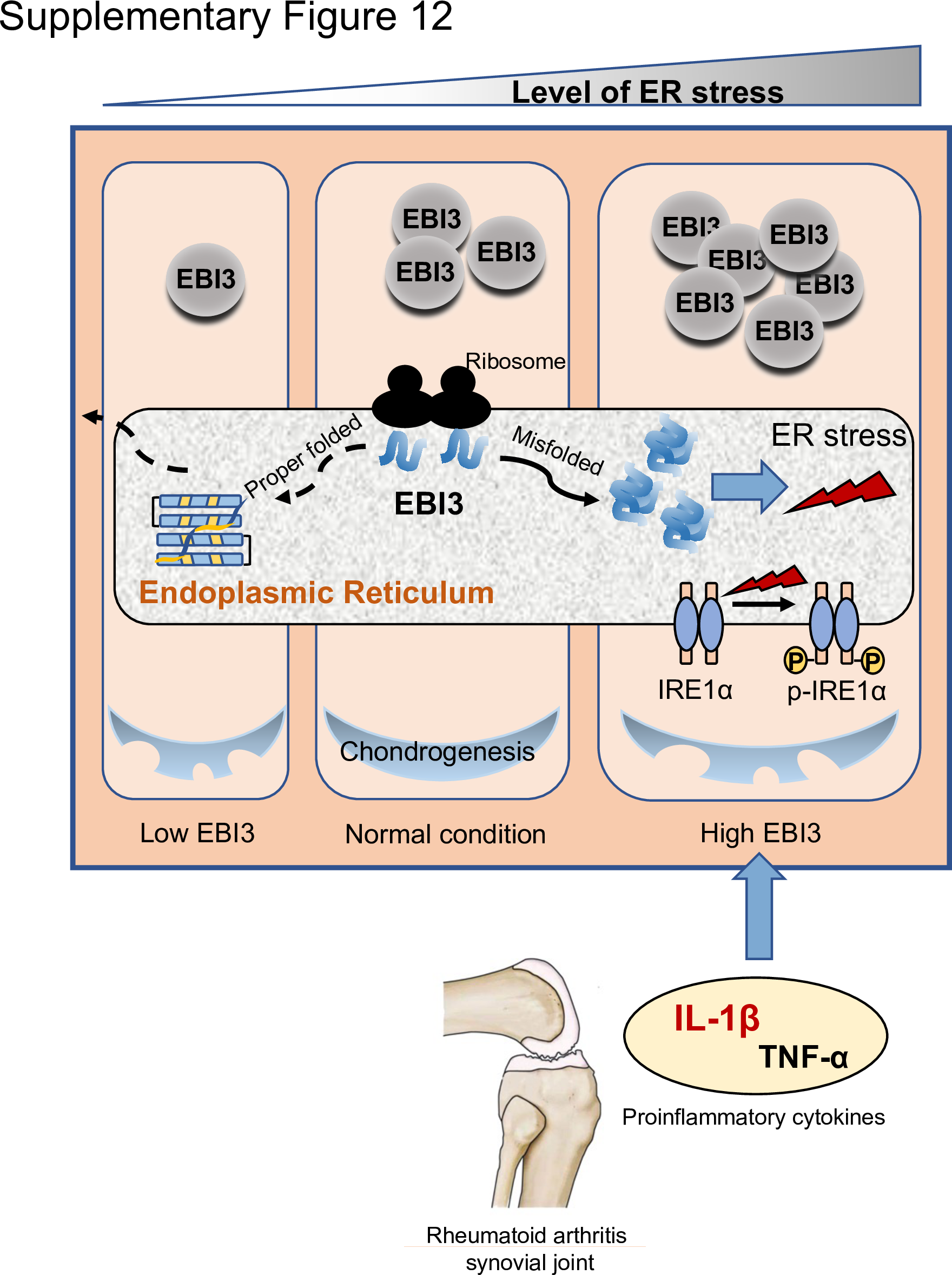

Supplement: S12 Fig — In normal condition, EBI3 exists in the early stage and maintain the ER stress. While low ER stress caused by EBI3 knockdown or excessive ER stress induced by overexpression of EBI3 result in the inhibition of chondrogenesis. In RA condition, high expression of EBI3 induced by inflammatory cytokines contributes to excessive ER stress and suppression of chondrogenesis. (TIF) [file pone.0279584.s012.tif]

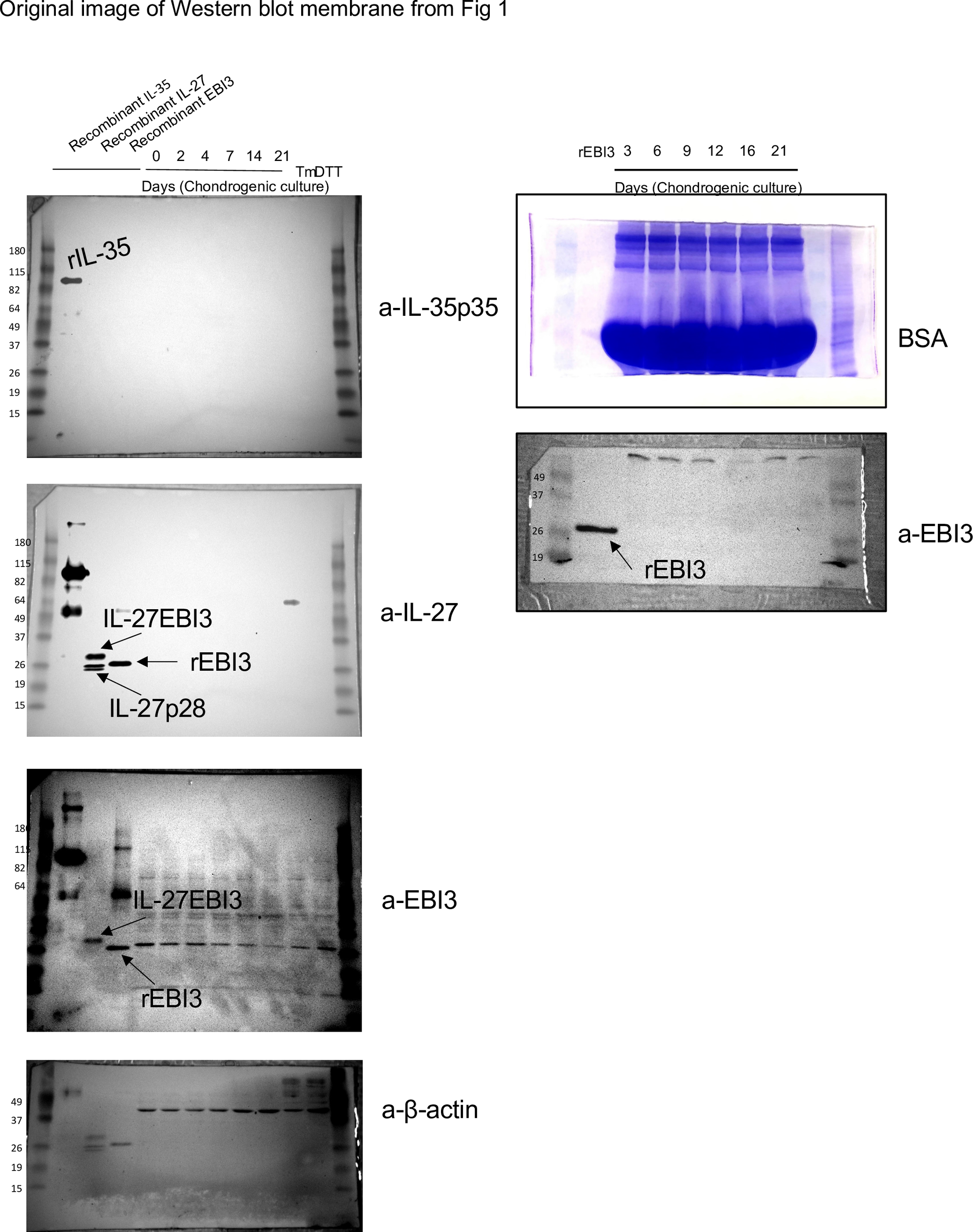

Supplement: S1 File — (ZIP) [file pone.0279584.s015.zip › S1 files/WB CBB Fig 1.tif]

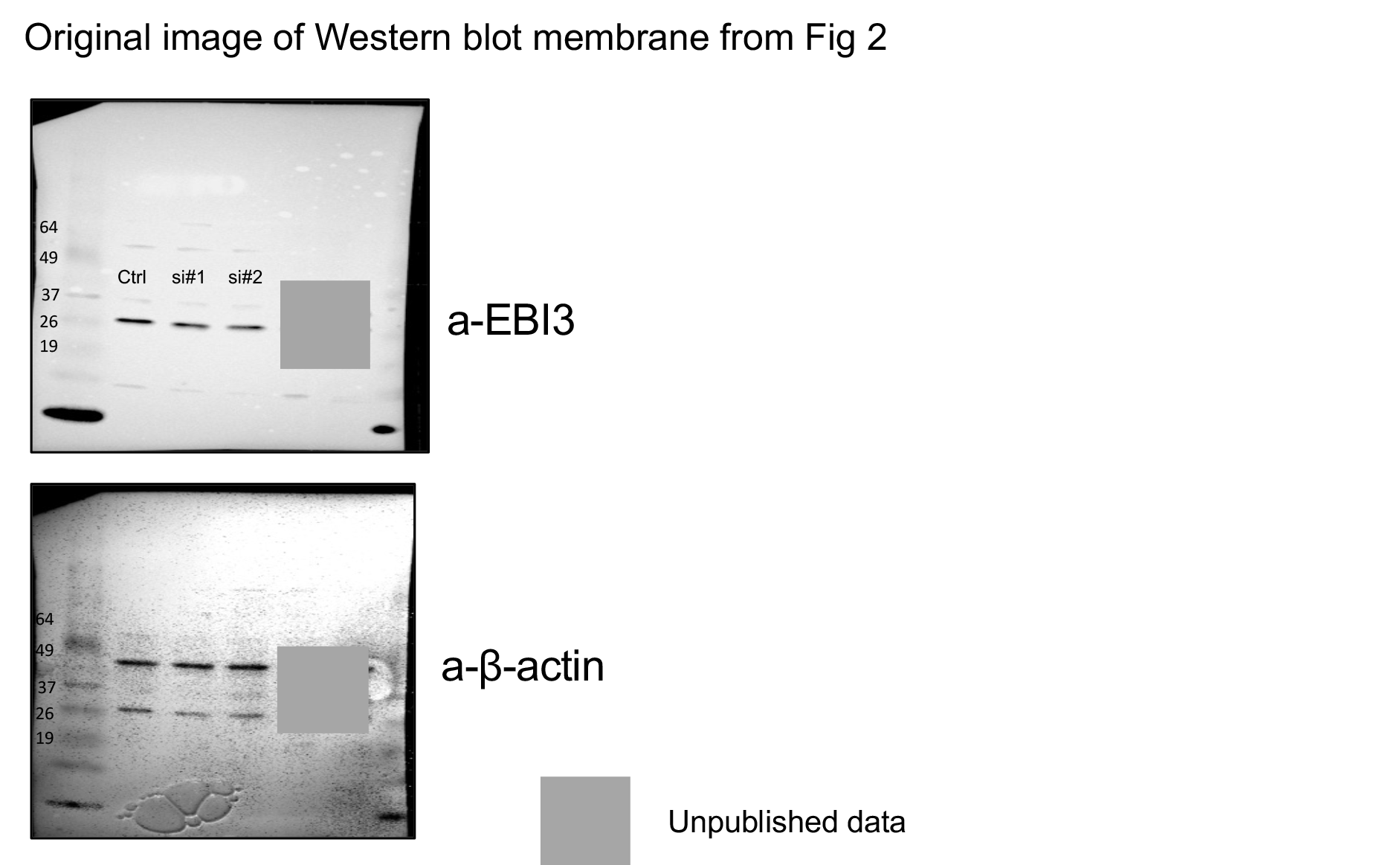

Supplement: S2 File — (ZIP) [file pone.0279584.s016.zip › S2 files/WB Fig 2.tif]

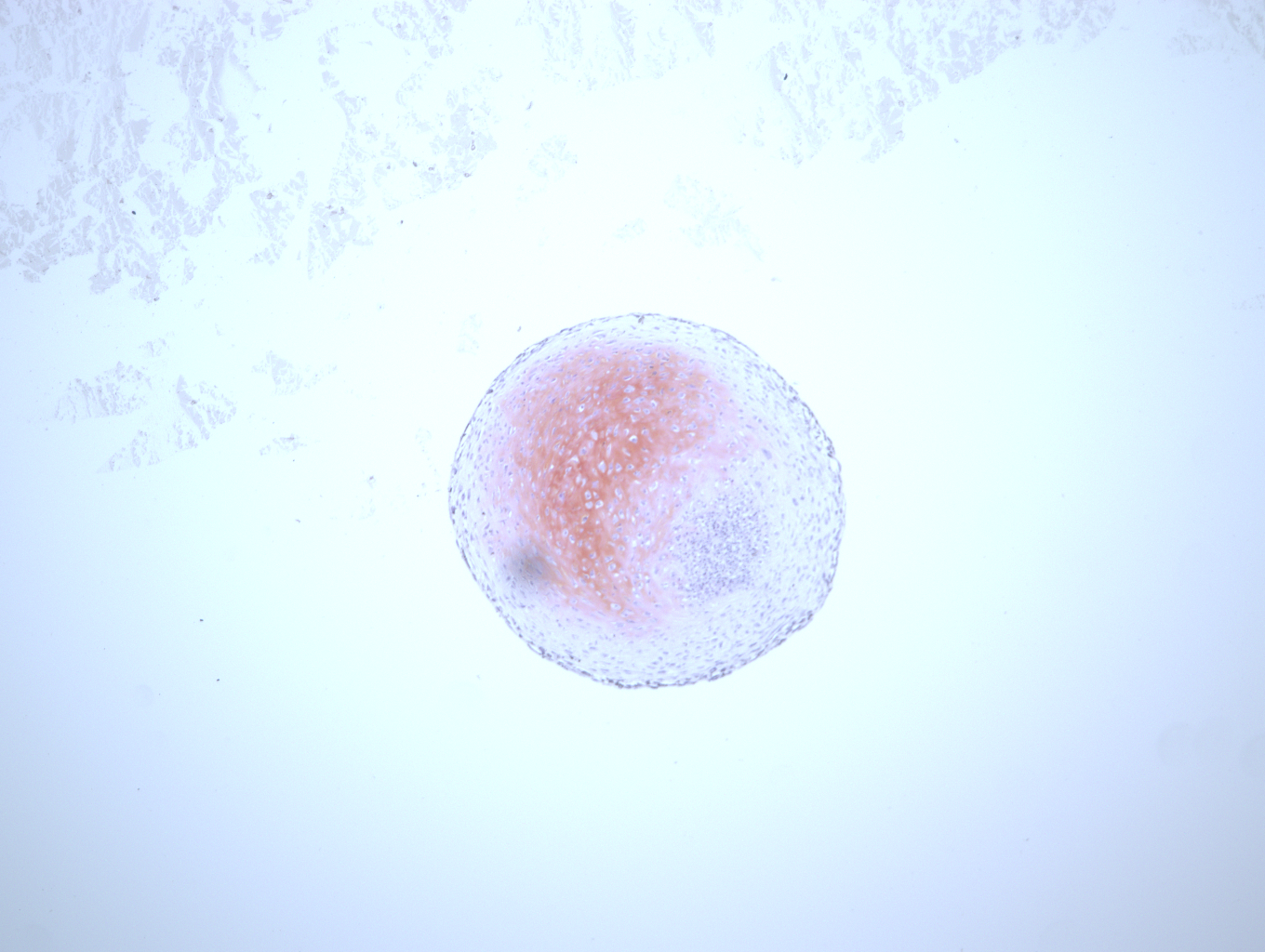

Supplement: S2 File — (ZIP) [file pone.0279584.s016.zip › S2 files/IHC/S-O/EBI3#1.TIF]

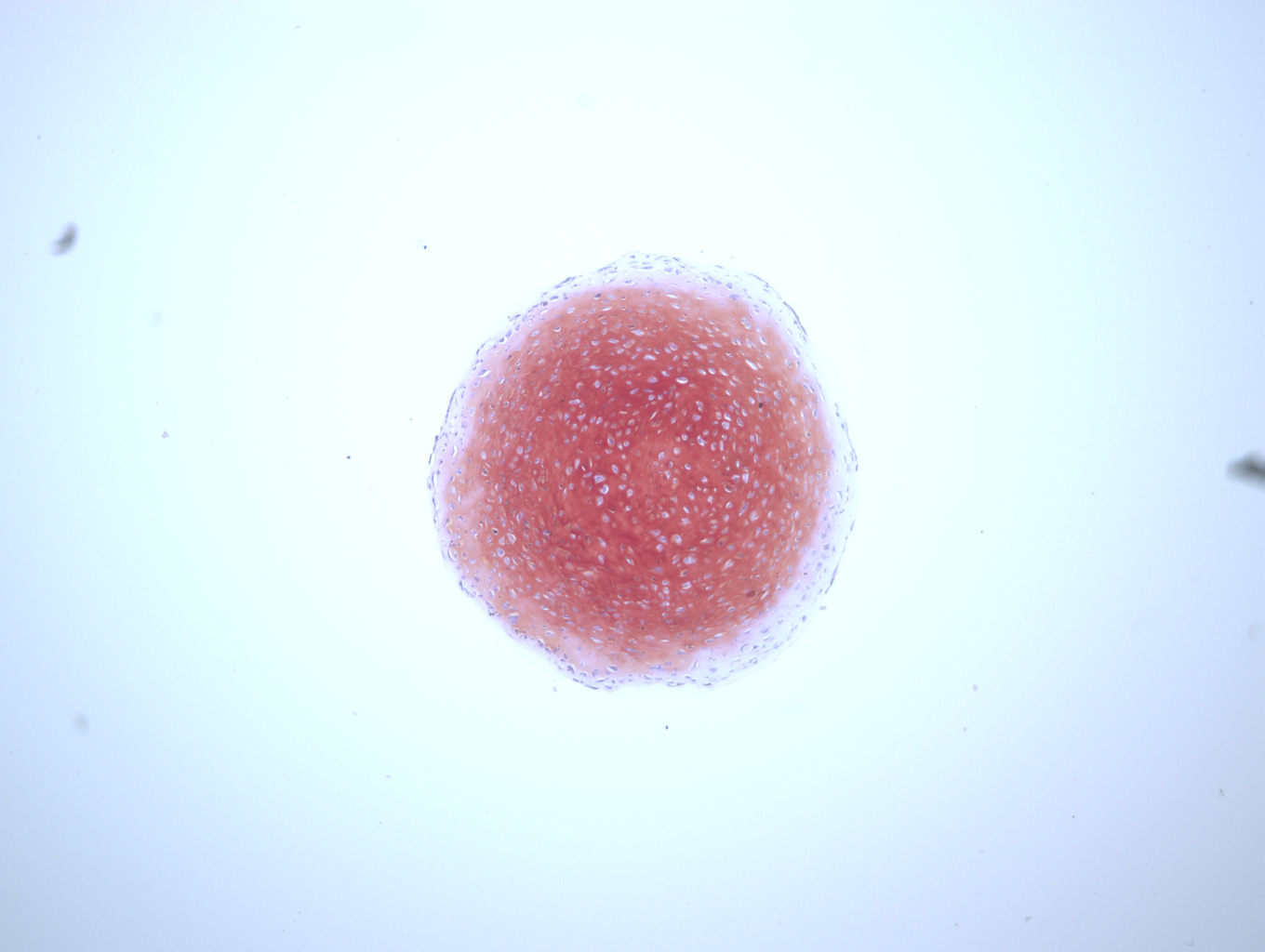

Supplement: S2 File — (ZIP) [file pone.0279584.s016.zip › S2 files/IHC/S-O/siRNA control.TIF]

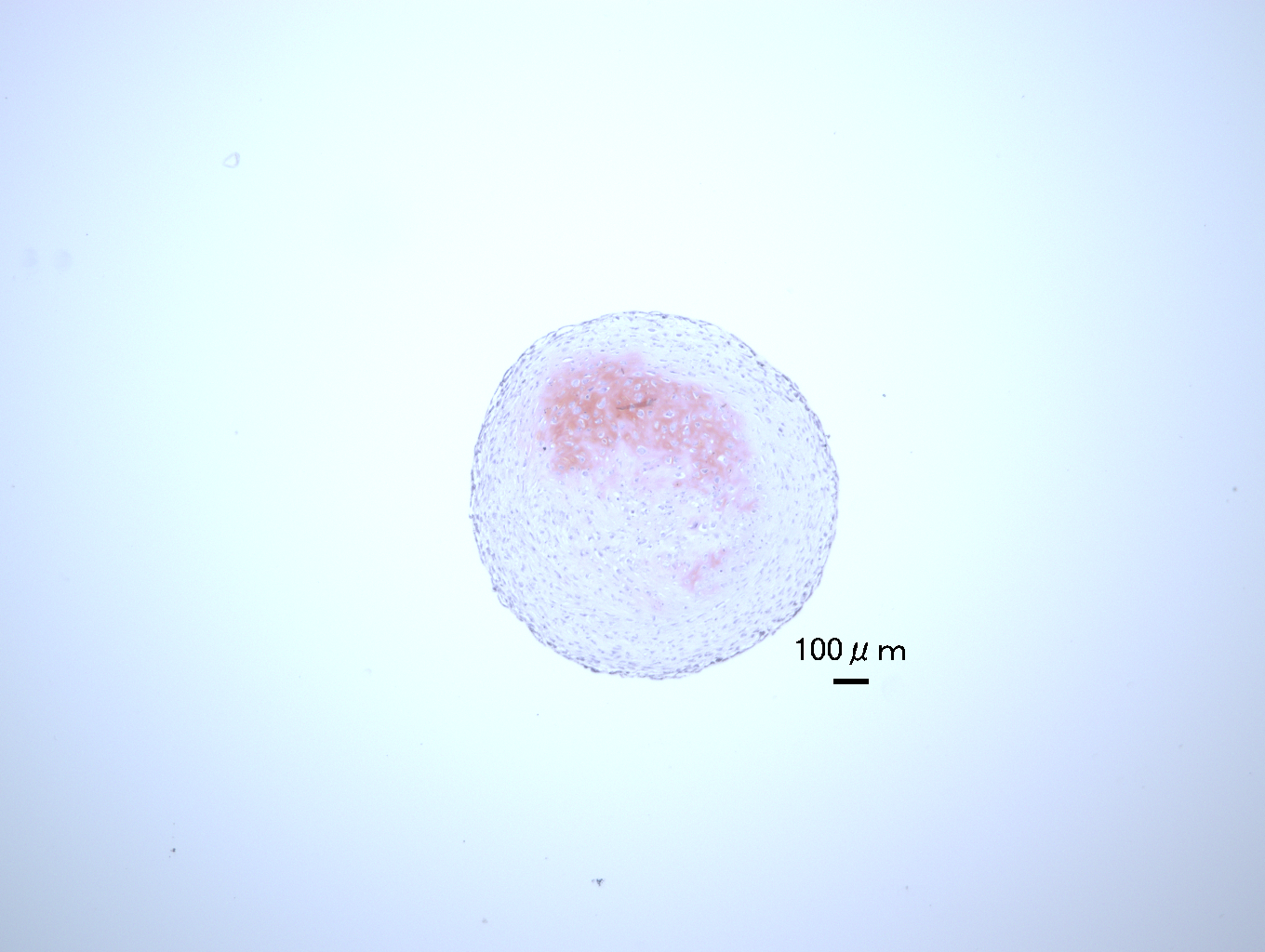

Supplement: S2 File — (ZIP) [file pone.0279584.s016.zip › S2 files/IHC/S-O/EBI3#2.TIF]

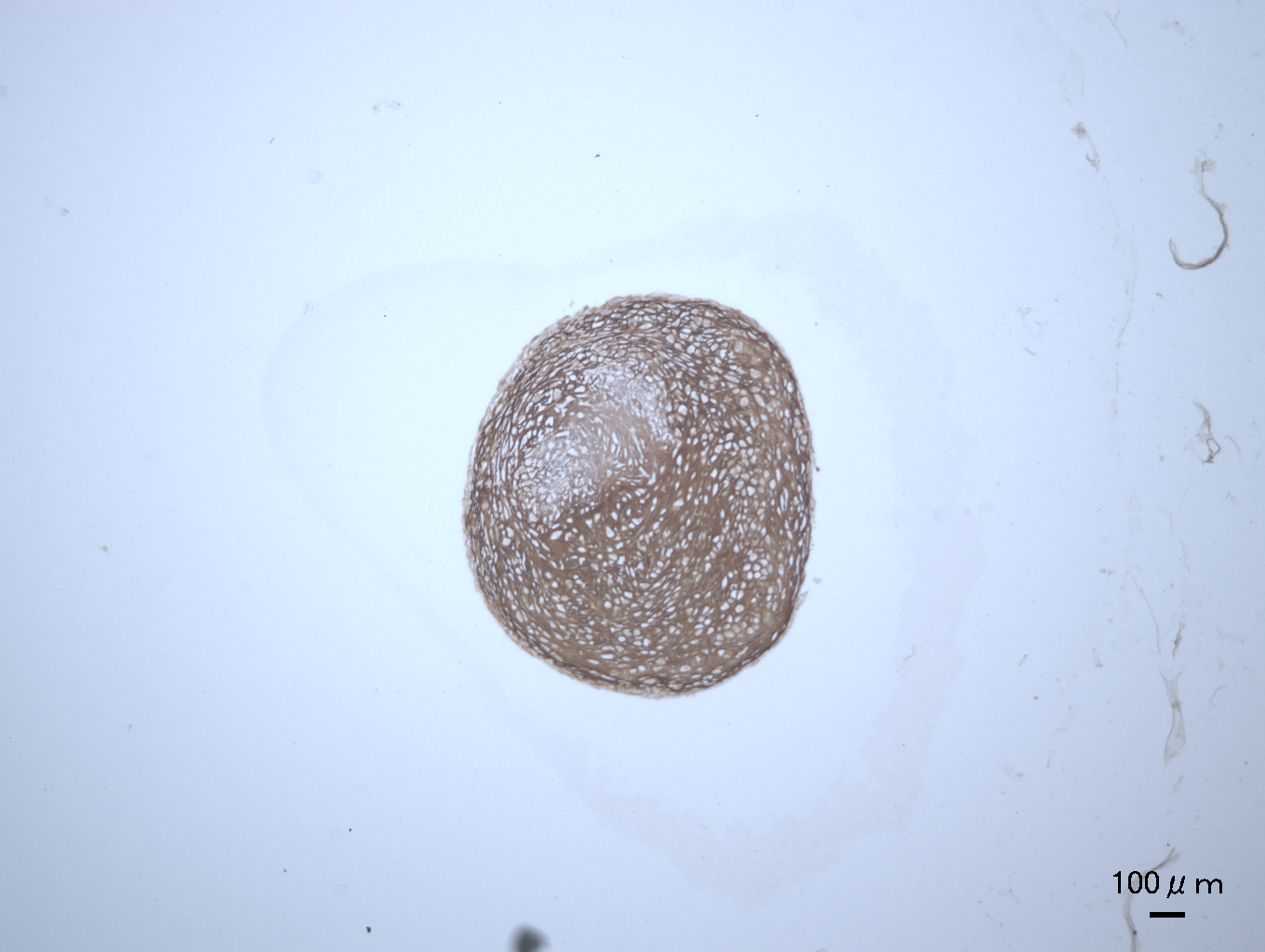

Supplement: S2 File — (ZIP) [file pone.0279584.s016.zip › S2 files/IHC/COL2/EBI3#1.TIF]

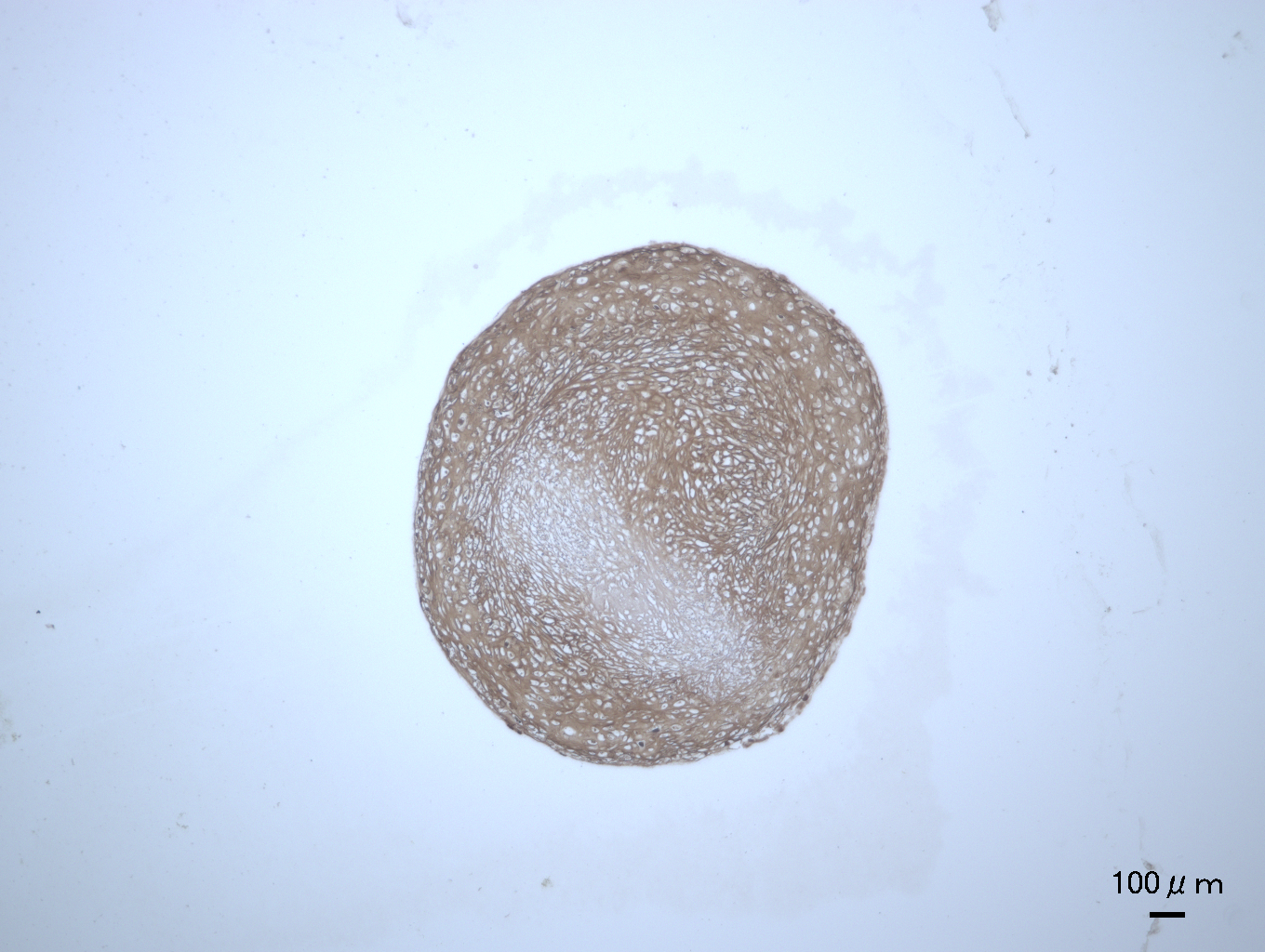

Supplement: S2 File — (ZIP) [file pone.0279584.s016.zip › S2 files/IHC/COL2/siRNA control.TIF]

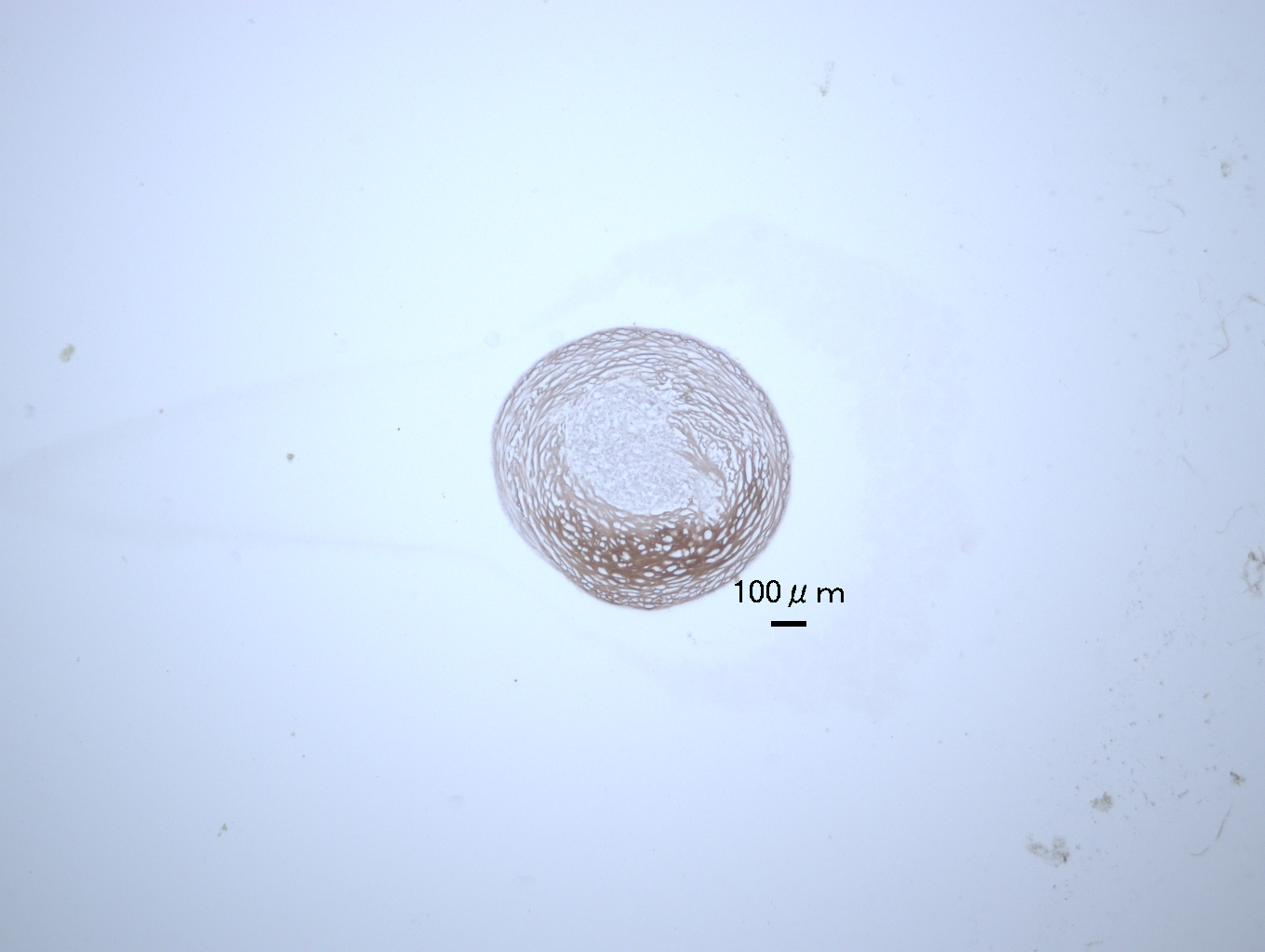

Supplement: S2 File — (ZIP) [file pone.0279584.s016.zip › S2 files/IHC/COL2/EBI3#2.TIF]

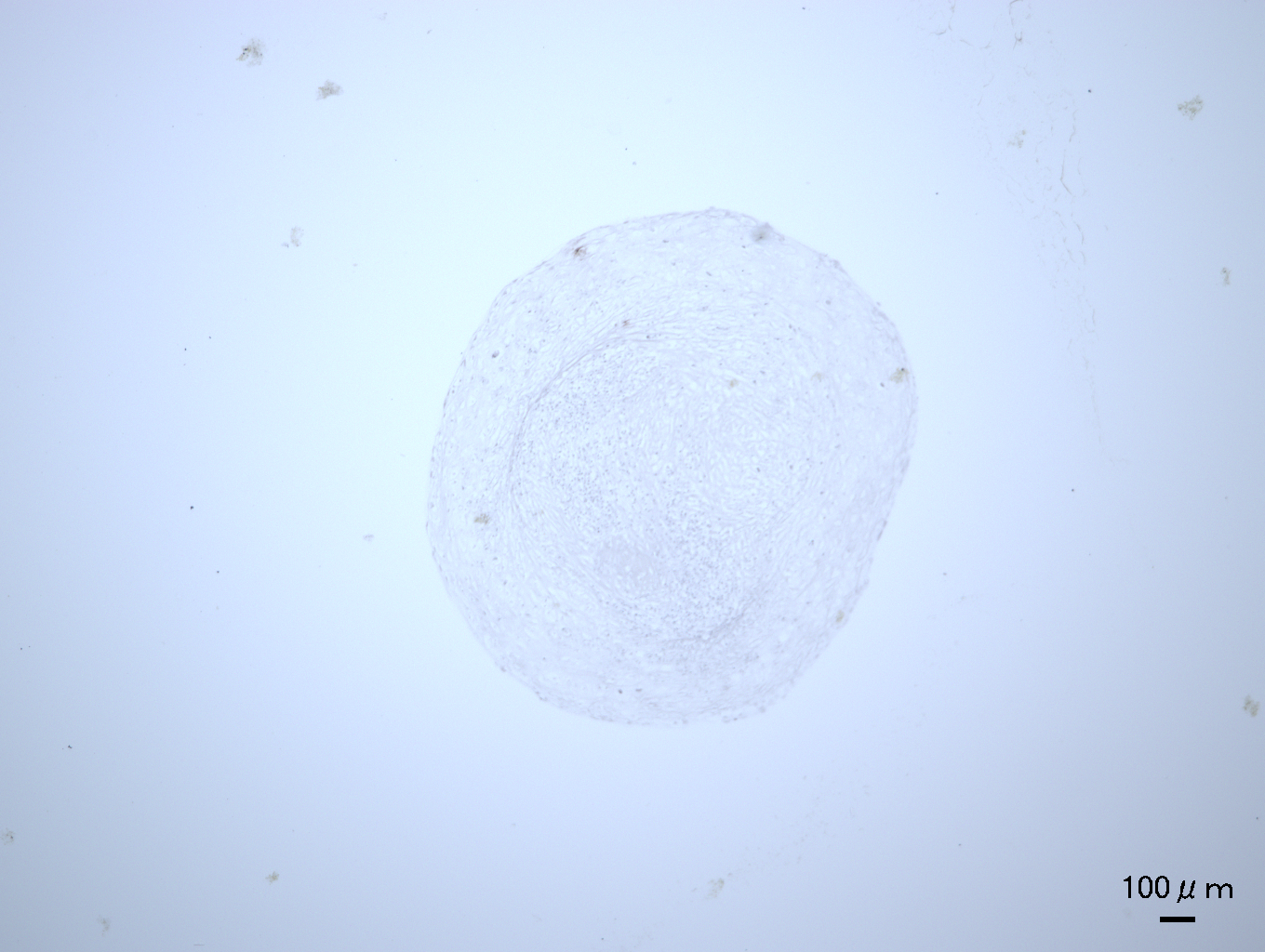

Supplement: S2 File — (ZIP) [file pone.0279584.s016.zip › S2 files/IHC/COL2/SiRNA control IgG.TIF]

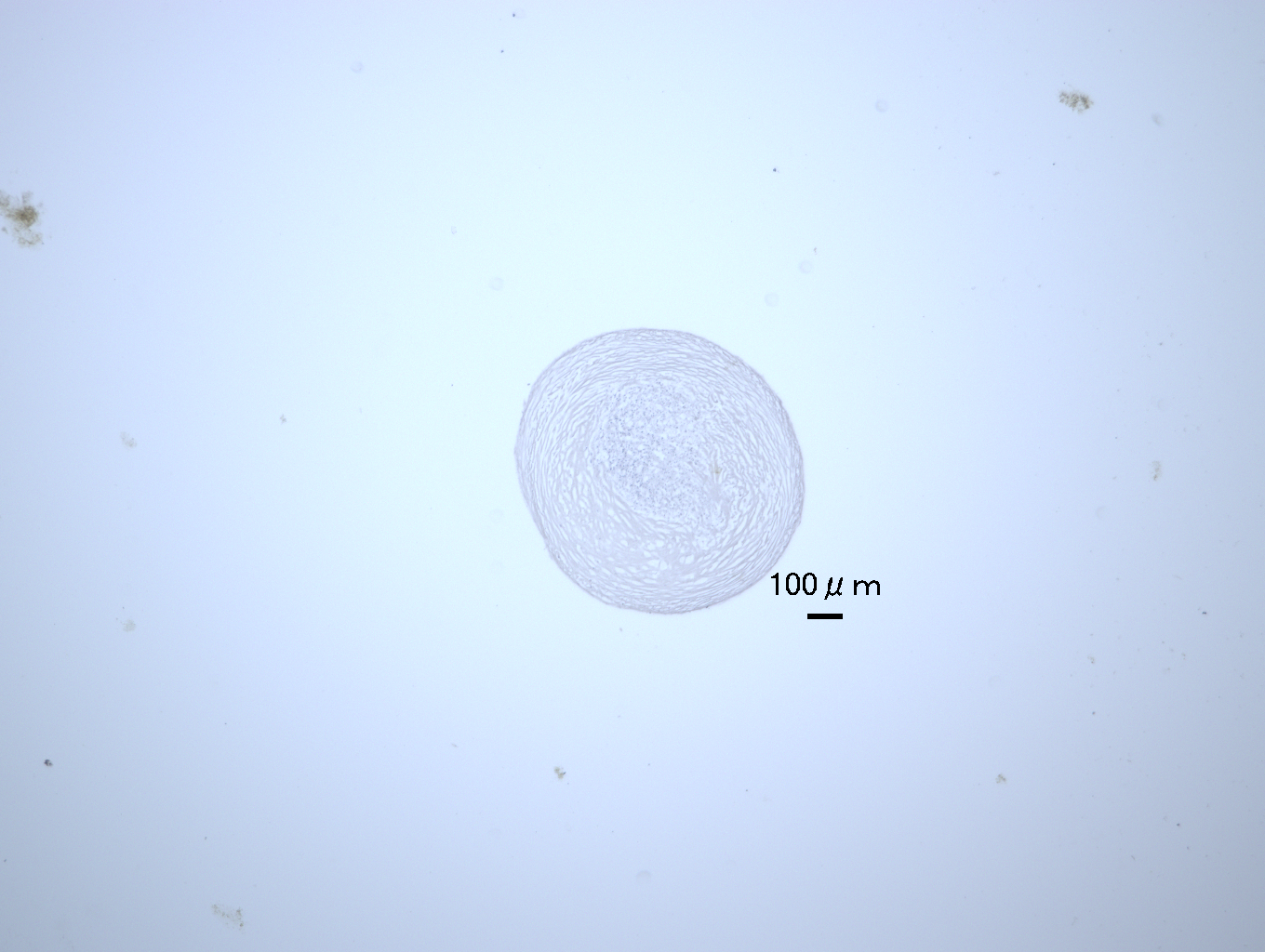

Supplement: S2 File — (ZIP) [file pone.0279584.s016.zip › S2 files/IHC/COL2/EBI3#2 IgG.TIF]

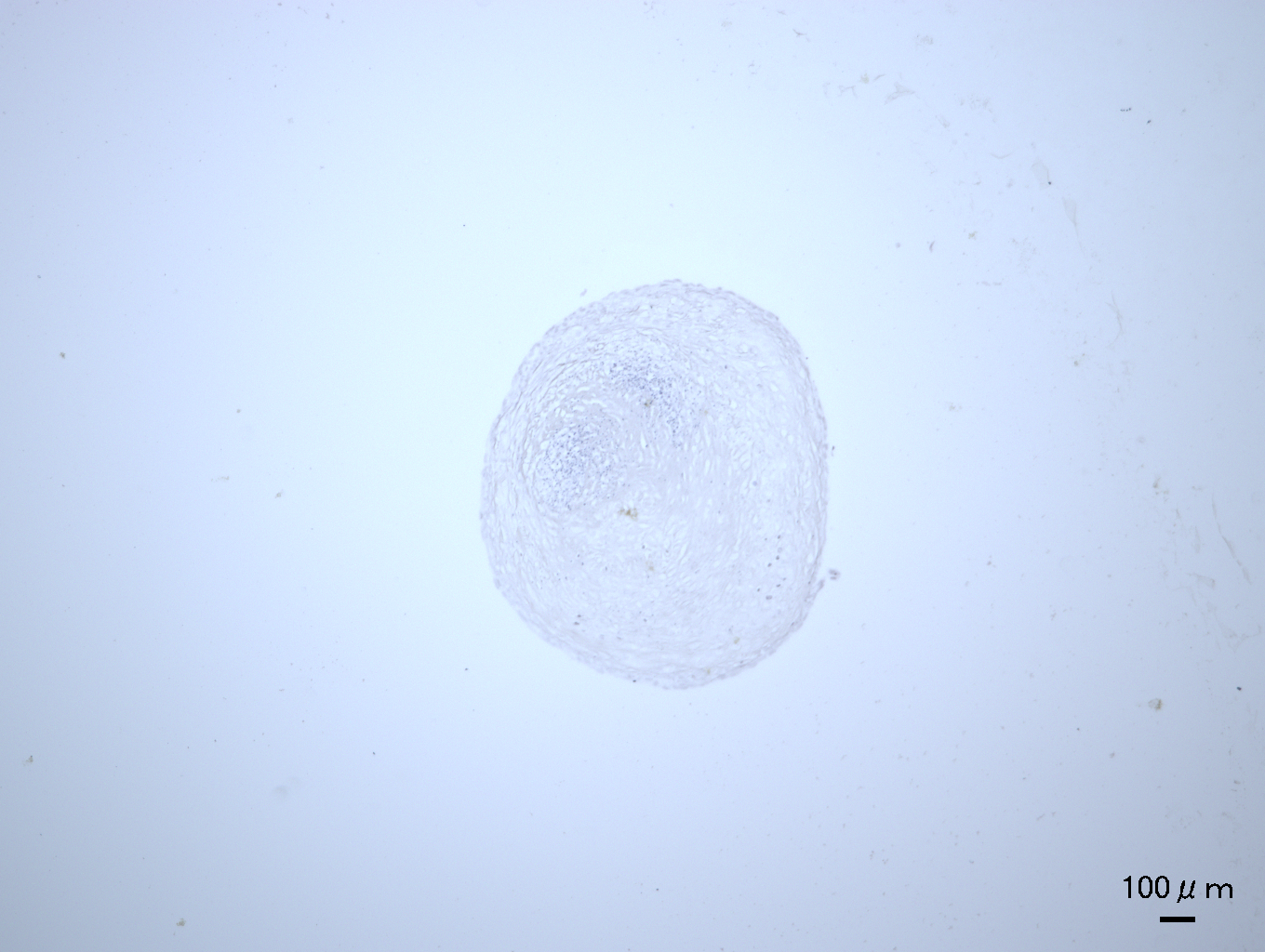

Supplement: S2 File — (ZIP) [file pone.0279584.s016.zip › S2 files/IHC/COL2/EBI3#1 IgG.TIF]

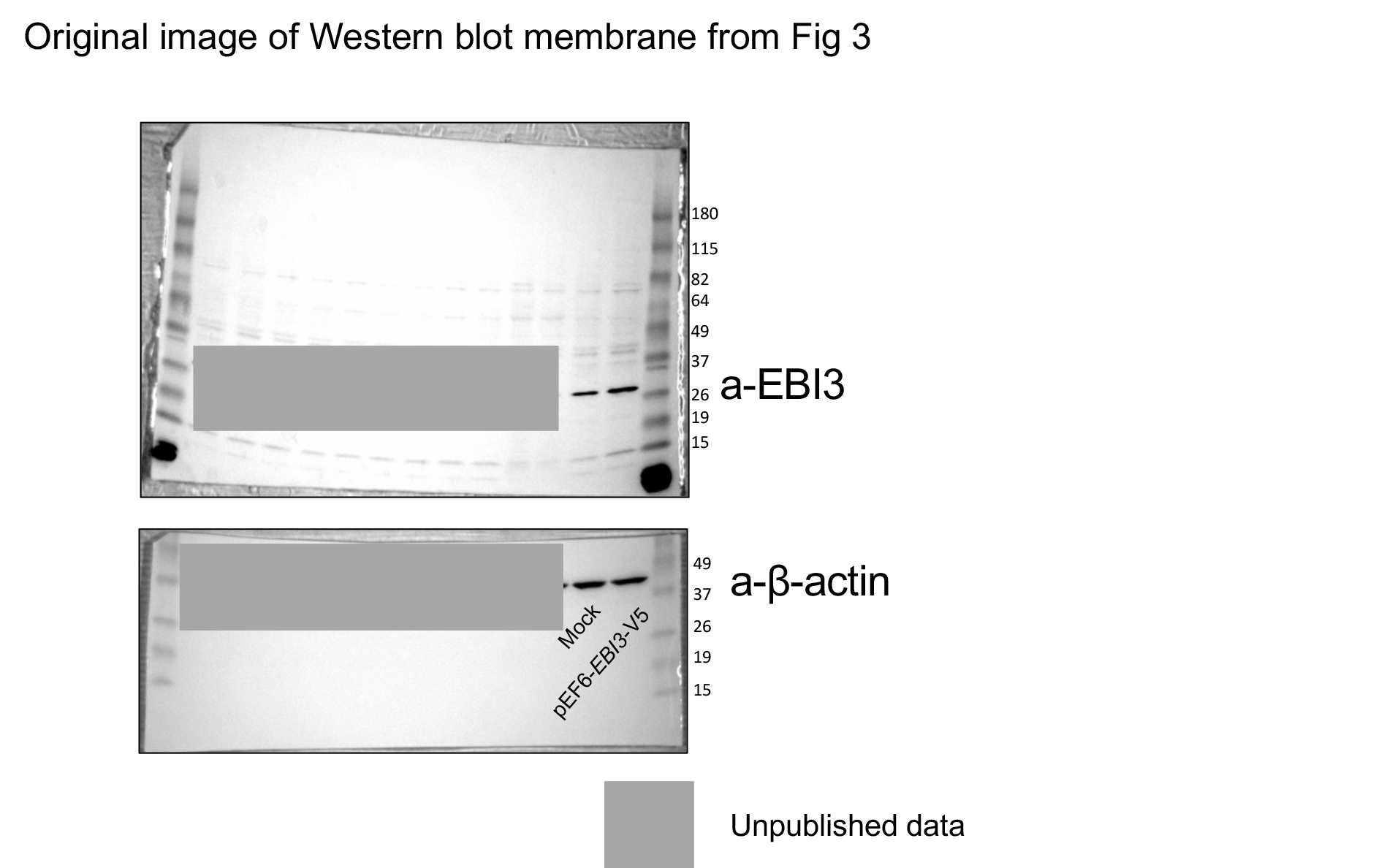

Supplement: S3 File — (ZIP) [file pone.0279584.s017.zip › S3 files/WB Fig 3.tif]

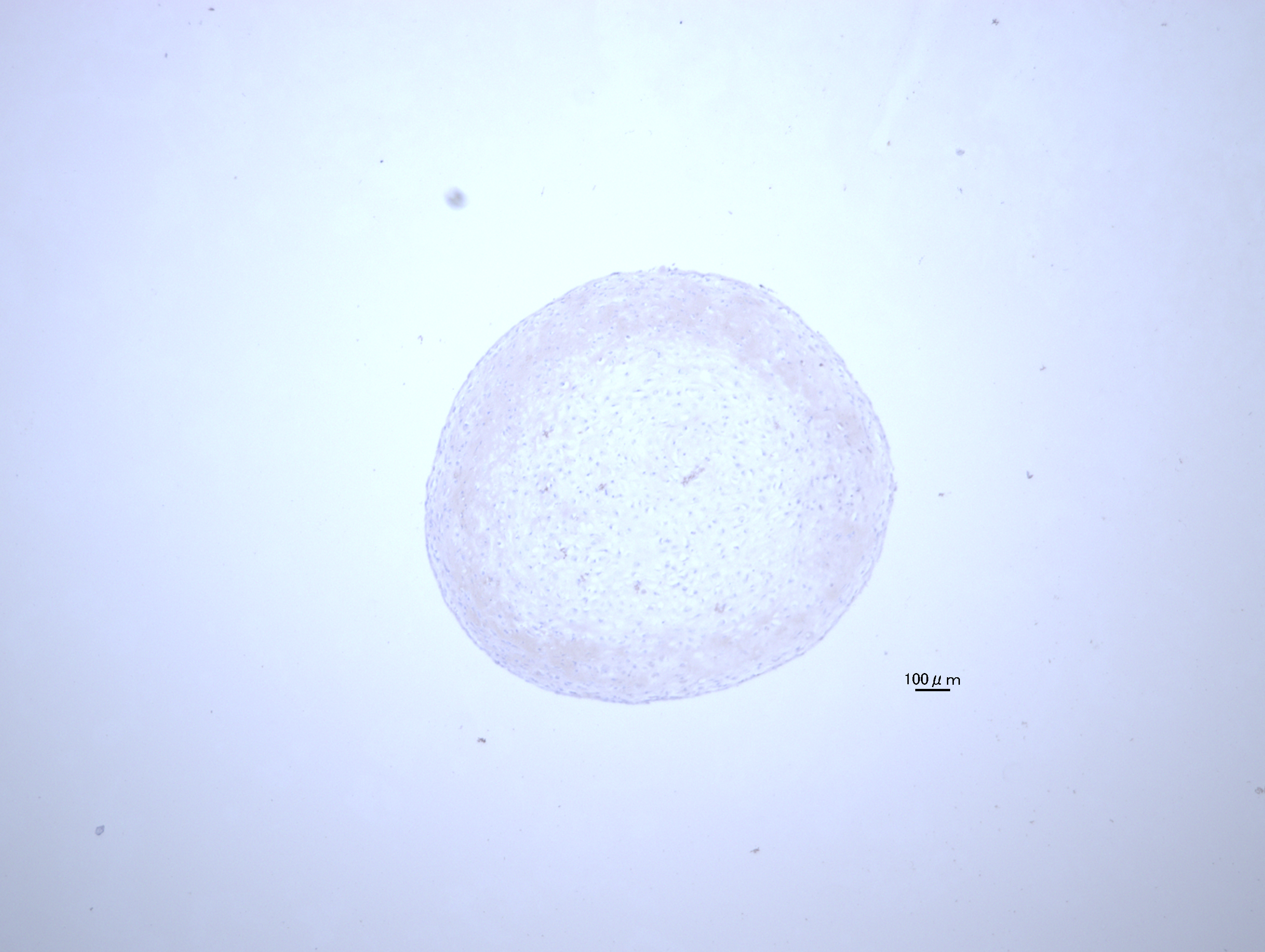

Supplement: S3 File — (ZIP) [file pone.0279584.s017.zip › S3 files/IHC/S-O/pEF6-EBI3-V5.tif]

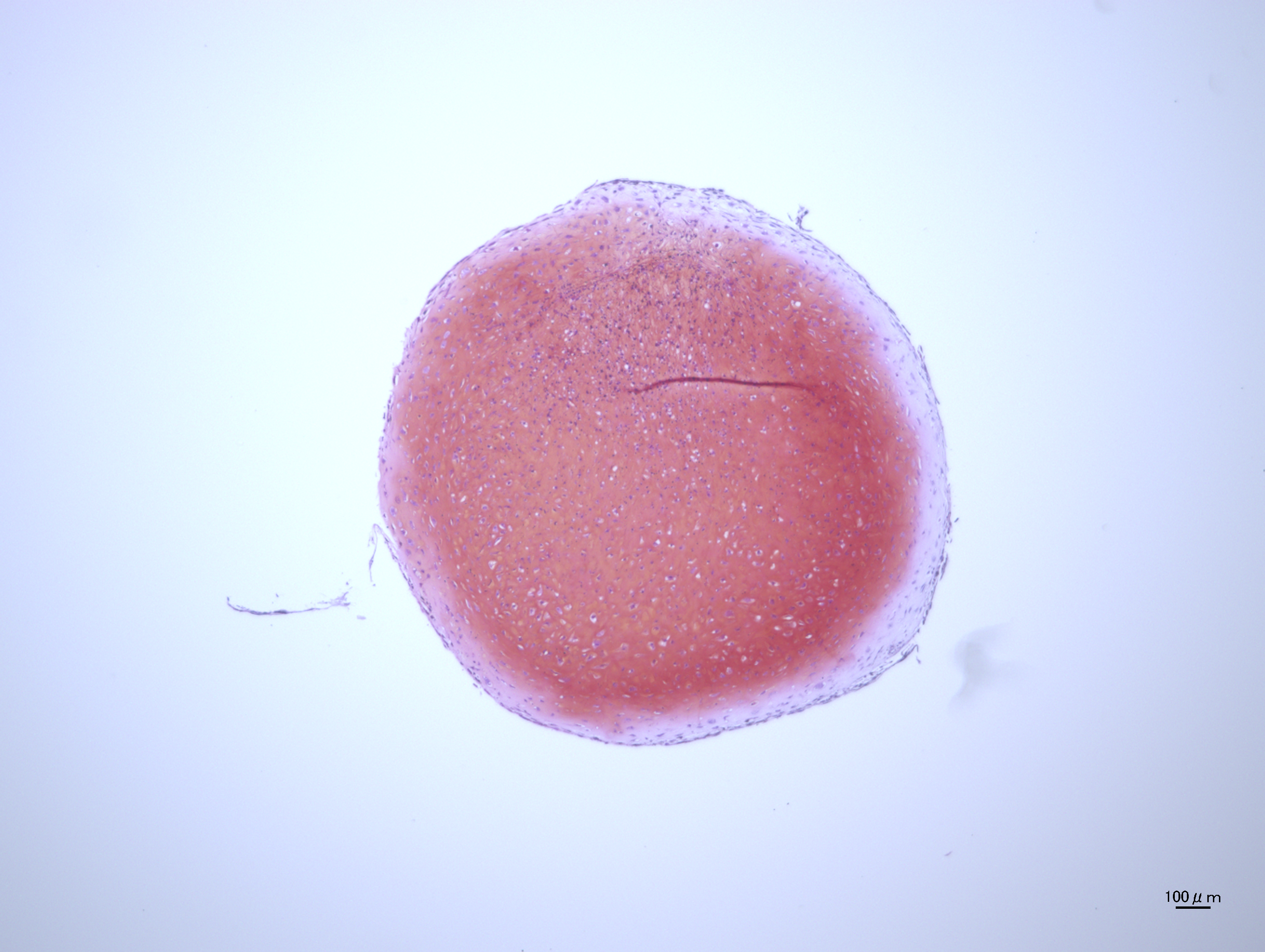

Supplement: S3 File — (ZIP) [file pone.0279584.s017.zip › S3 files/IHC/S-O/Mock.tif]

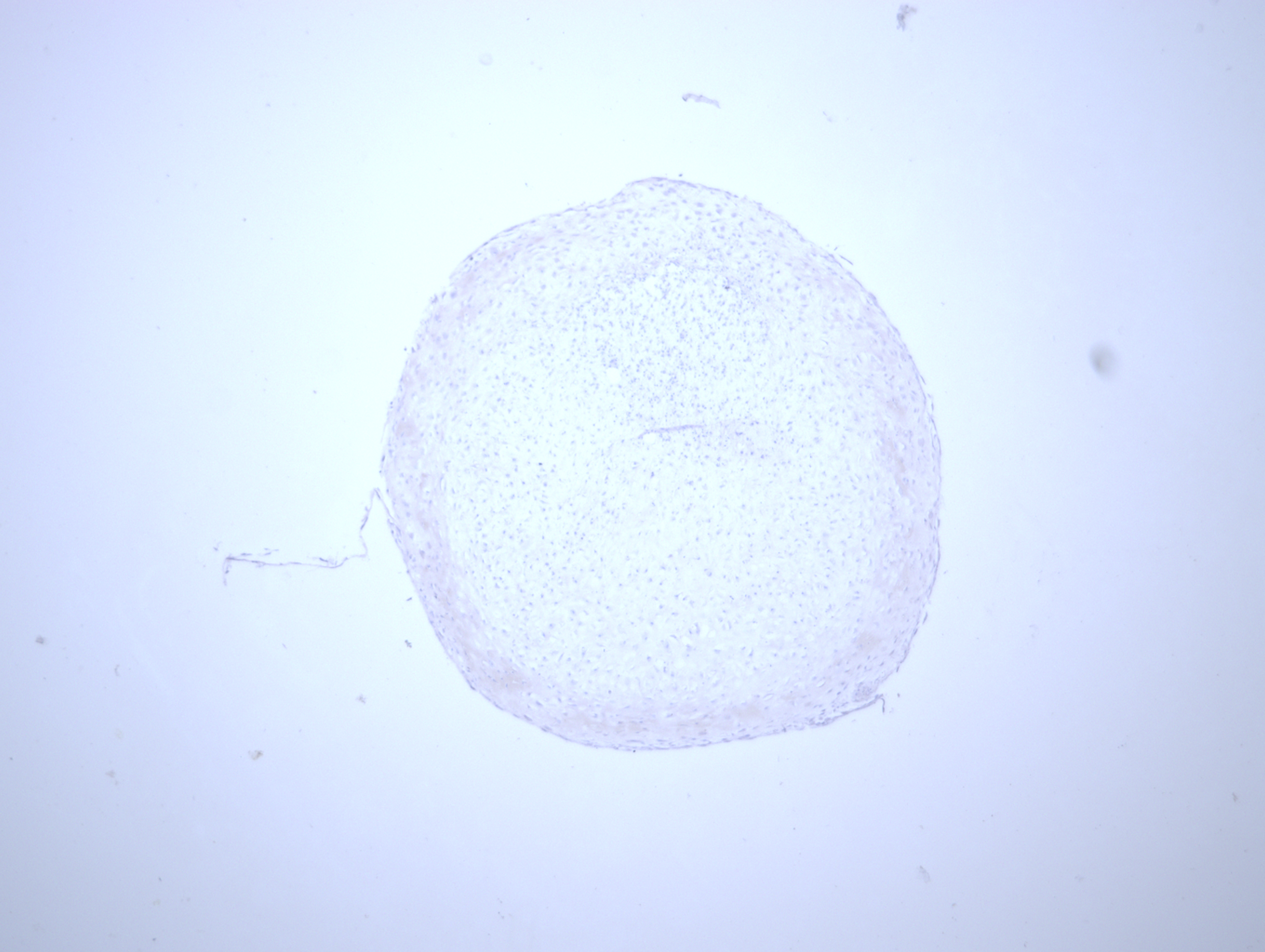

Supplement: S3 File — (ZIP) [file pone.0279584.s017.zip › S3 files/IHC/COL2/Mock IgG.tif]

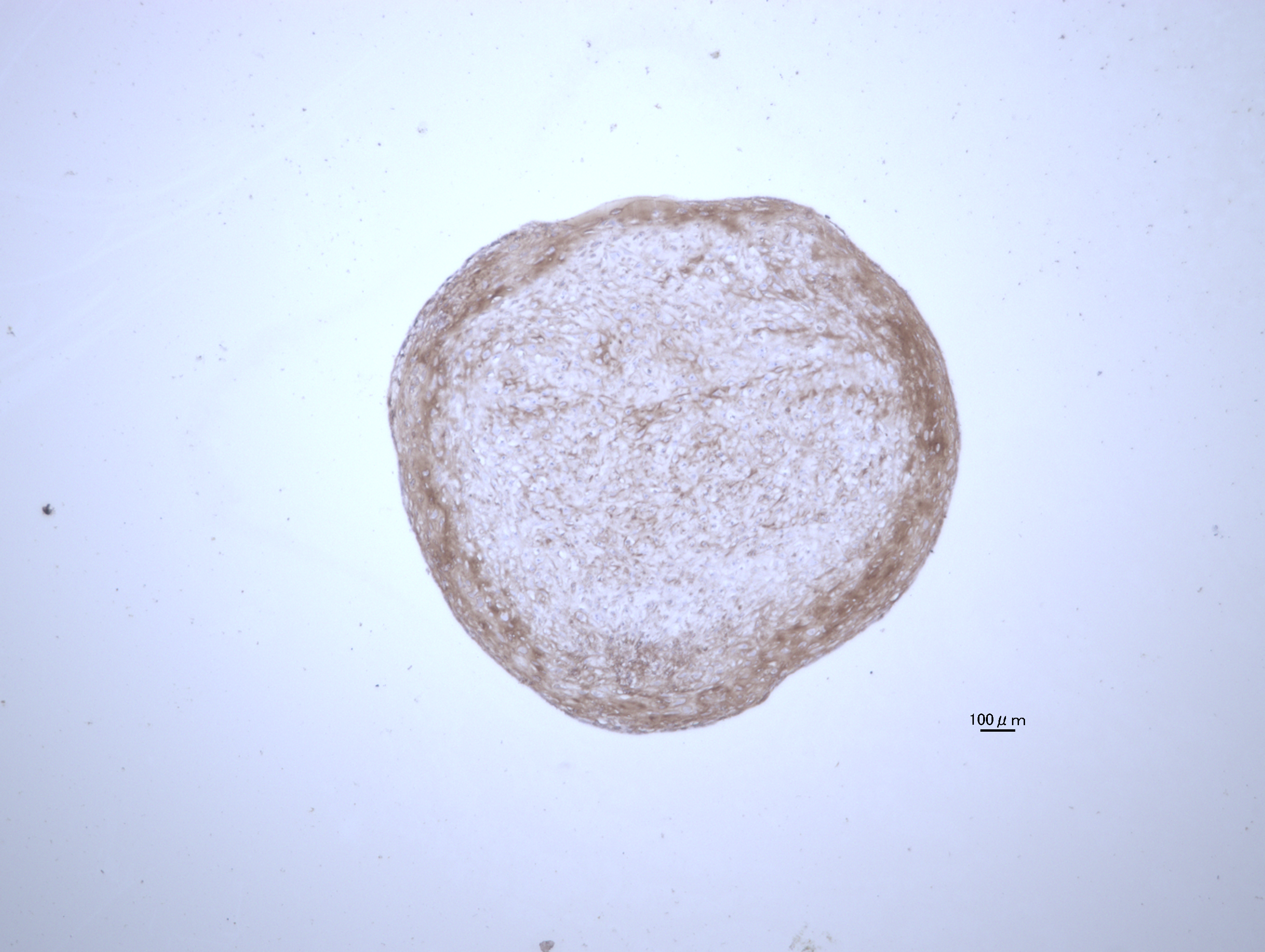

Supplement: S3 File — (ZIP) [file pone.0279584.s017.zip › S3 files/IHC/COL2/pEF6-EBI3-V5.tif]

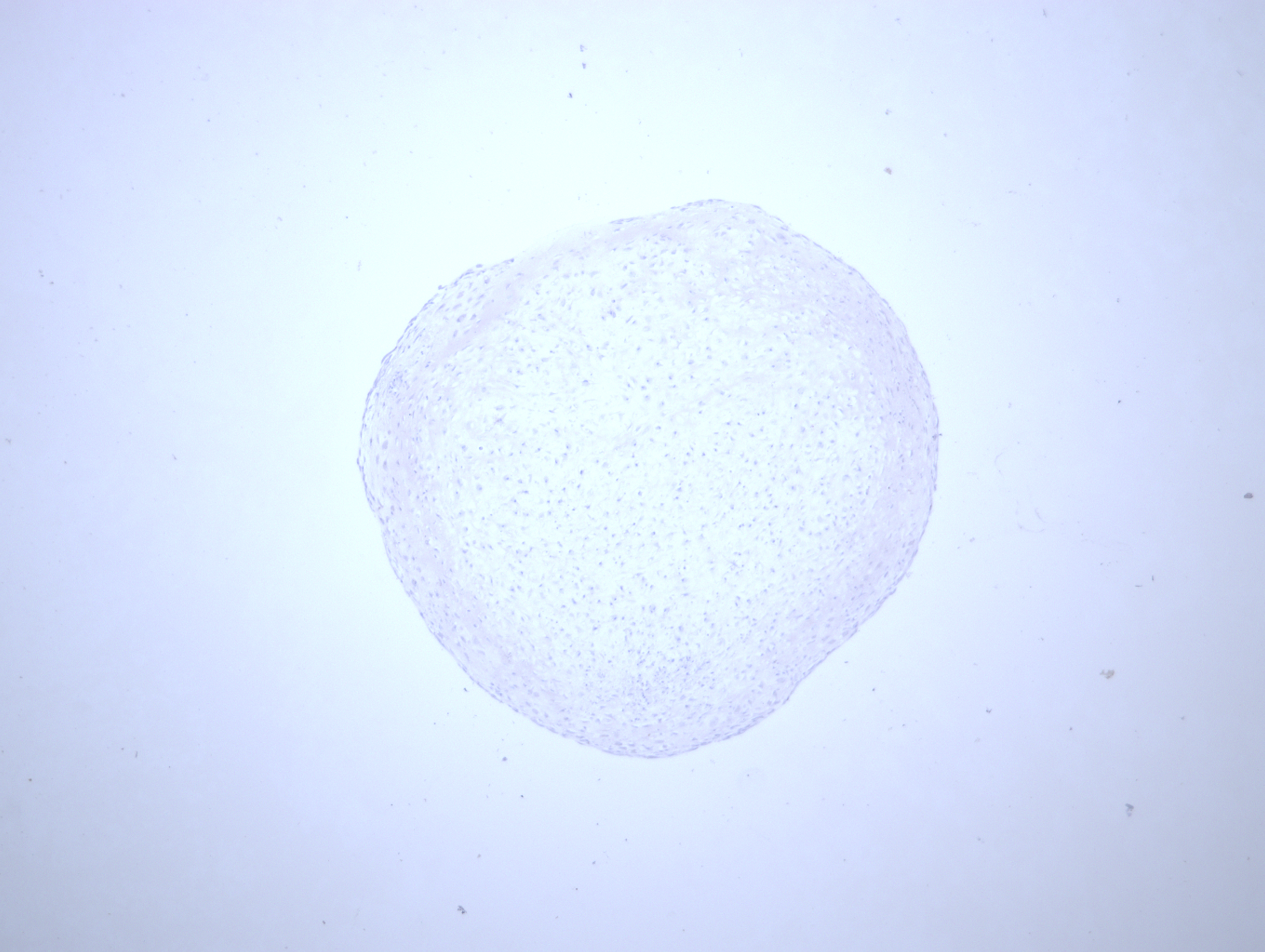

Supplement: S3 File — (ZIP) [file pone.0279584.s017.zip › S3 files/IHC/COL2/pEF6-EBI3-V5 IgG.tif]

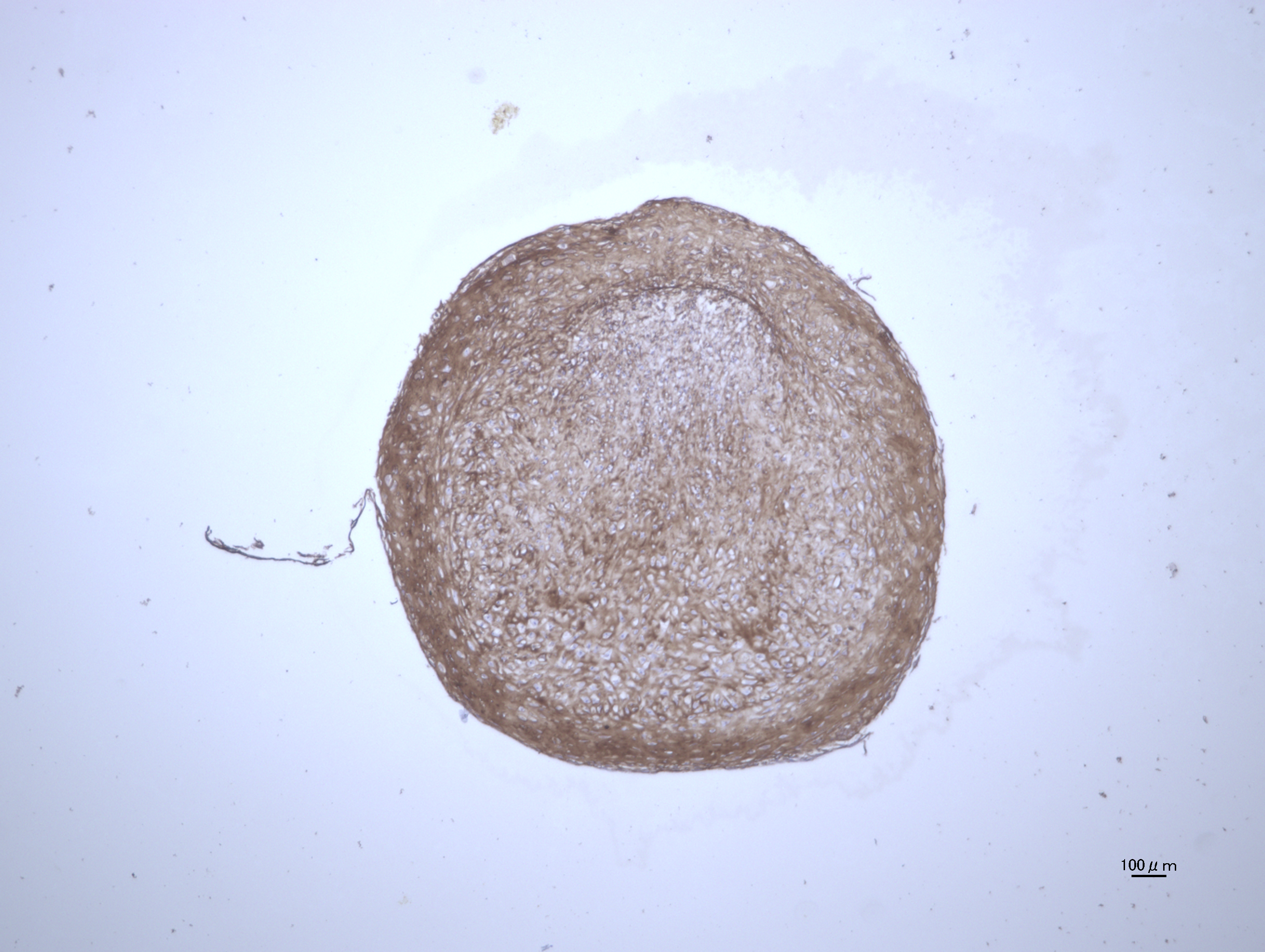

Supplement: S3 File — (ZIP) [file pone.0279584.s017.zip › S3 files/IHC/COL2/Mock.tif]

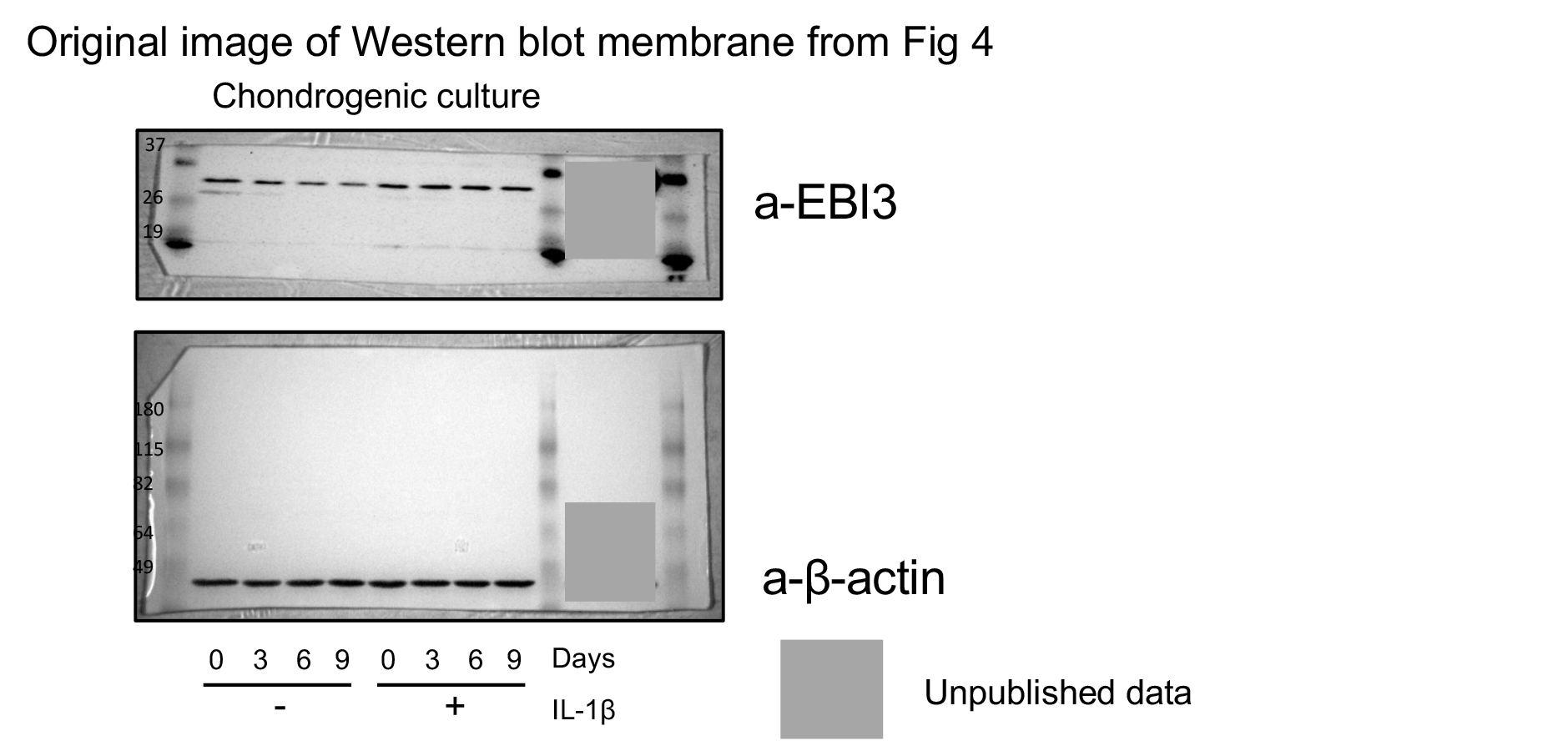

Supplement: S4 File — (ZIP) [file pone.0279584.s018.zip › S4 files/WB Fig 4.tif]

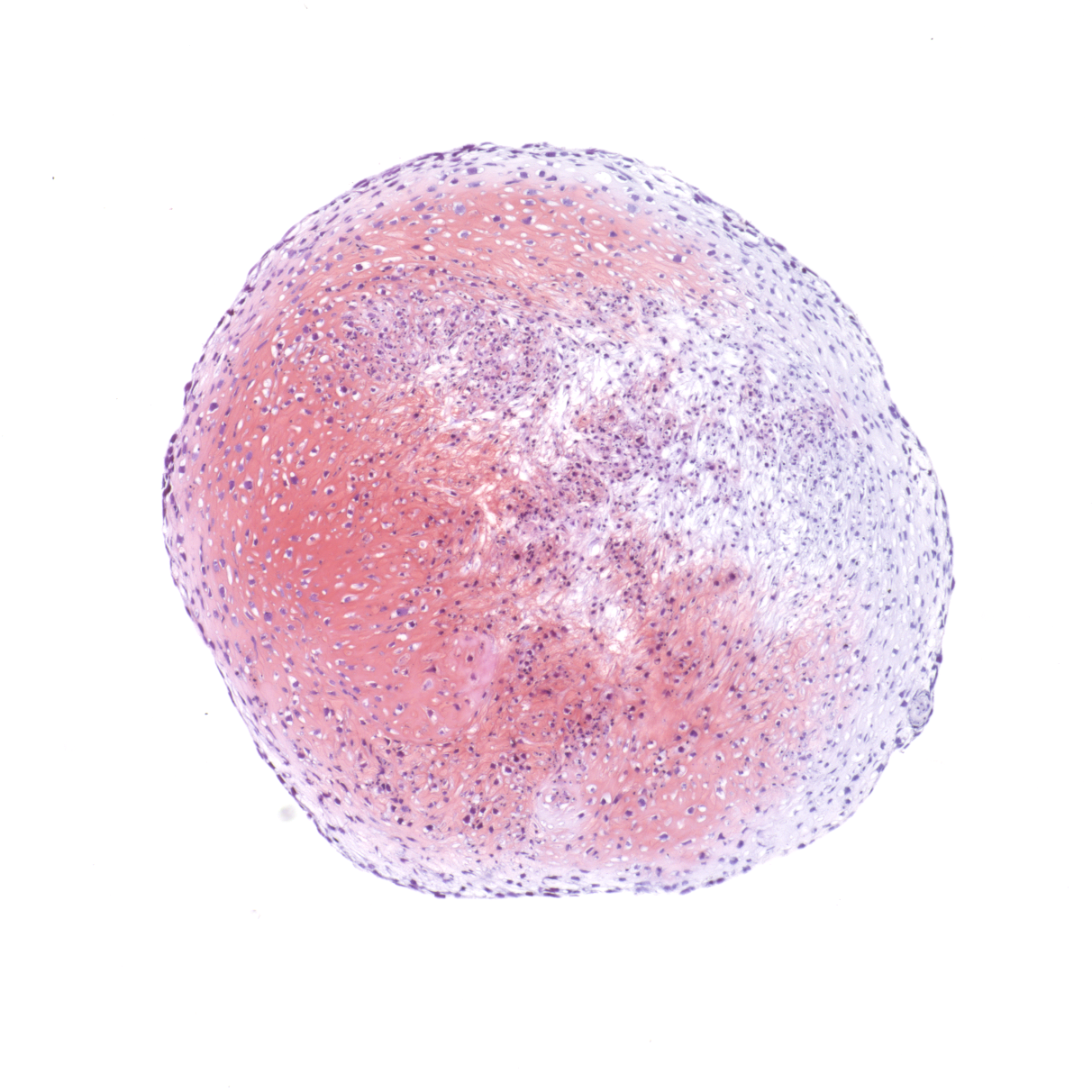

Supplement: S4 File — (ZIP) [file pone.0279584.s018.zip › S4 files/IHC/S-O/IL6 and R.tif]

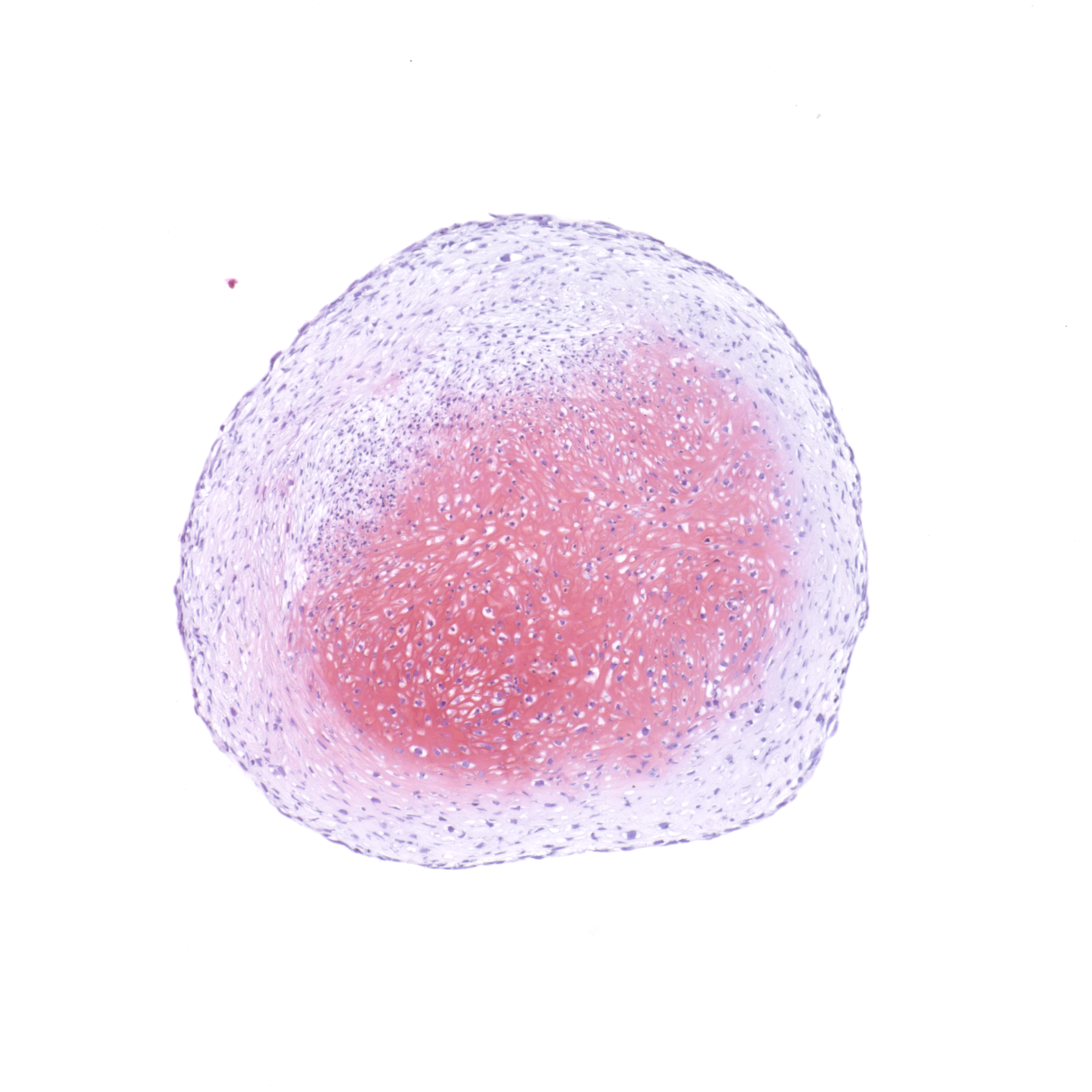

Supplement: S4 File — (ZIP) [file pone.0279584.s018.zip › S4 files/IHC/S-O/NS.tif]

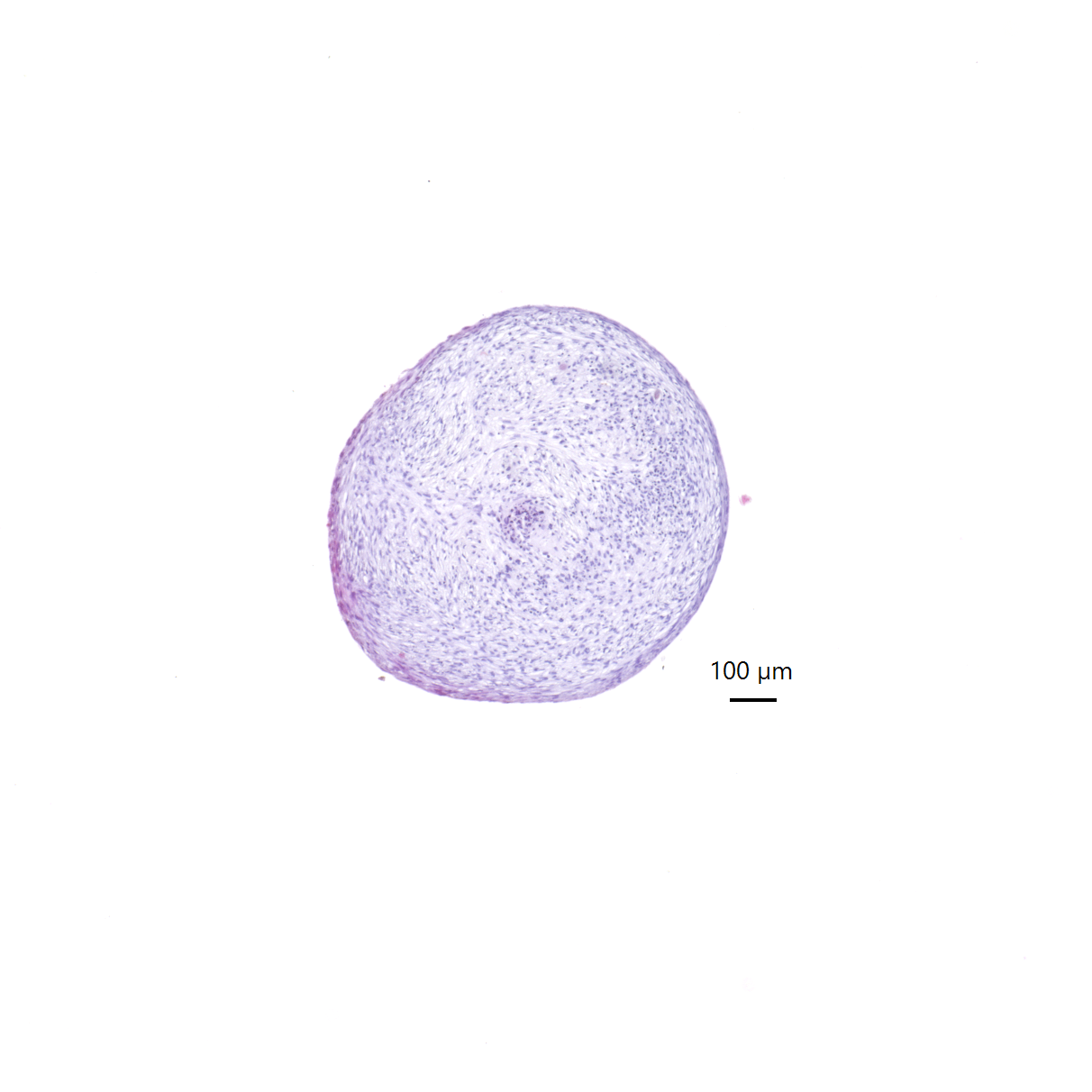

Supplement: S4 File — (ZIP) [file pone.0279584.s018.zip › S4 files/IHC/S-O/IL-1b.tif]

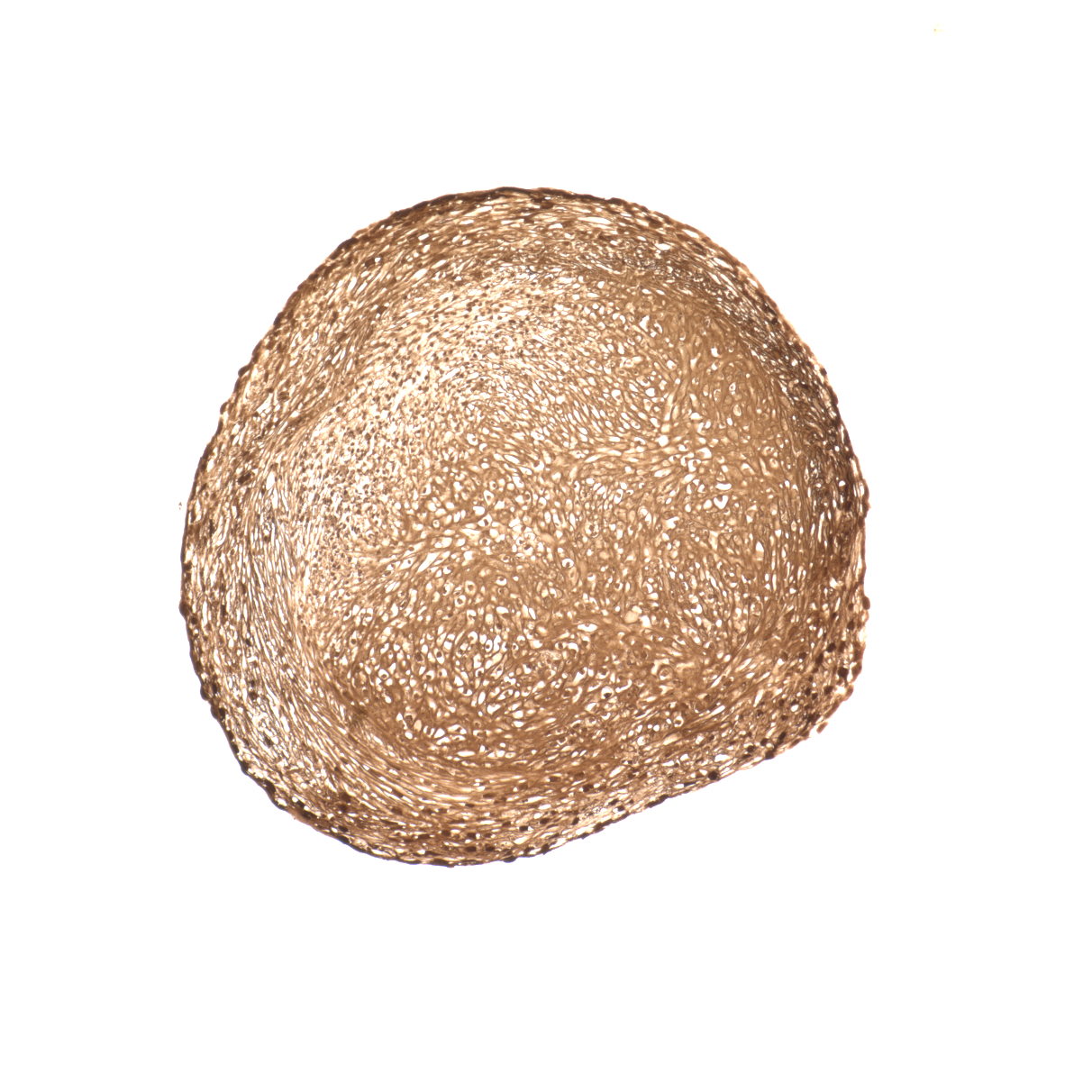

Supplement: S4 File — (ZIP) [file pone.0279584.s018.zip › S4 files/IHC/COL2/NS.tif]

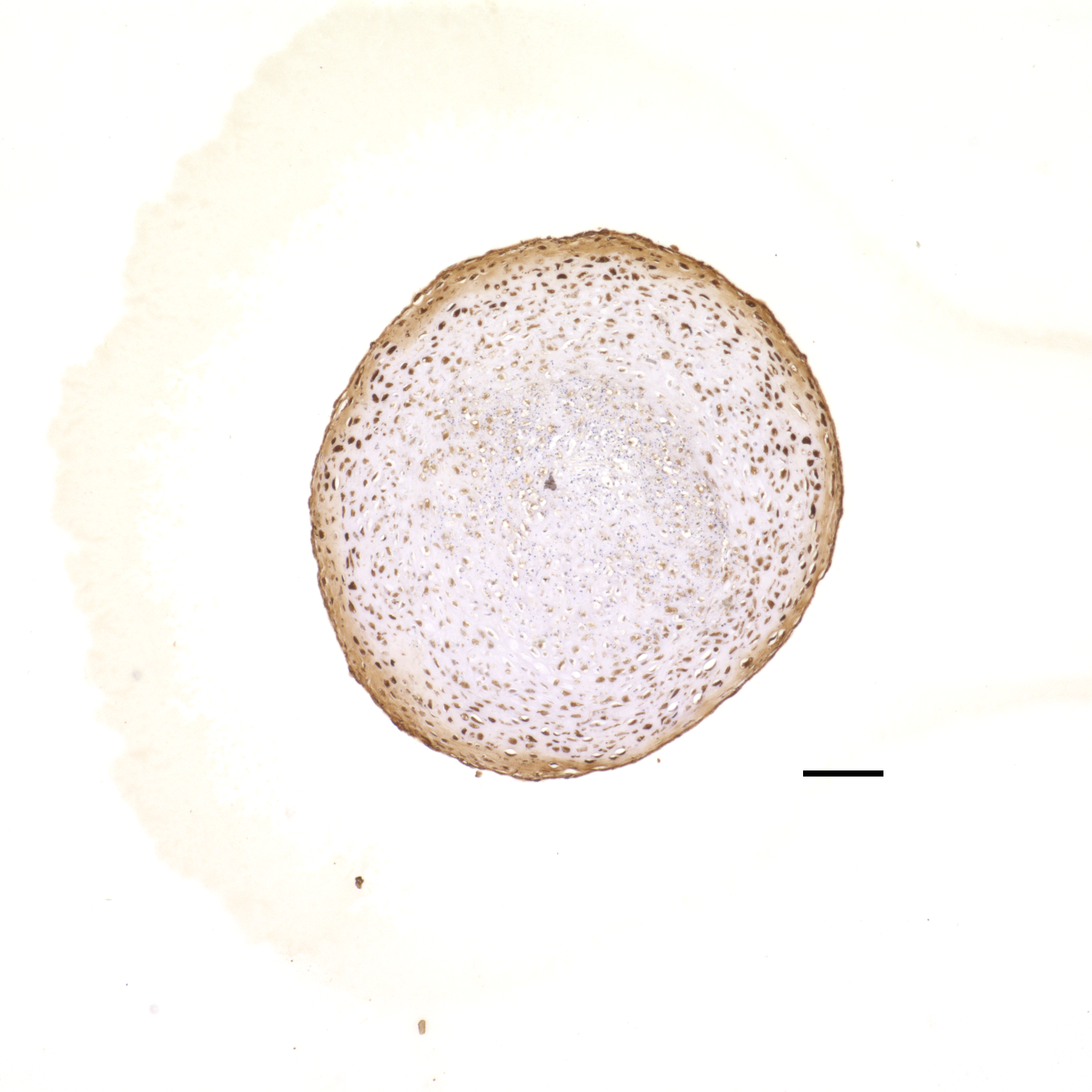

Supplement: S4 File — (ZIP) [file pone.0279584.s018.zip › S4 files/IHC/COL2/IL-1b.tif]

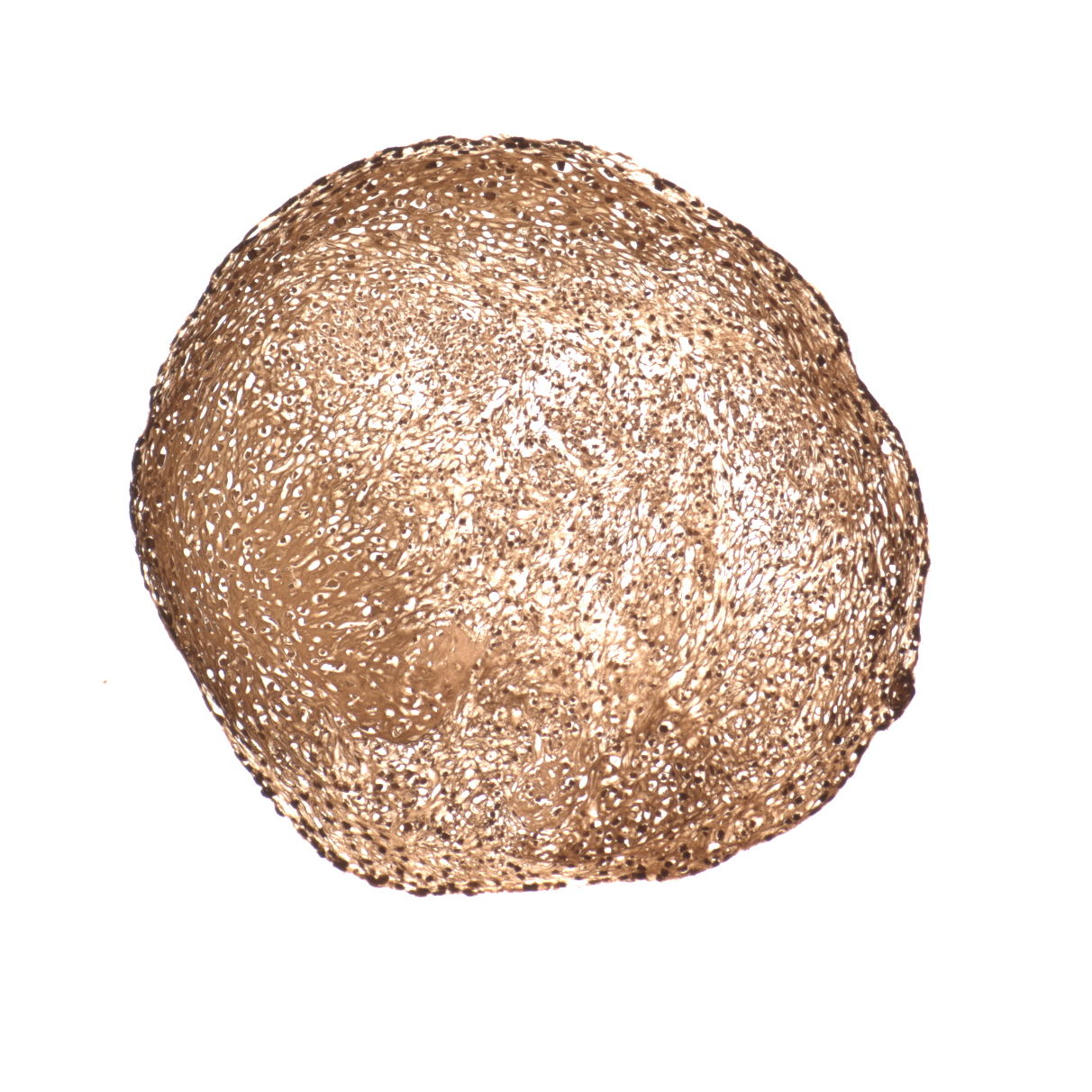

Supplement: S4 File — (ZIP) [file pone.0279584.s018.zip › S4 files/IHC/COL2/IL-6 and R.tif]

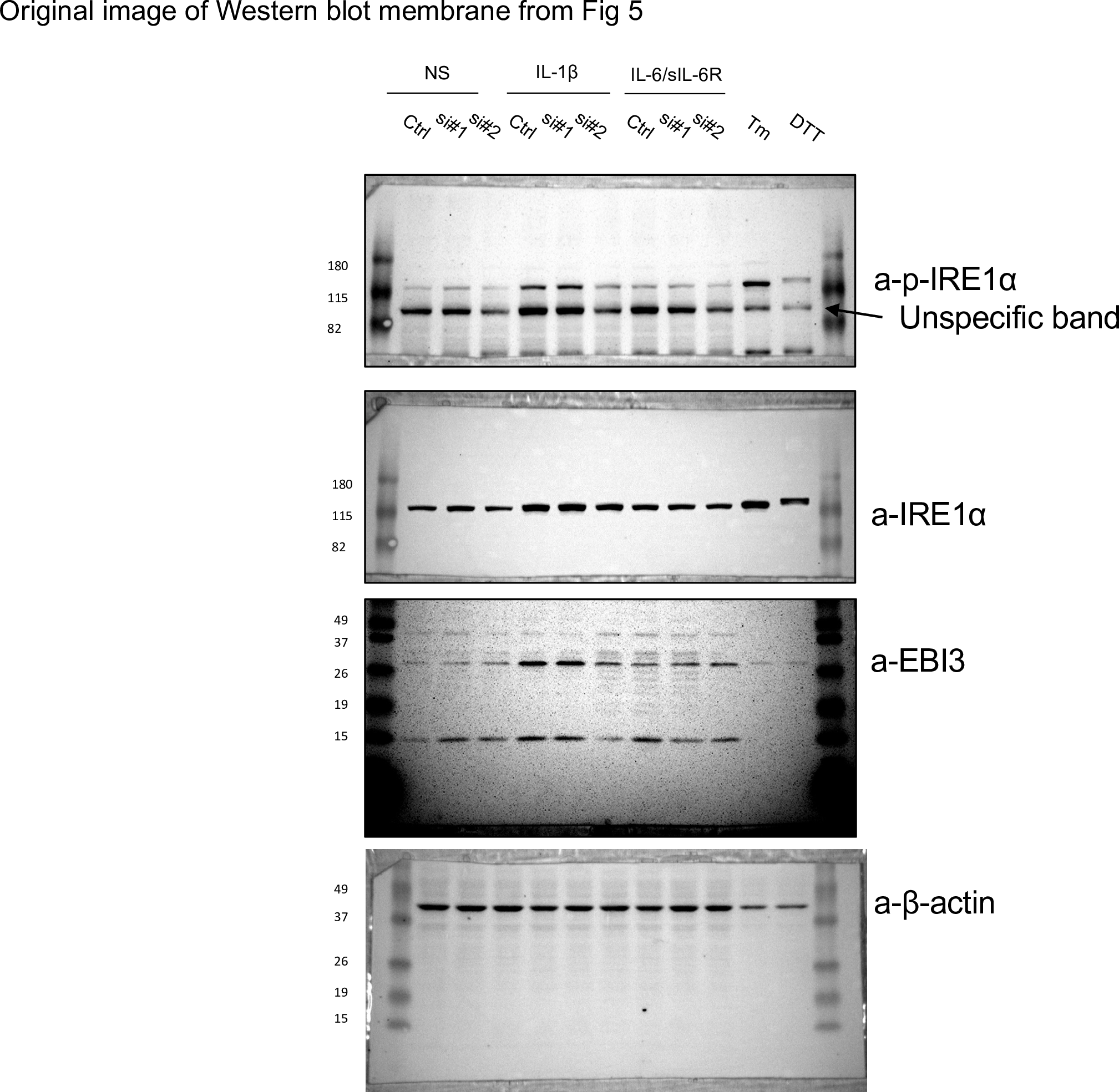

Supplement: S5 File — (ZIP) [file pone.0279584.s019.zip › S5_C files/WB Fig 5.tif]

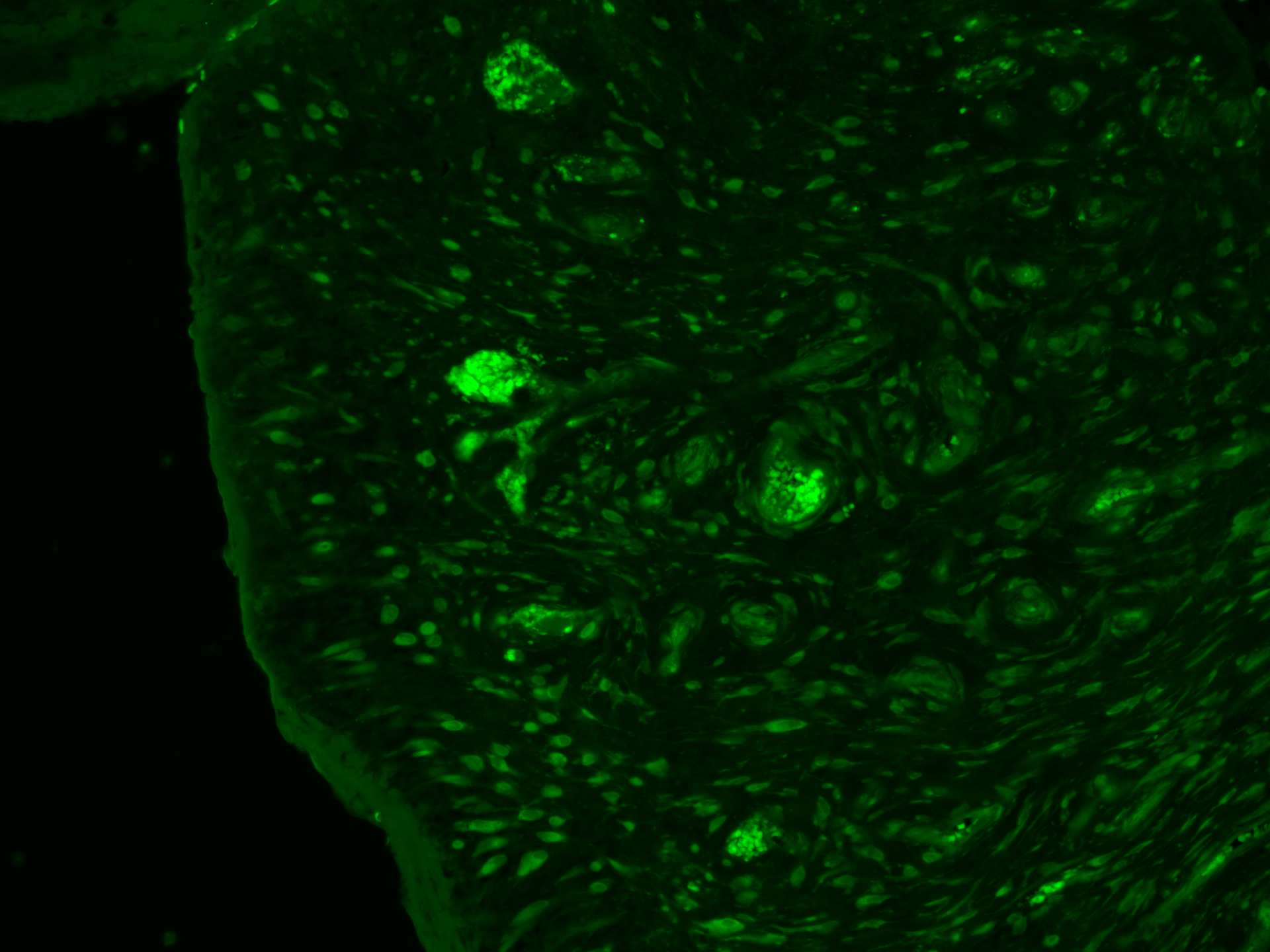

Supplement: S5 File — (ZIP) [file pone.0279584.s019.zip › S5_C files/IF/CD271 EBI3 IRE1/RA/Alexa Fluor 488_IRE1.tif]

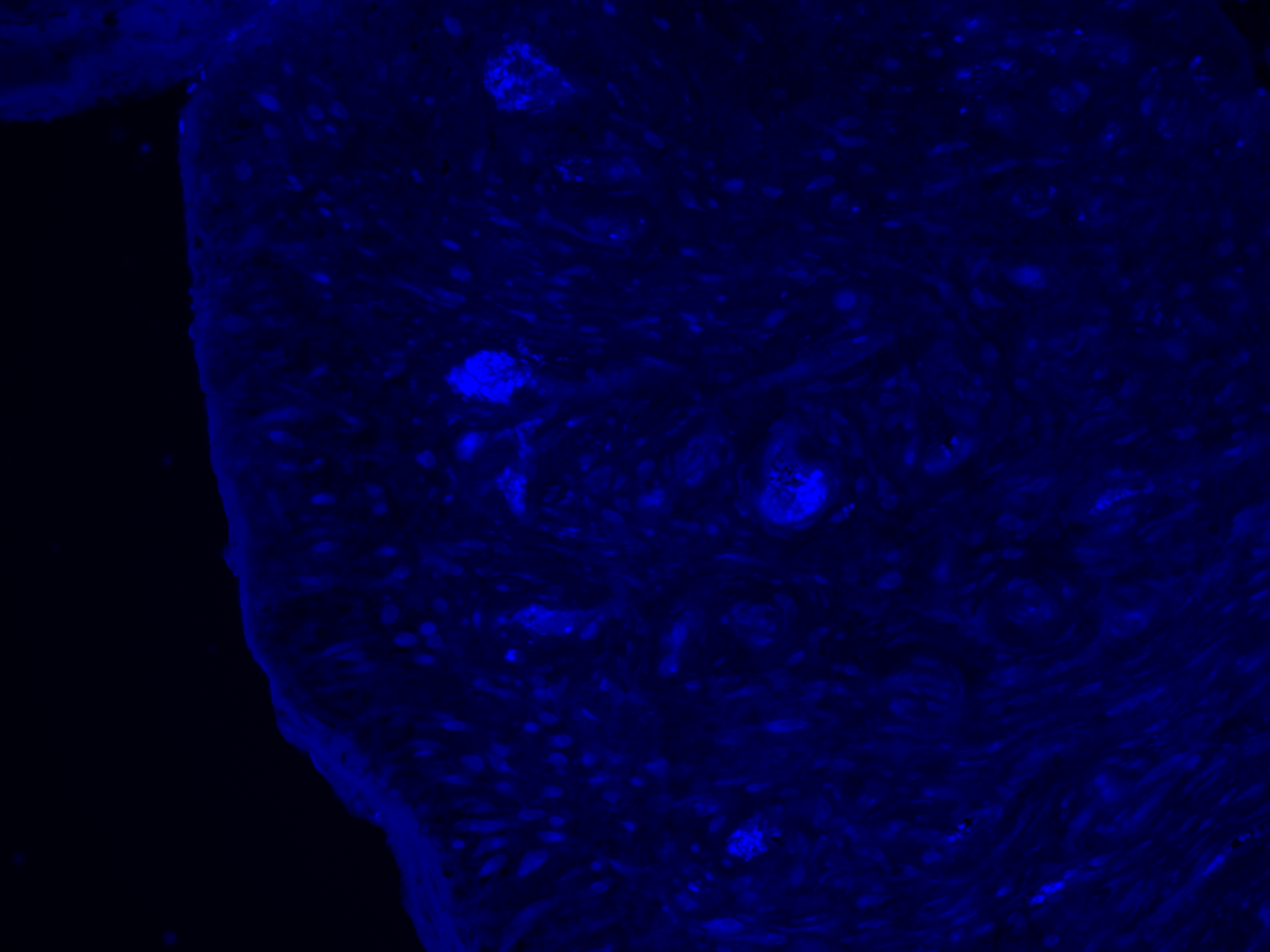

Supplement: S5 File — (ZIP) [file pone.0279584.s019.zip › S5_C files/IF/CD271 EBI3 IRE1/RA/Alexa Fluor 350_CD271.tif]

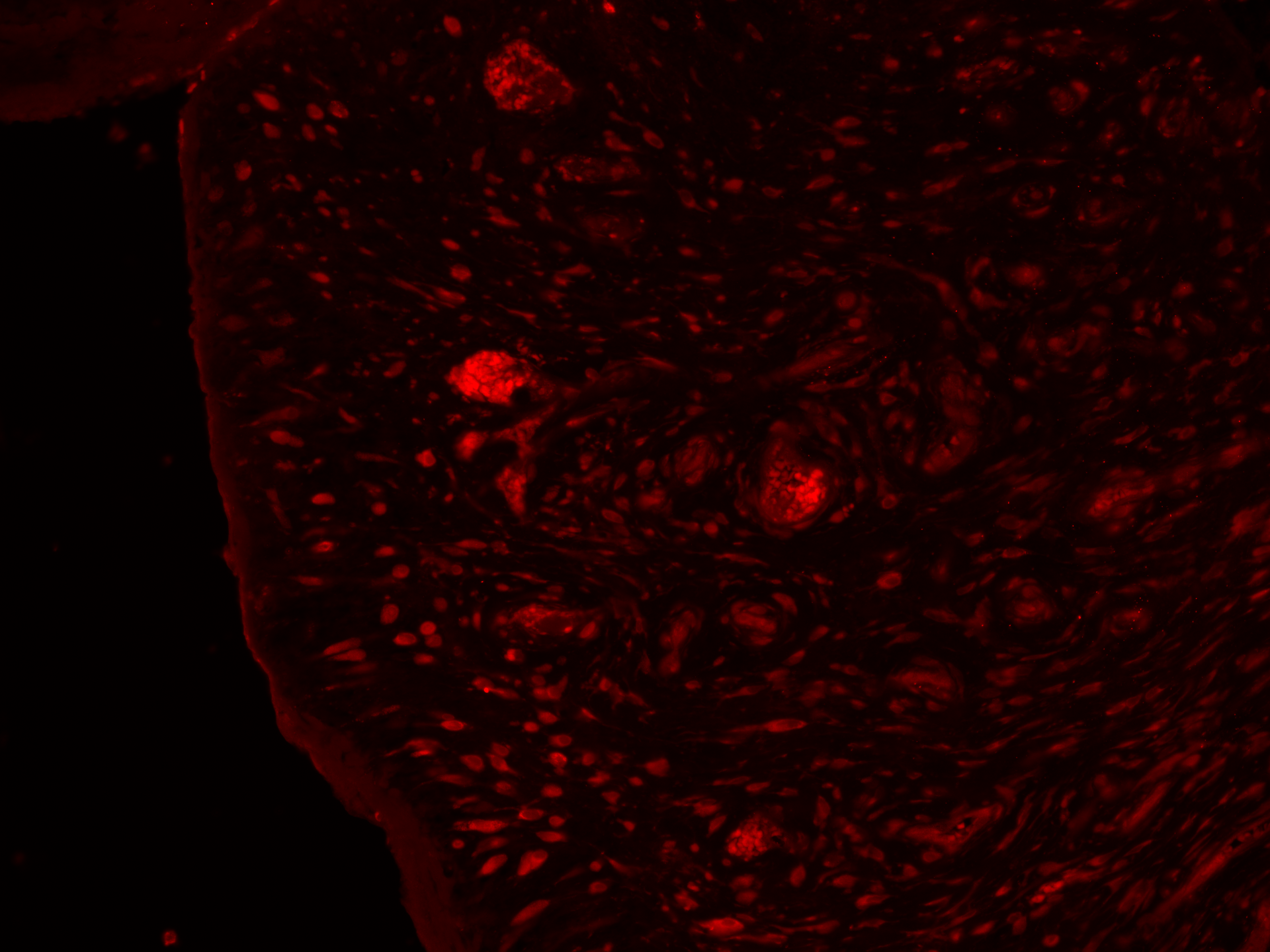

Supplement: S5 File — (ZIP) [file pone.0279584.s019.zip › S5_C files/IF/CD271 EBI3 IRE1/RA/Alexa Fluor 568_EBI3.tif]

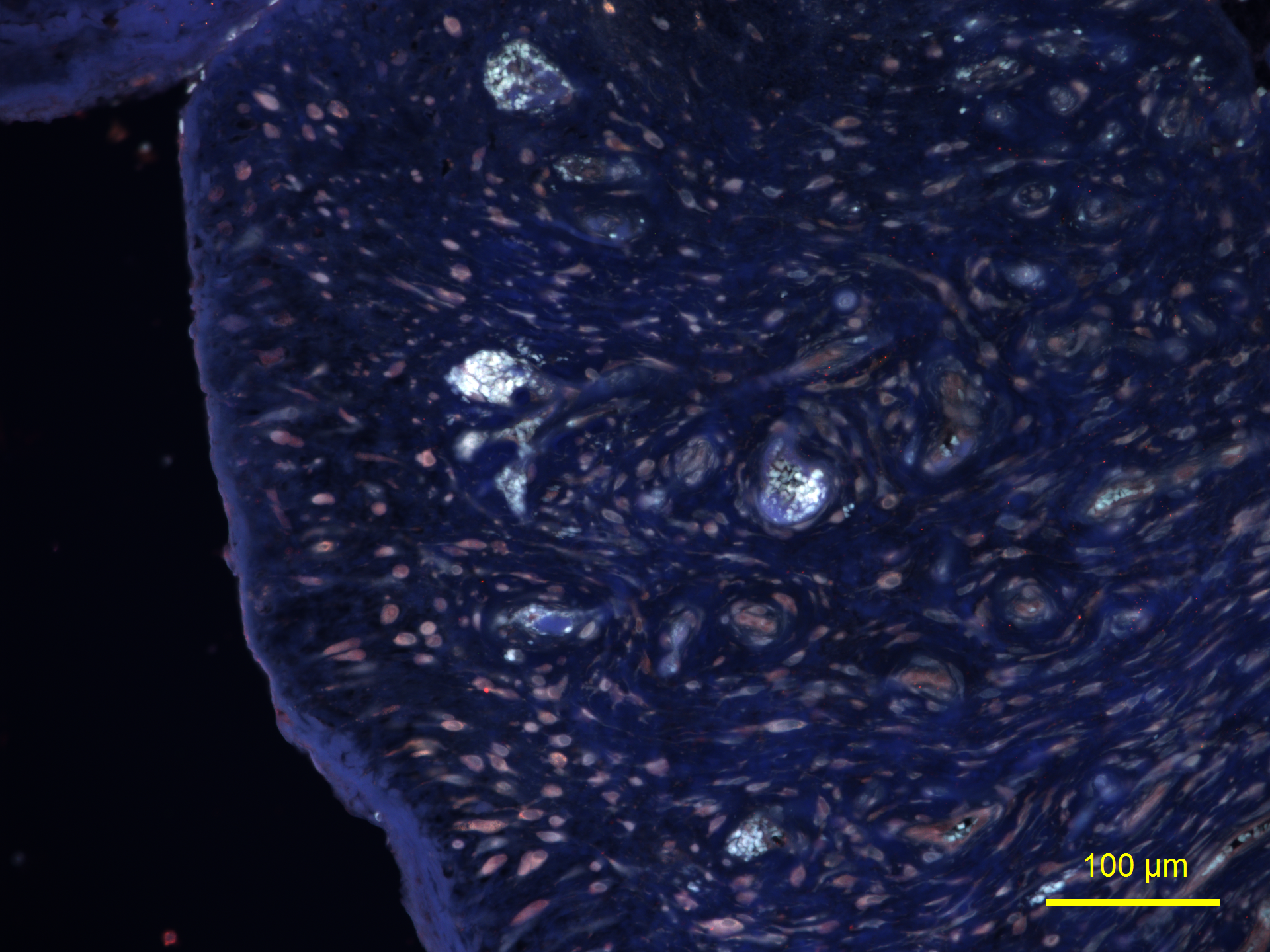

Supplement: S5 File — (ZIP) [file pone.0279584.s019.zip › S5_C files/IF/CD271 EBI3 IRE1/RA/Image_Overlay bar.tif]

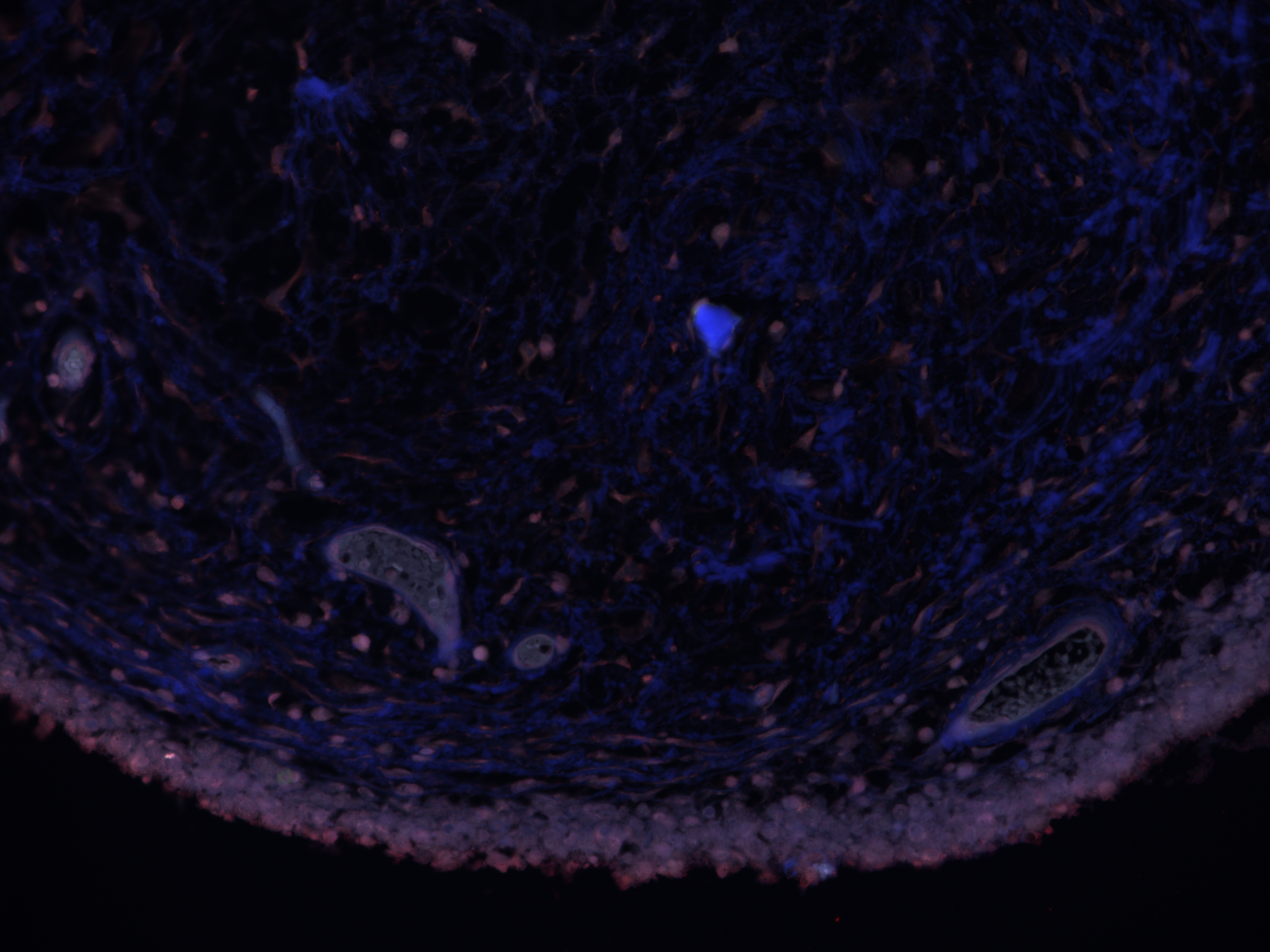

Supplement: S5 File — (ZIP) [file pone.0279584.s019.zip › S5_C files/IF/CD271 EBI3 IRE1/OA/Overlay_Image_overlay.tif]

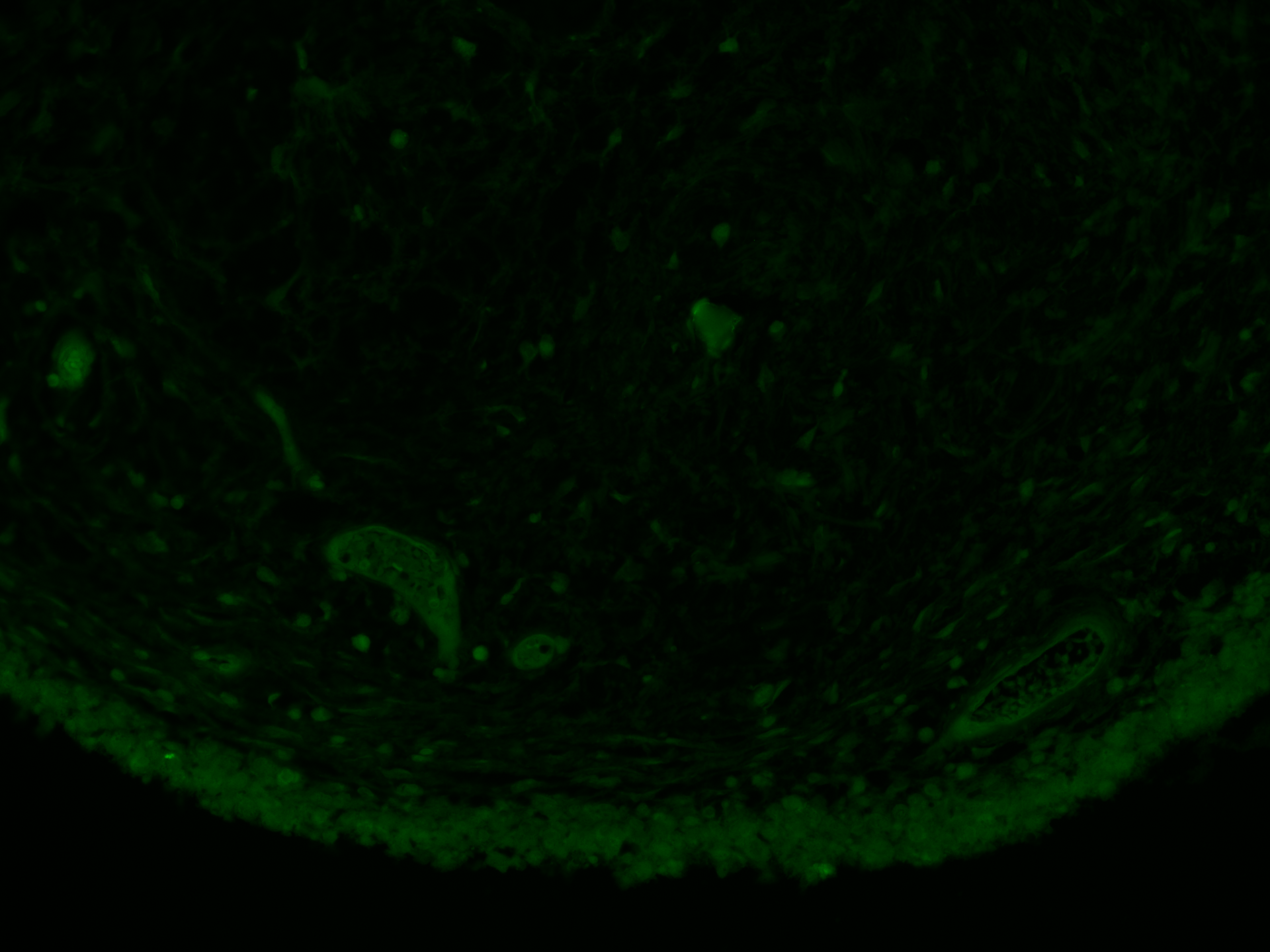

Supplement: S5 File — (ZIP) [file pone.0279584.s019.zip › S5_C files/IF/CD271 EBI3 IRE1/OA/Alexa Fluor 488_IRE1.tif]

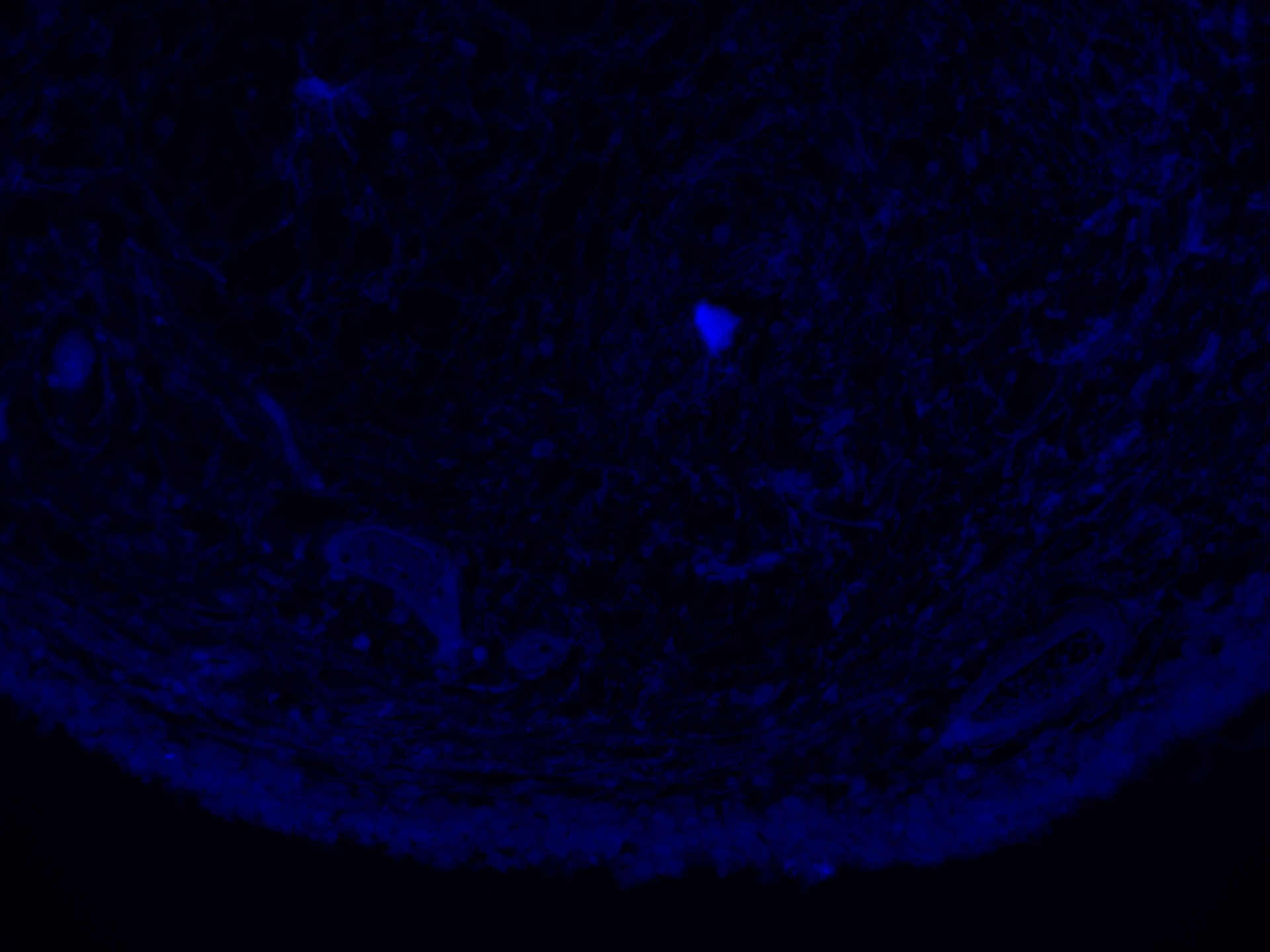

Supplement: S5 File — (ZIP) [file pone.0279584.s019.zip › S5_C files/IF/CD271 EBI3 IRE1/OA/Alexa Fluor 350_CD271.tif]

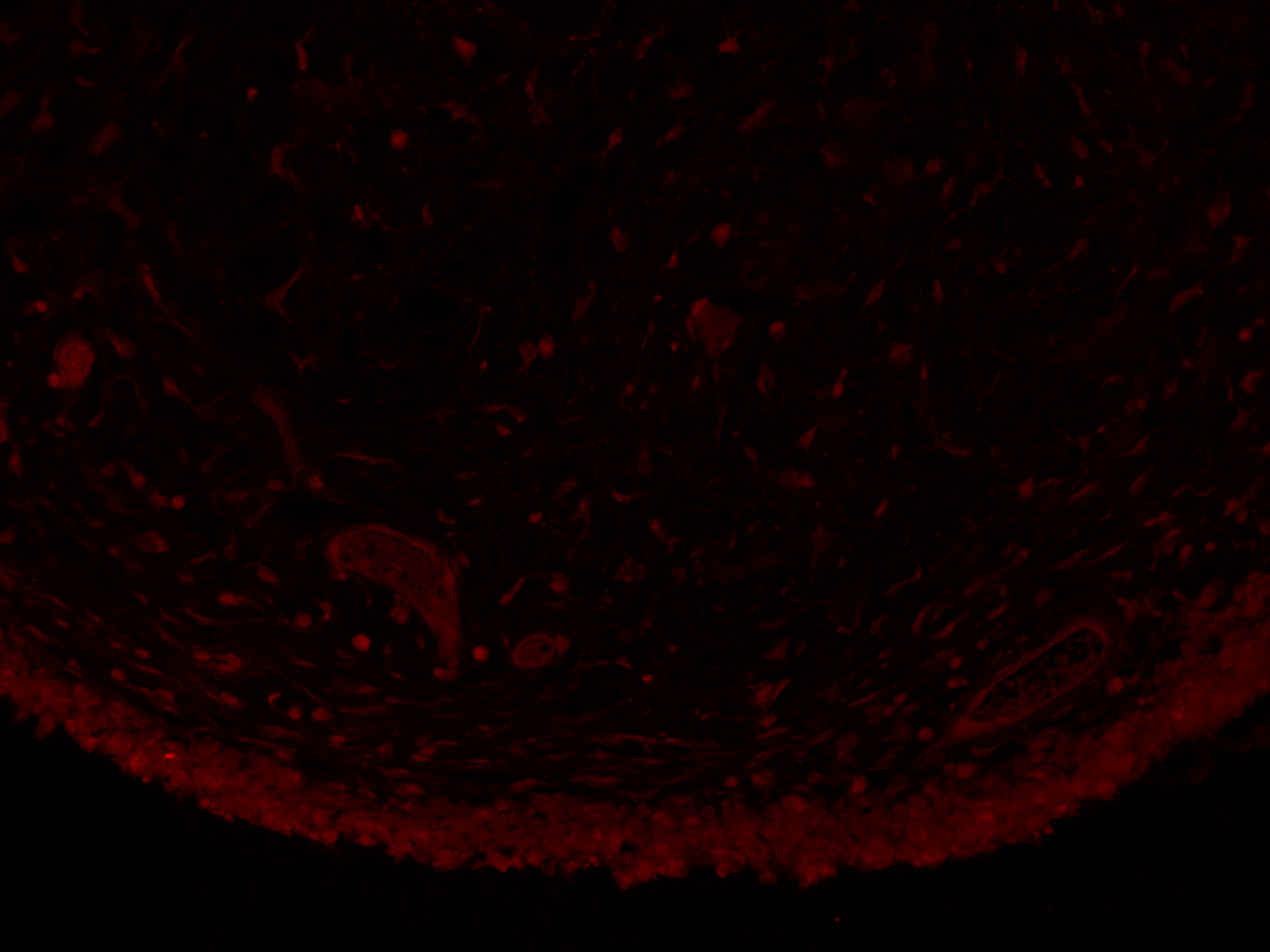

Supplement: S5 File — (ZIP) [file pone.0279584.s019.zip › S5_C files/IF/CD271 EBI3 IRE1/OA/Alexa Fluor 568_EBI3.tif]

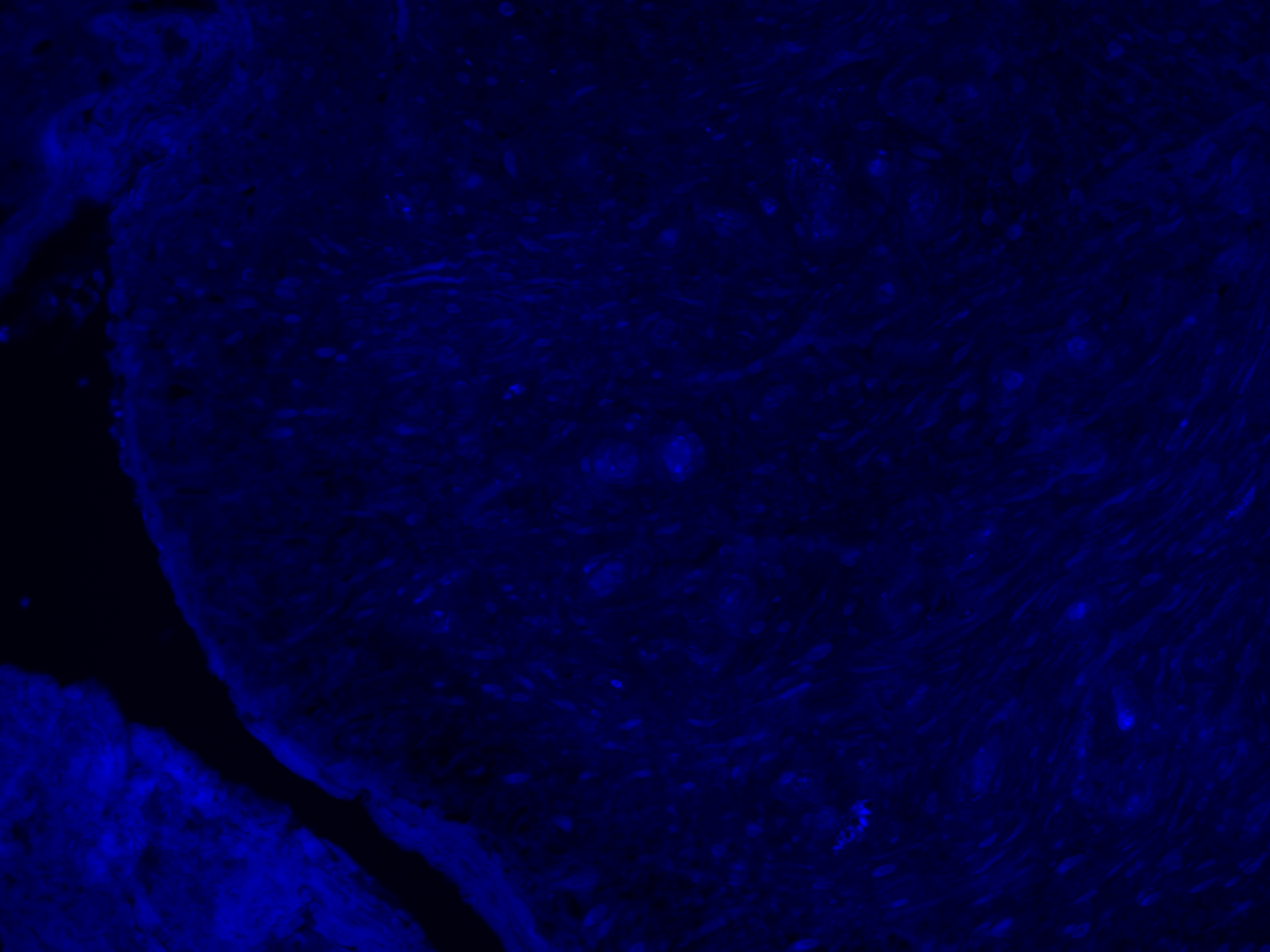

Supplement: S5 File — (ZIP) [file pone.0279584.s019.zip › S5_C files/IF/CD271 EBI3 IRE1/RA/IgG/Image_CH3.tif]

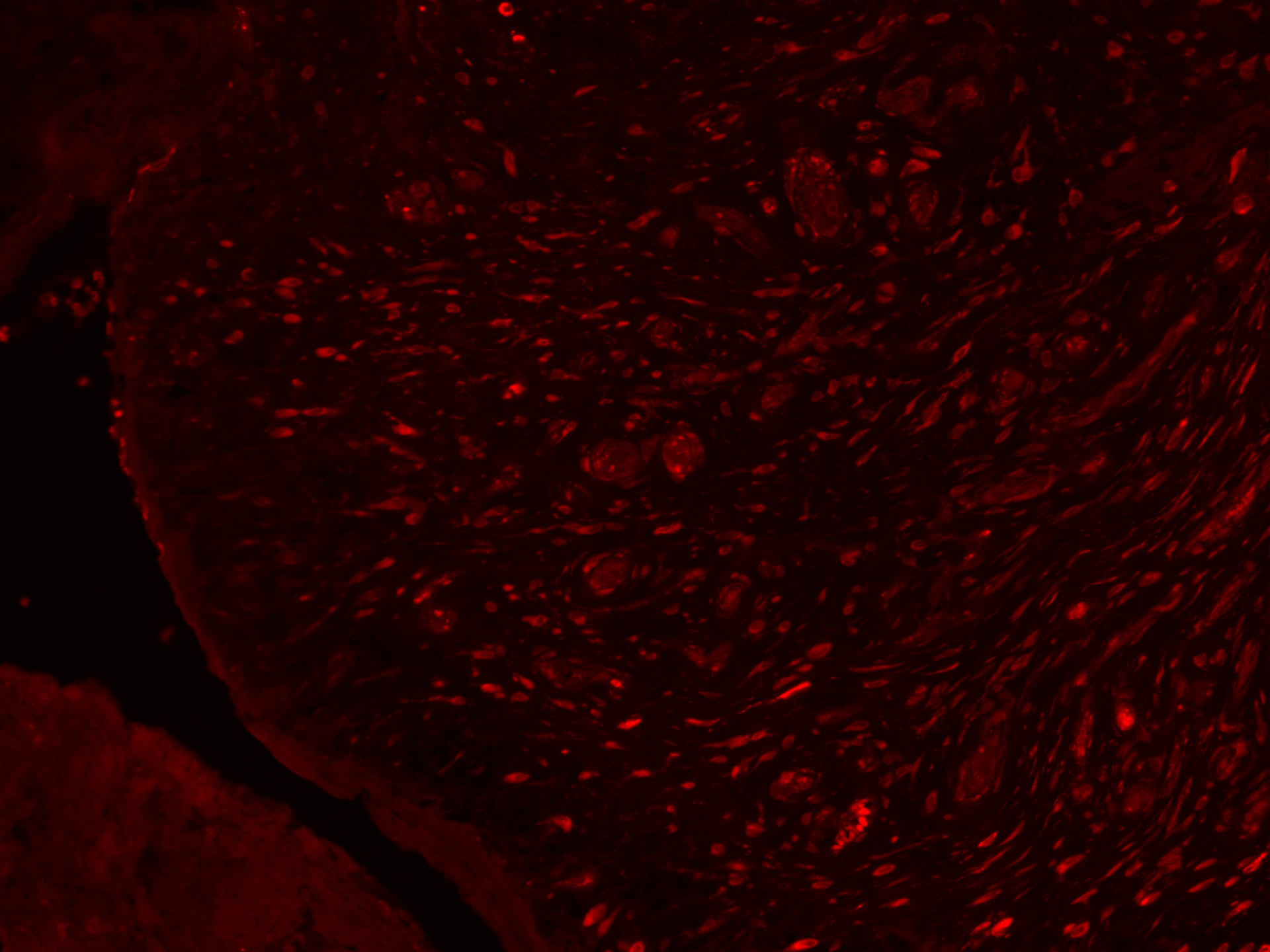

Supplement: S5 File — (ZIP) [file pone.0279584.s019.zip › S5_C files/IF/CD271 EBI3 IRE1/RA/IgG/Image_CH2.tif]

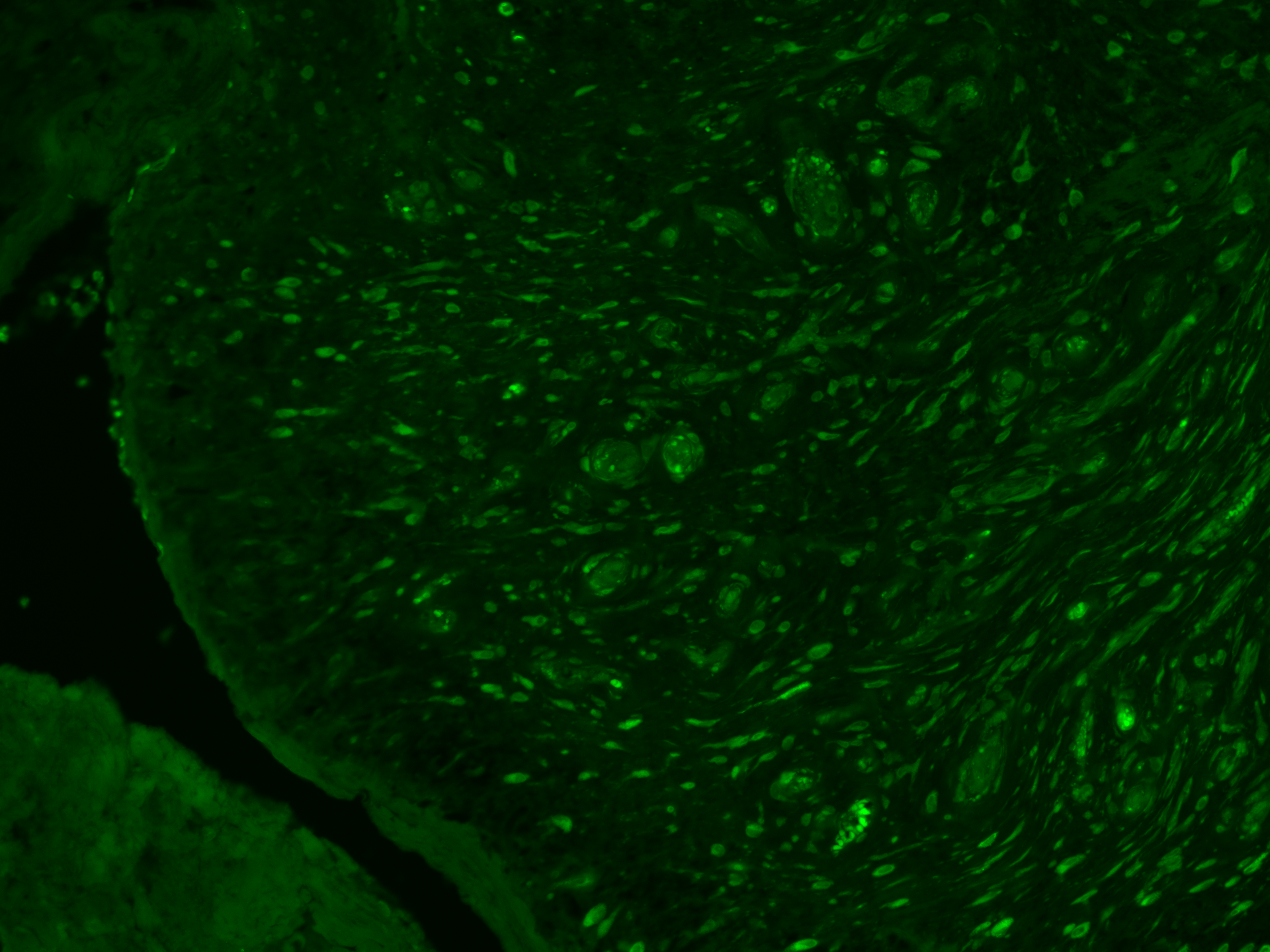

Supplement: S5 File — (ZIP) [file pone.0279584.s019.zip › S5_C files/IF/CD271 EBI3 IRE1/RA/IgG/Image_CH1.tif]

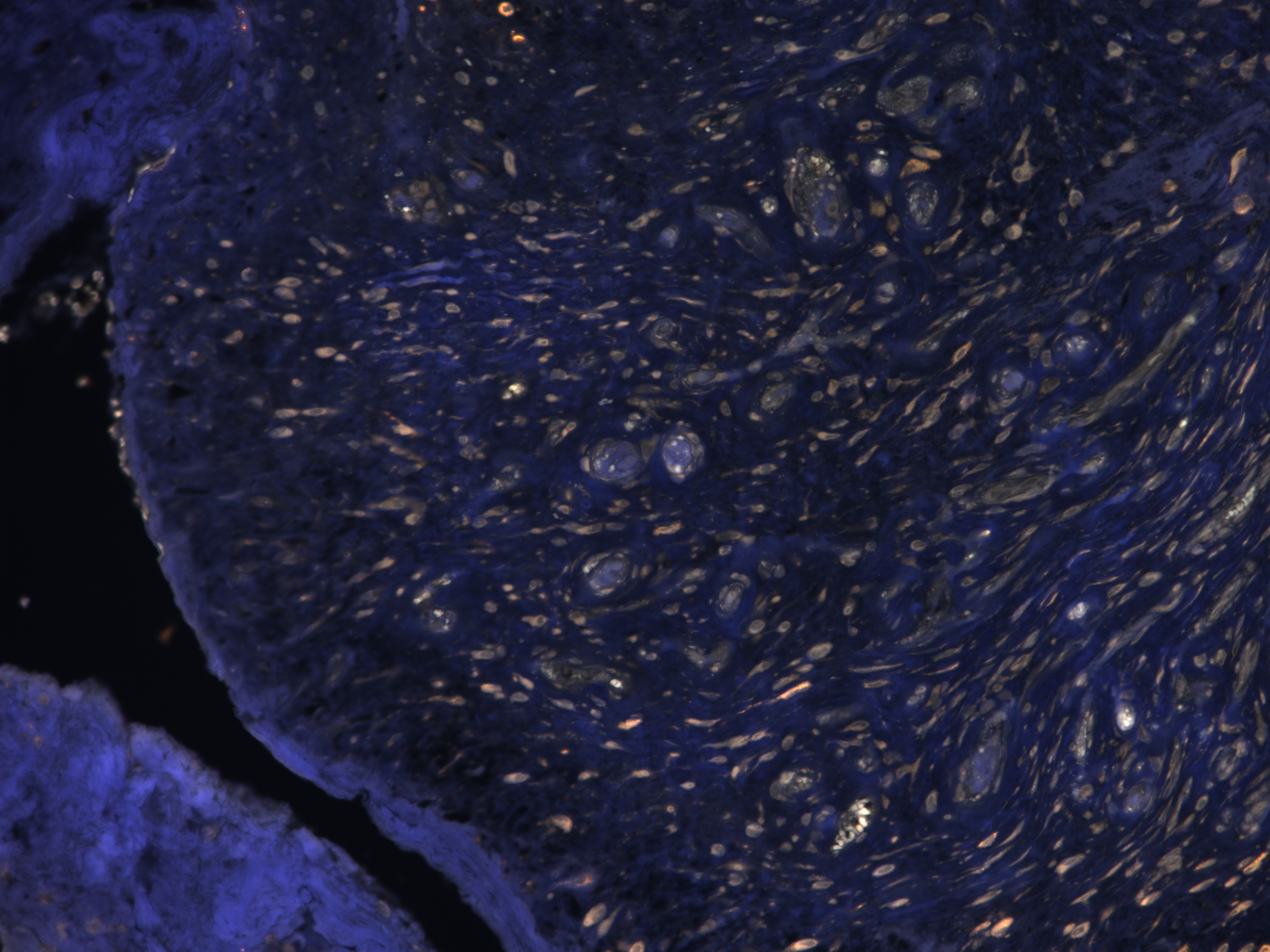

Supplement: S5 File — (ZIP) [file pone.0279584.s019.zip › S5_C files/IF/CD271 EBI3 IRE1/RA/IgG/Image_Overlay.tif]

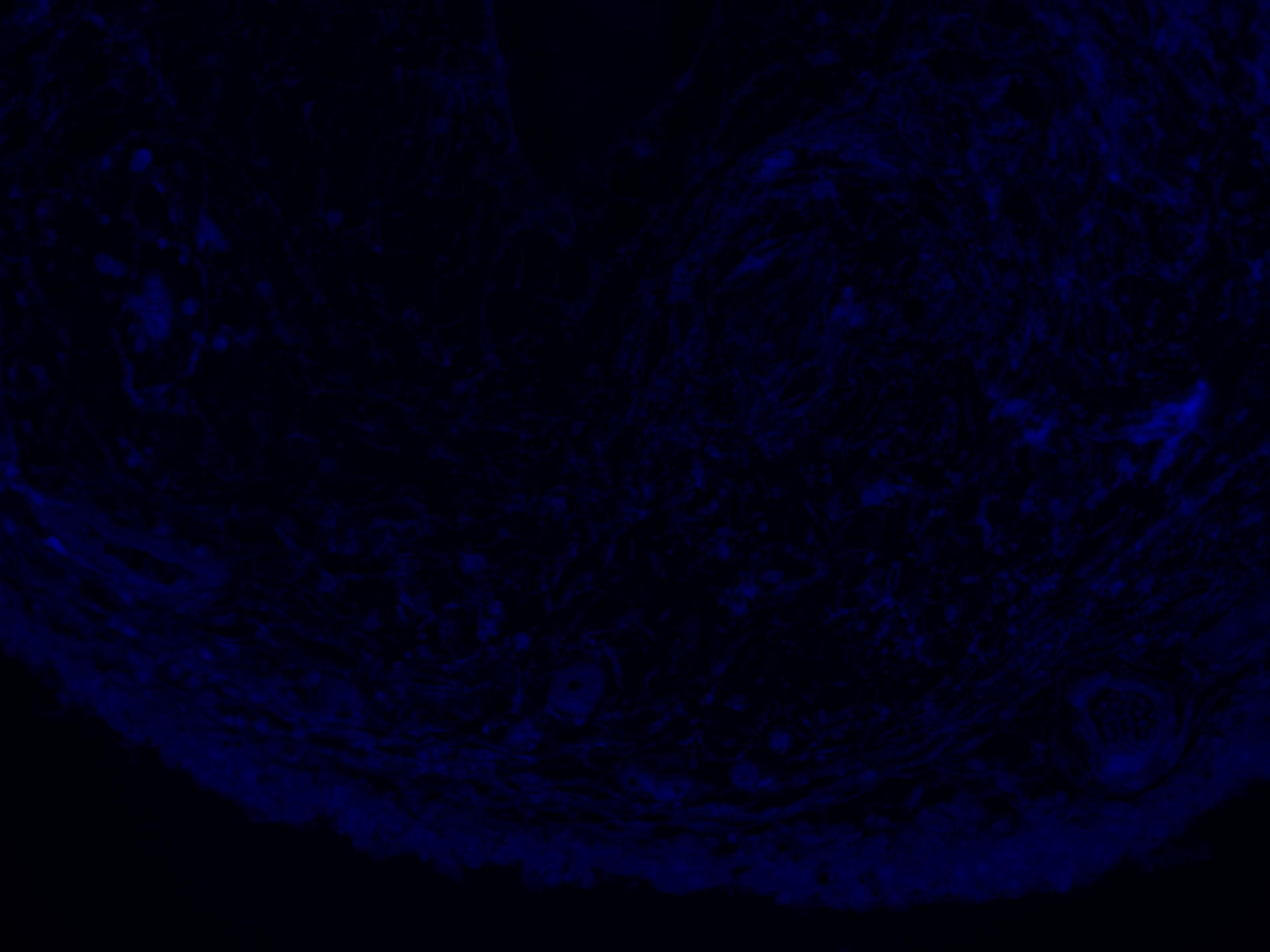

Supplement: S5 File — (ZIP) [file pone.0279584.s019.zip › S5_C files/IF/CD271 EBI3 IRE1/OA/OA IgG/Image_CH3.tif]

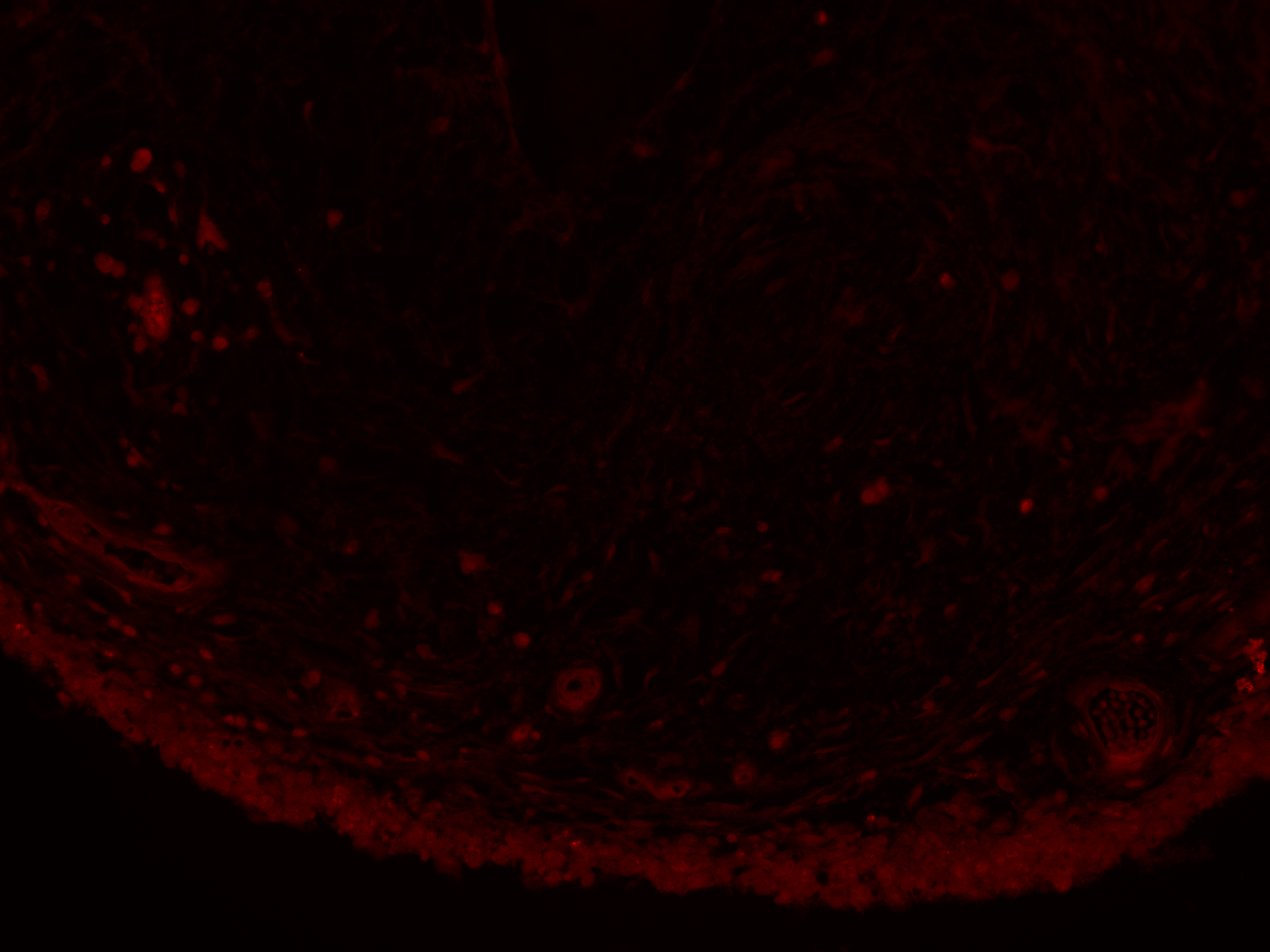

Supplement: S5 File — (ZIP) [file pone.0279584.s019.zip › S5_C files/IF/CD271 EBI3 IRE1/OA/OA IgG/Image_CH2.tif]

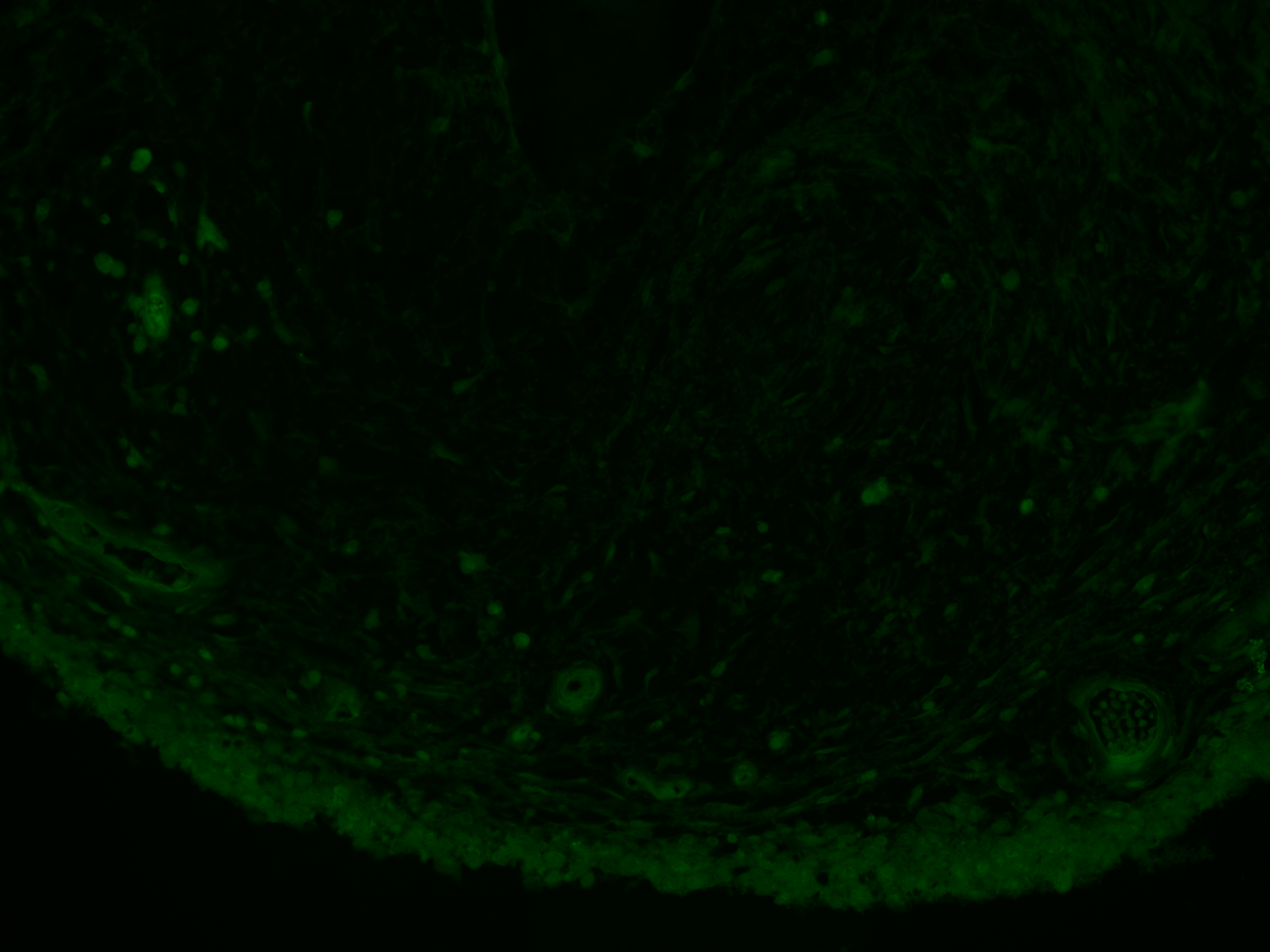

Supplement: S5 File — (ZIP) [file pone.0279584.s019.zip › S5_C files/IF/CD271 EBI3 IRE1/OA/OA IgG/Image_CH1.tif]

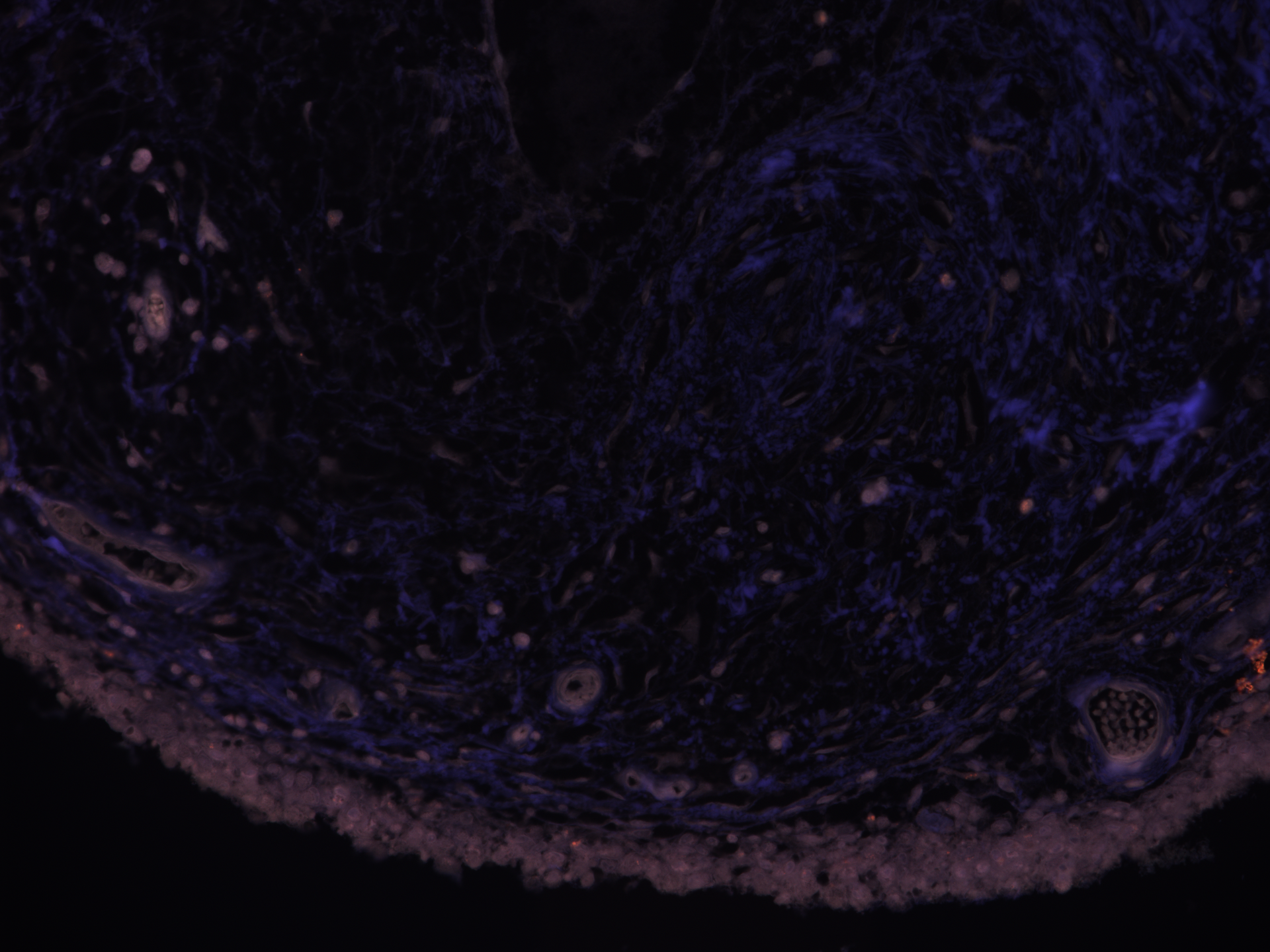

Supplement: S5 File — (ZIP) [file pone.0279584.s019.zip › S5_C files/IF/CD271 EBI3 IRE1/OA/OA IgG/Image_Overlay.tif]

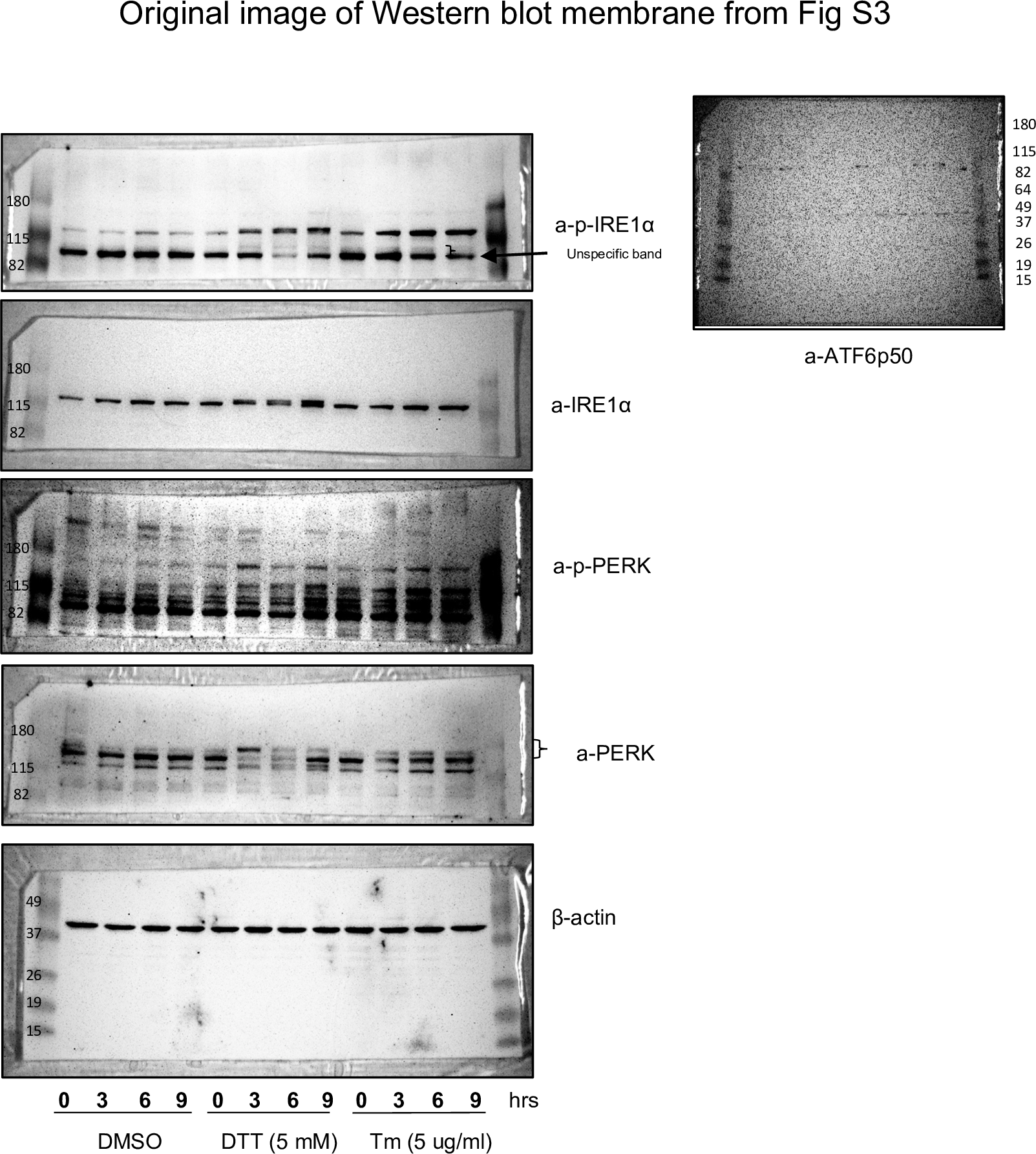

Supplement: S6 File — (ZIP) [file pone.0279584.s020.zip › S6 files/WB S3.tif]

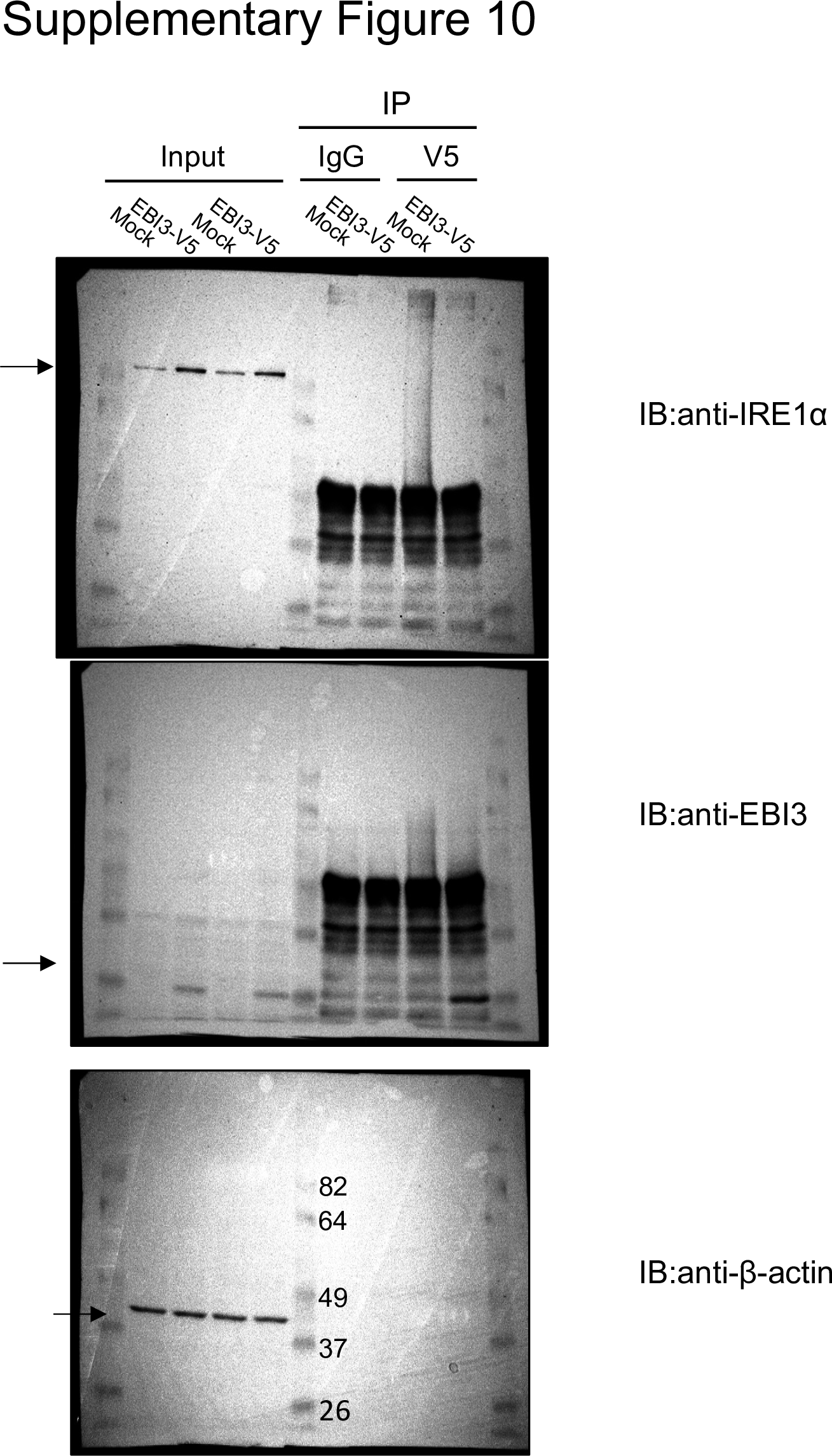

Supplement: S6 File — (ZIP) [file pone.0279584.s020.zip › S6 files/IP S10.tif]

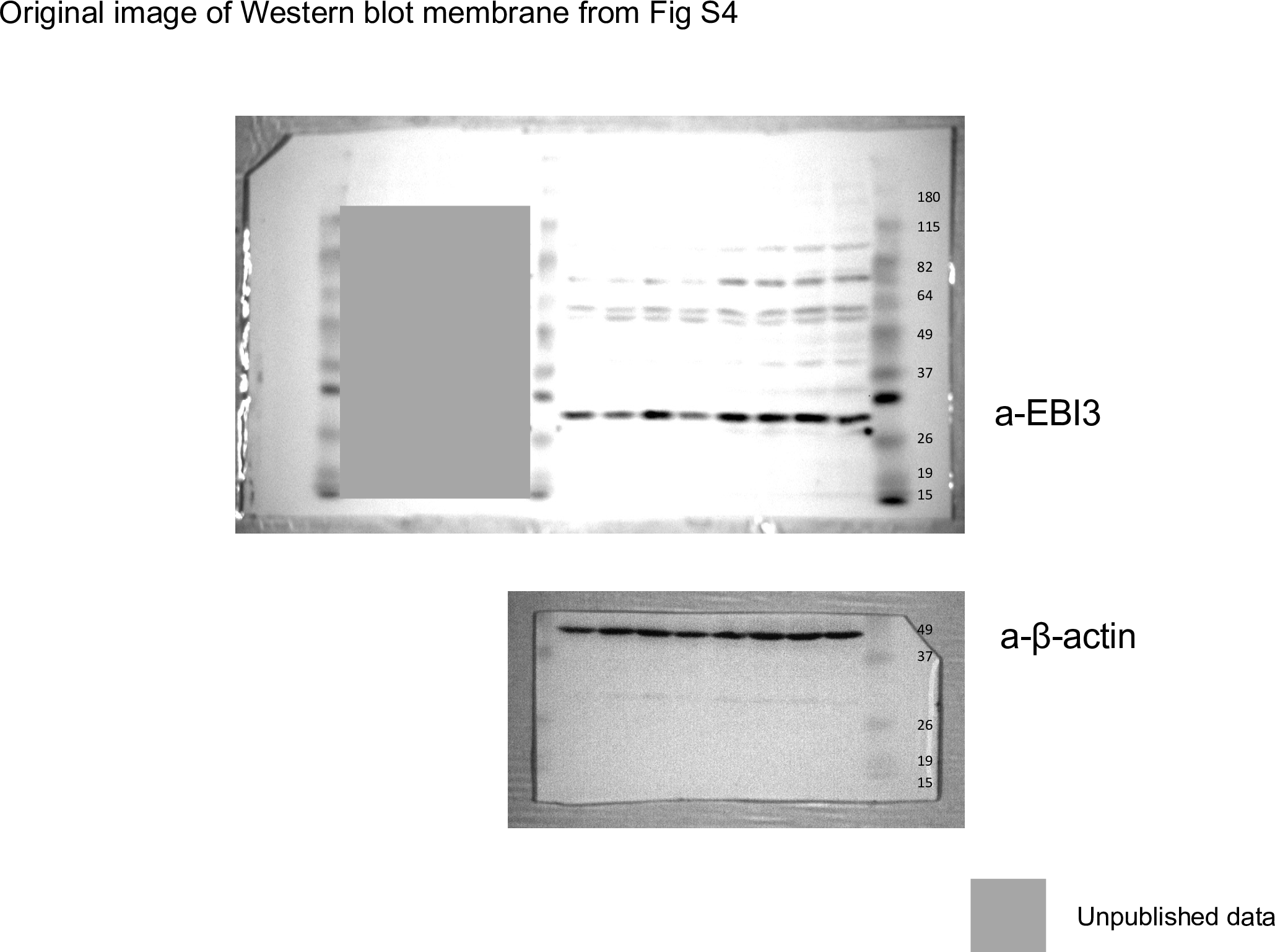

Supplement: S6 File — (ZIP) [file pone.0279584.s020.zip › S6 files/WB S4.tif]

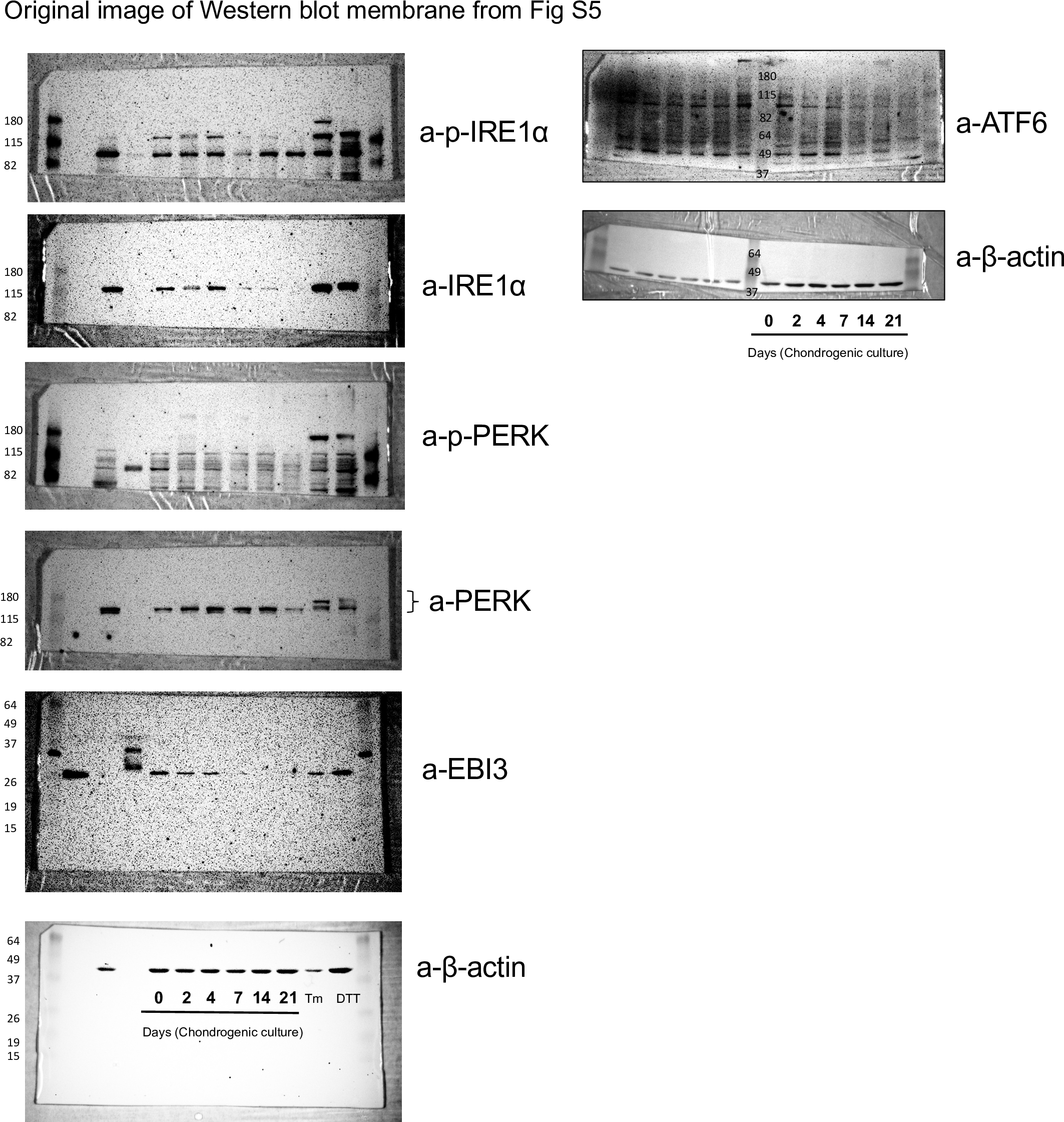

Supplement: S6 File — (ZIP) [file pone.0279584.s020.zip › S6 files/WB S5.tif]

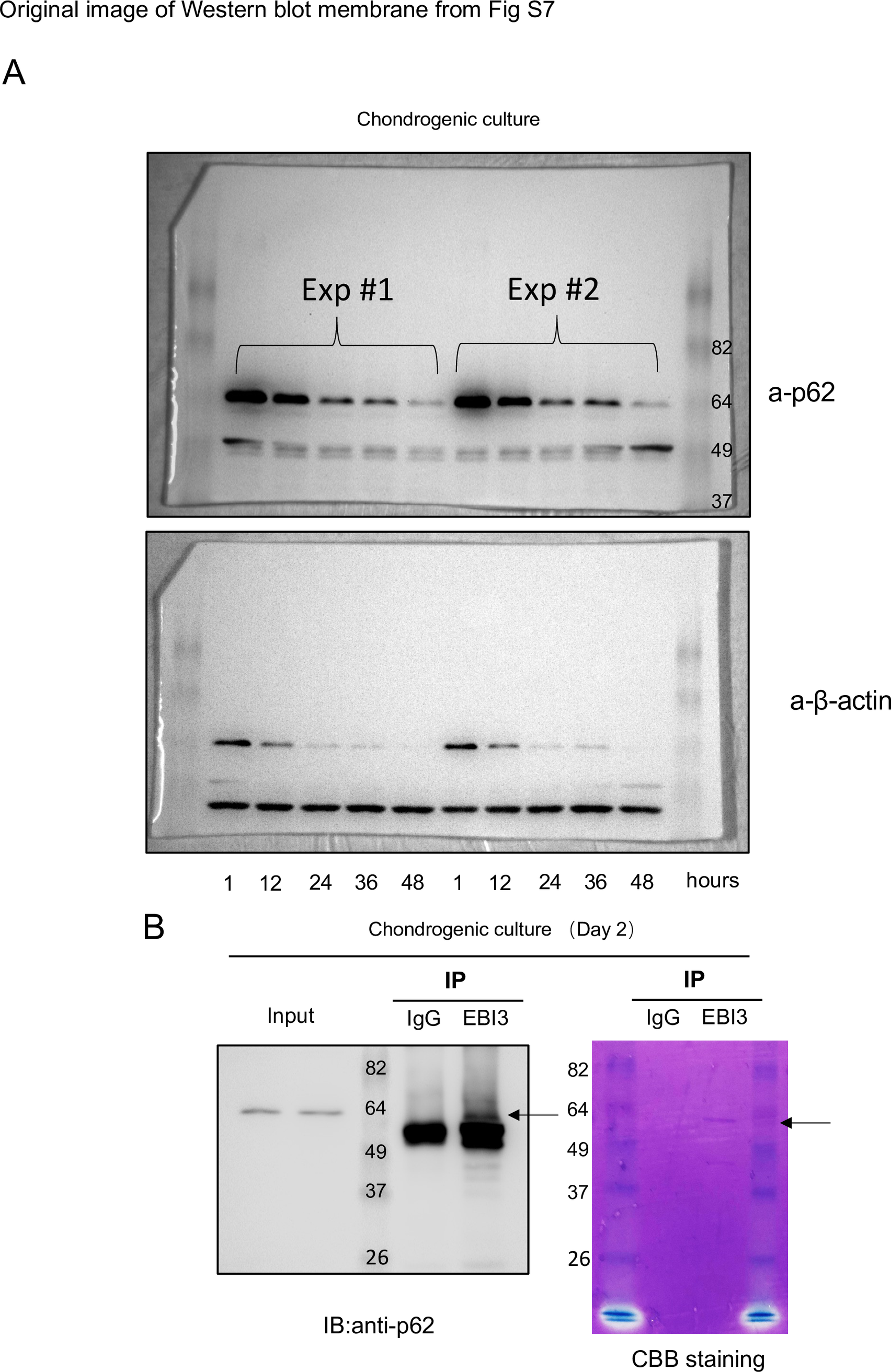

Supplement: S6 File — (ZIP) [file pone.0279584.s020.zip › S6 files/WB IP CBB S7.tif]

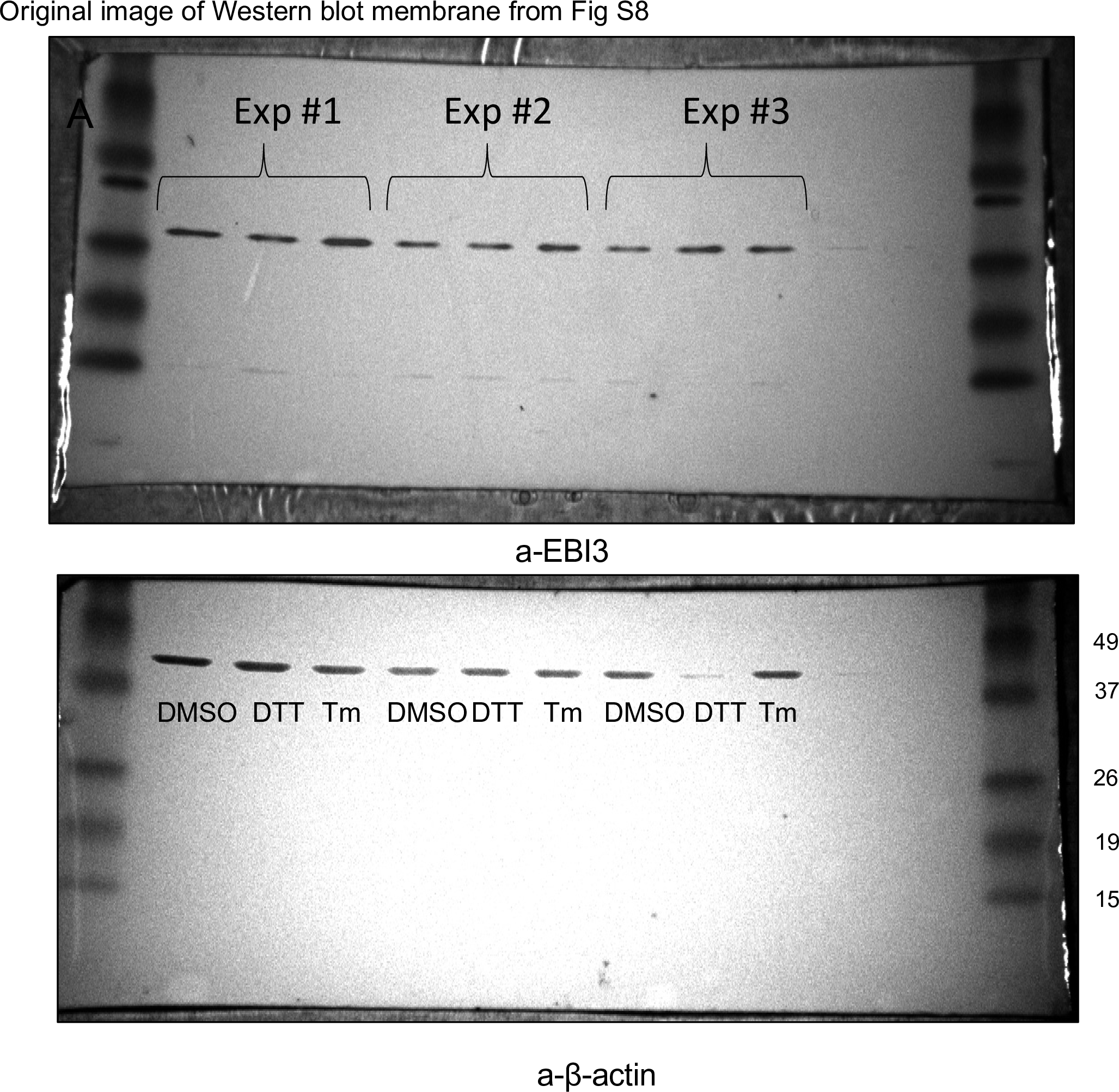

Supplement: S6 File — (ZIP) [file pone.0279584.s020.zip › S6 files/WB S8.tif]

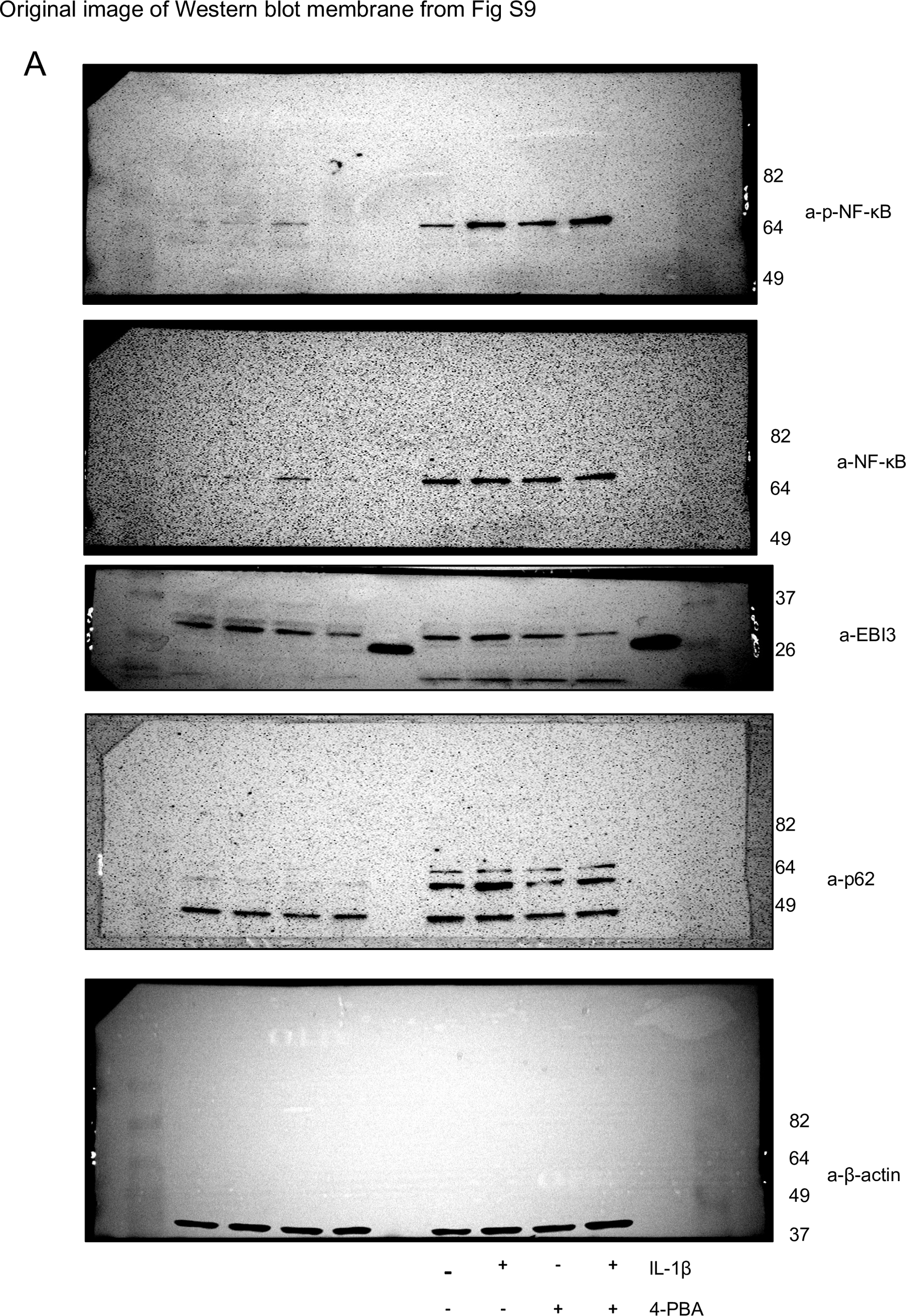

Supplement: S6 File — (ZIP) [file pone.0279584.s020.zip › S6 files/WB S9.tif]

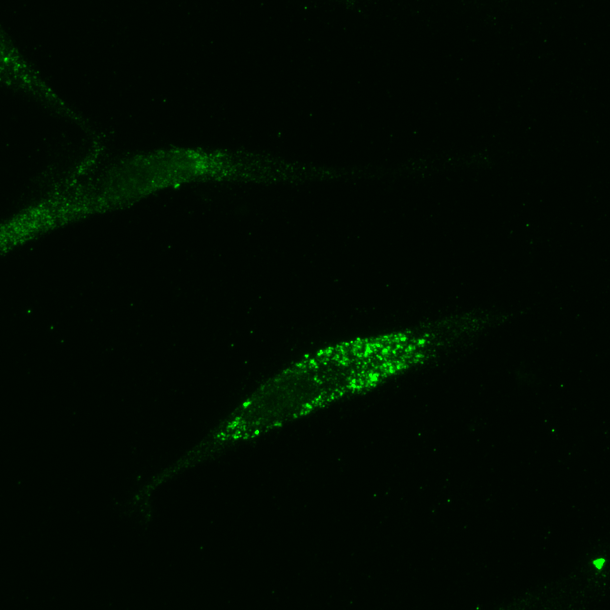

Supplement: S6 File — (ZIP) [file pone.0279584.s020.zip › S6 files/overexpression of EBI3 IF/FITC_Calnexin.tif]

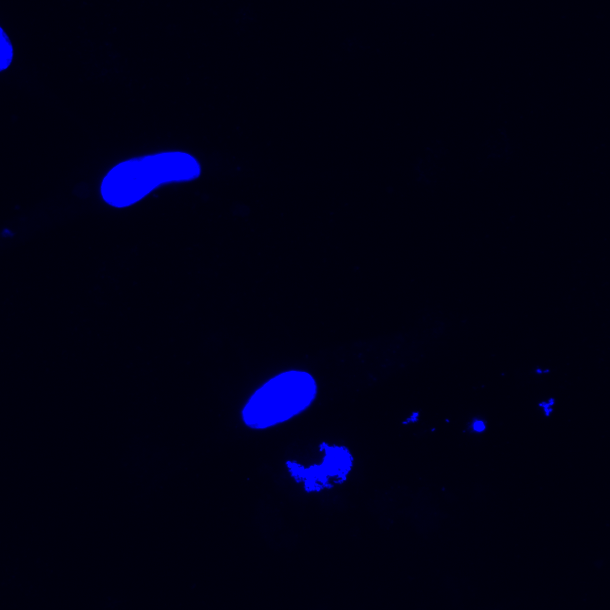

Supplement: S6 File — (ZIP) [file pone.0279584.s020.zip › S6 files/overexpression of EBI3 IF/DAPI.tif]

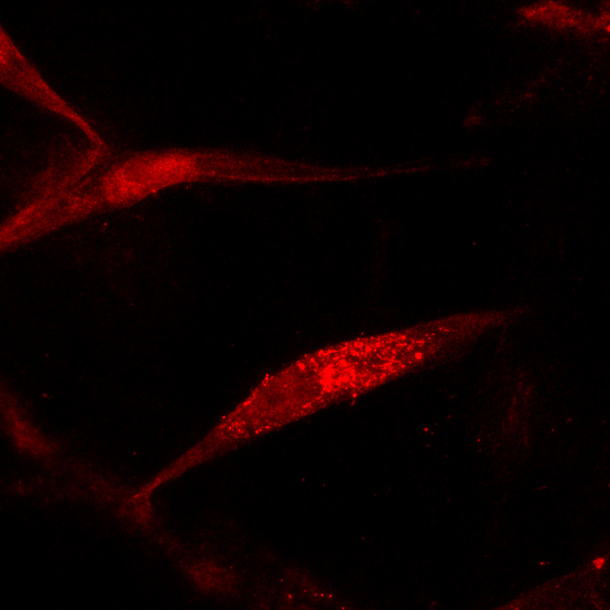

Supplement: S6 File — (ZIP) [file pone.0279584.s020.zip › S6 files/overexpression of EBI3 IF/Rhodamine_EBI3.tif]

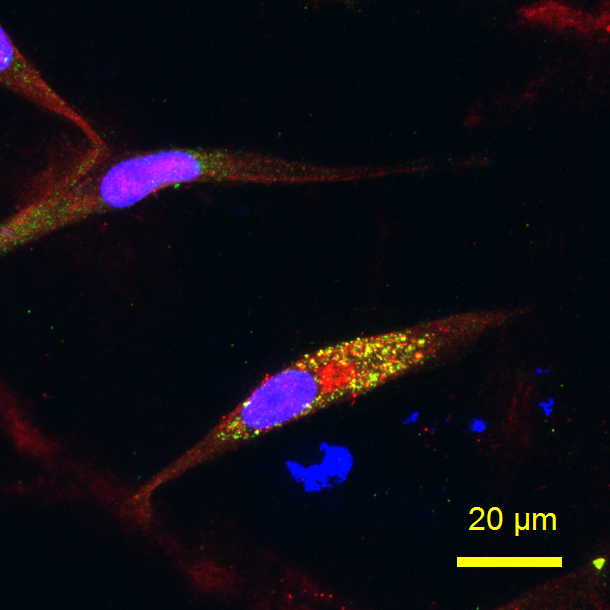

Supplement: S6 File — (ZIP) [file pone.0279584.s020.zip › S6 files/overexpression of EBI3 IF/Image_Overlay.tif]

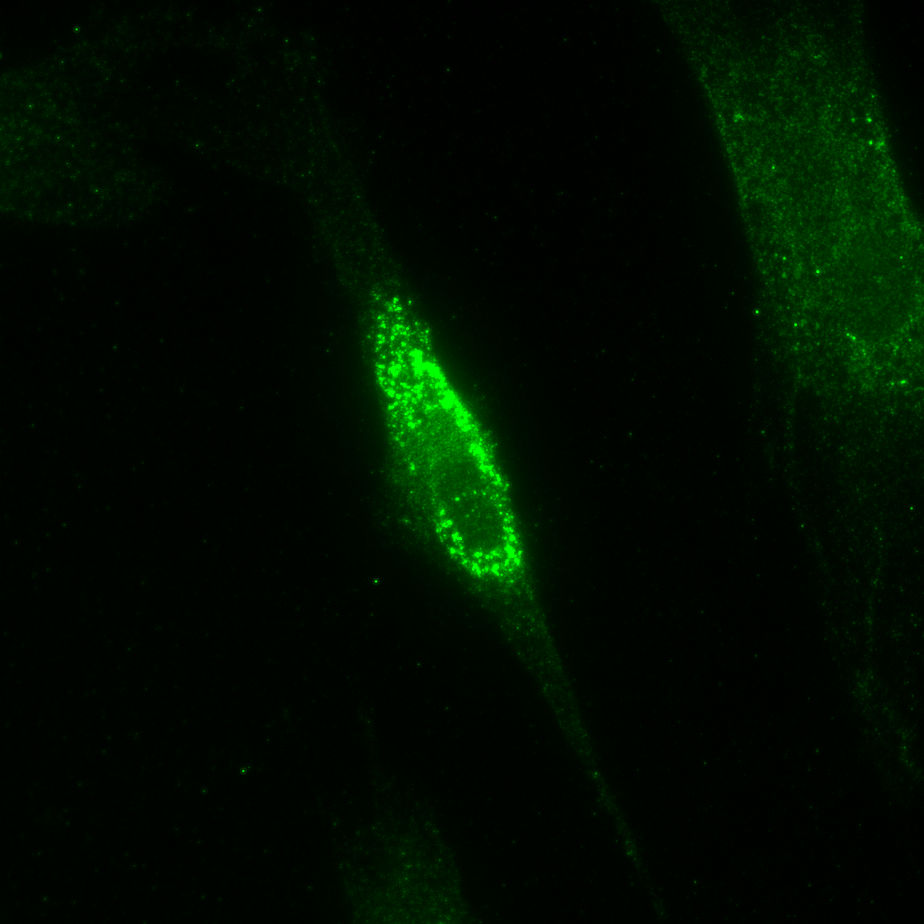

Supplement: S6 File — (ZIP) [file pone.0279584.s020.zip › S6 files/overexpression of EBI3 IF/Mock/FITC_Calnexin.tif]

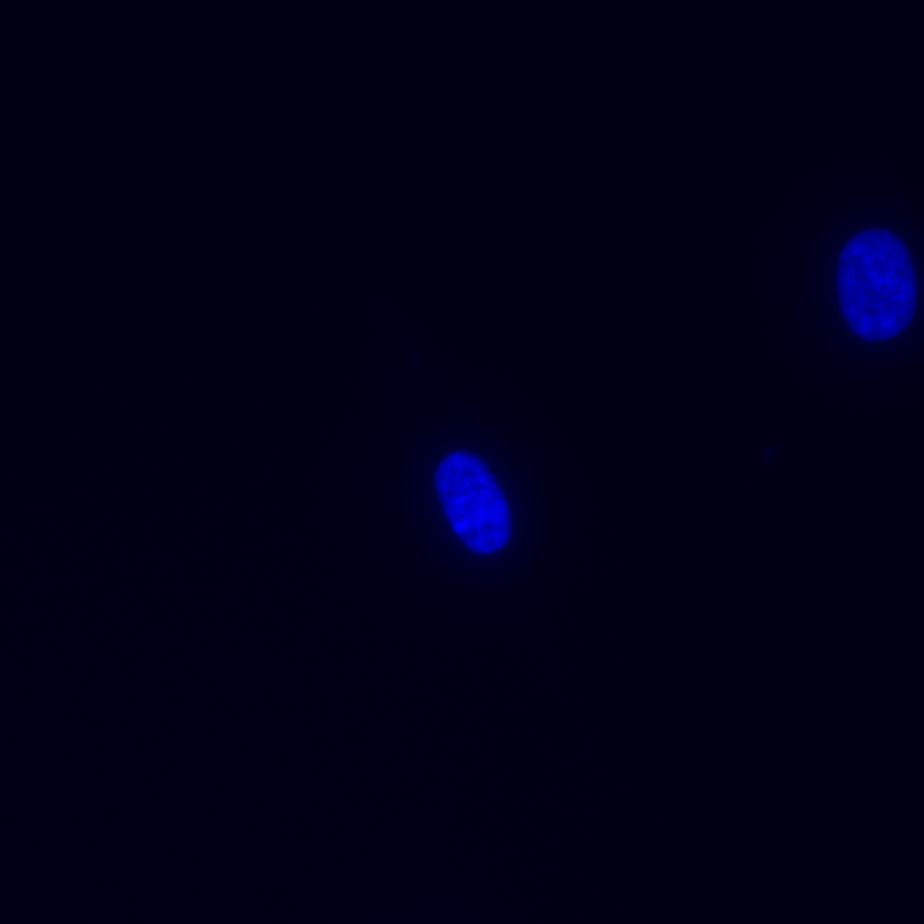

Supplement: S6 File — (ZIP) [file pone.0279584.s020.zip › S6 files/overexpression of EBI3 IF/Mock/DAPI.tif]

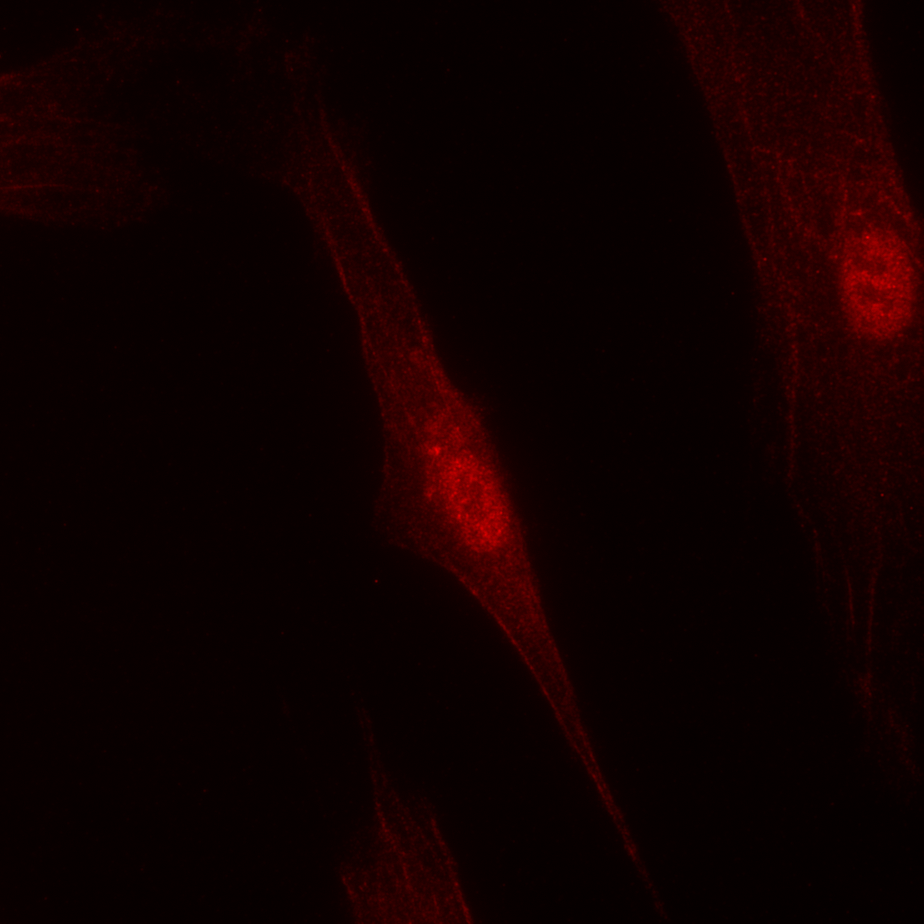

Supplement: S6 File — (ZIP) [file pone.0279584.s020.zip › S6 files/overexpression of EBI3 IF/Mock/Rhodamine_EBI3.tif]

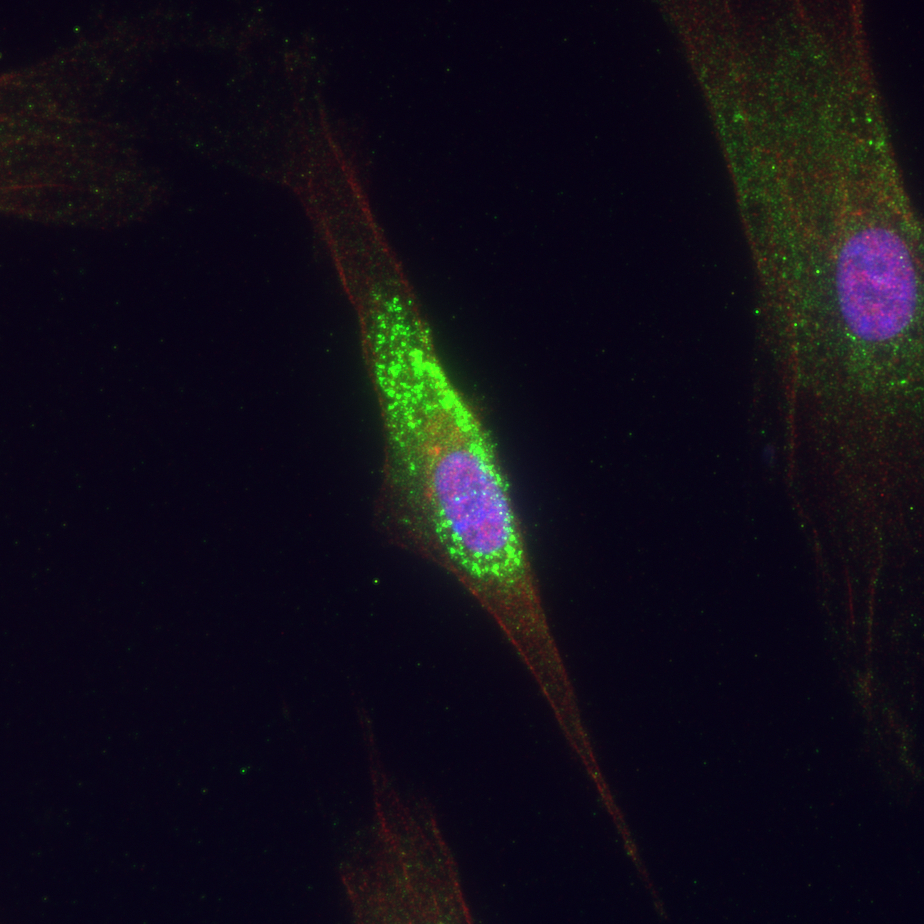

Supplement: S6 File — (ZIP) [file pone.0279584.s020.zip › S6 files/overexpression of EBI3 IF/Mock/Image_Overlay.tif]

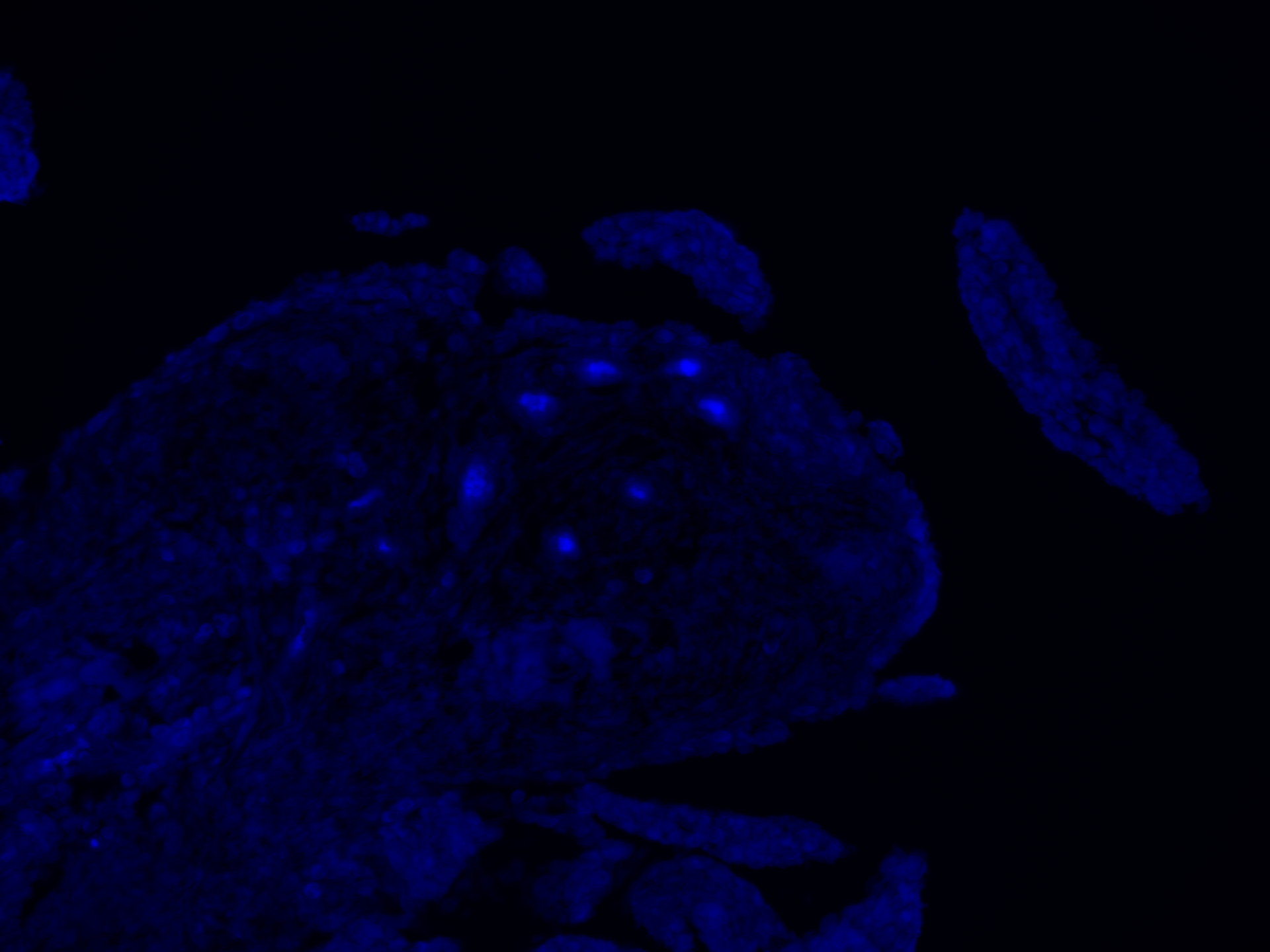

Supplement: S6 File — (ZIP) [file pone.0279584.s020.zip › S6 files/synovium IF/CD271 CD105 EBI3/RA/Alexa Fluor 350_CD271.tif]

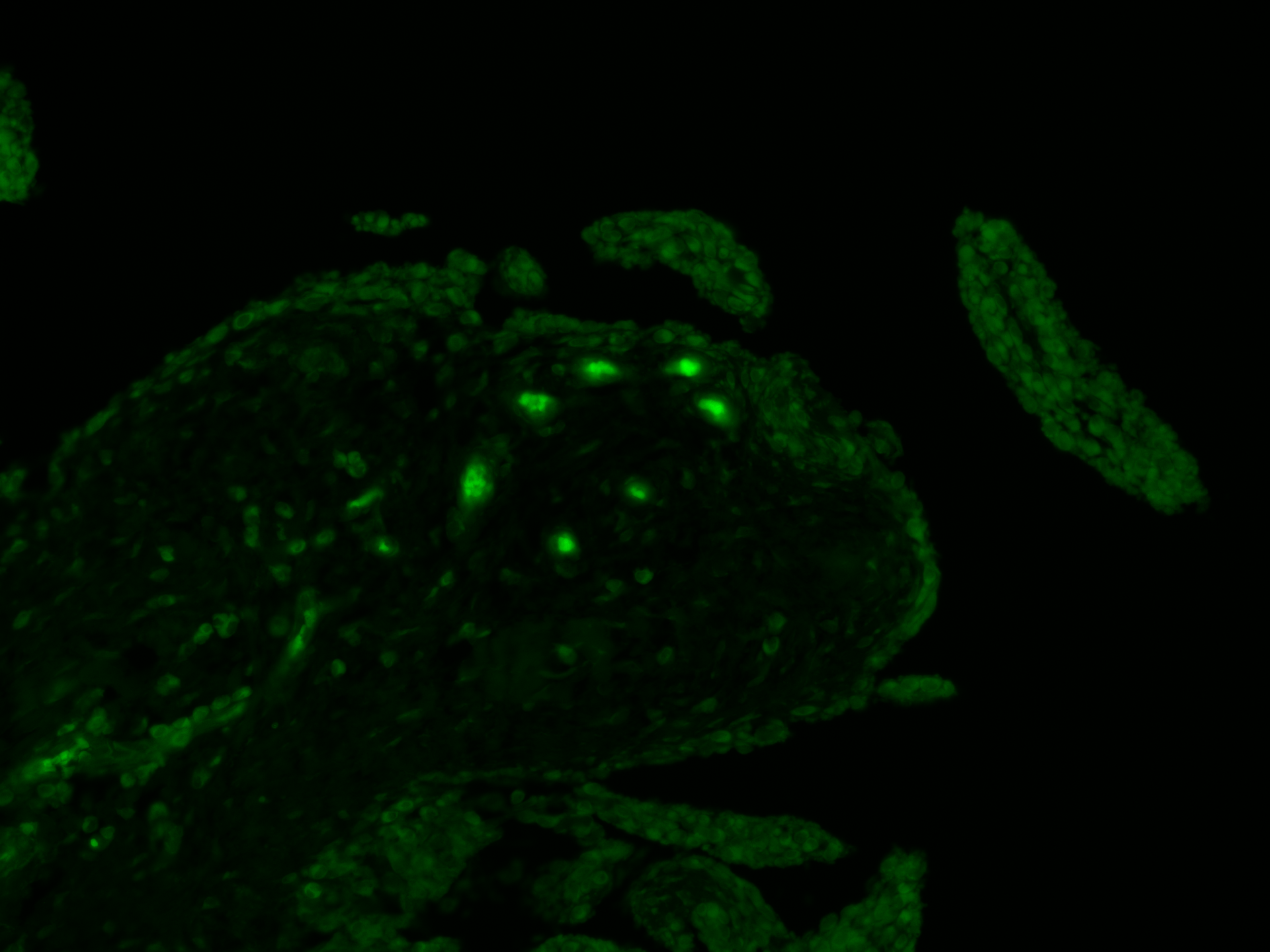

Supplement: S6 File — (ZIP) [file pone.0279584.s020.zip › S6 files/synovium IF/CD271 CD105 EBI3/RA/Alexa Fluor 488_CD105.tif]

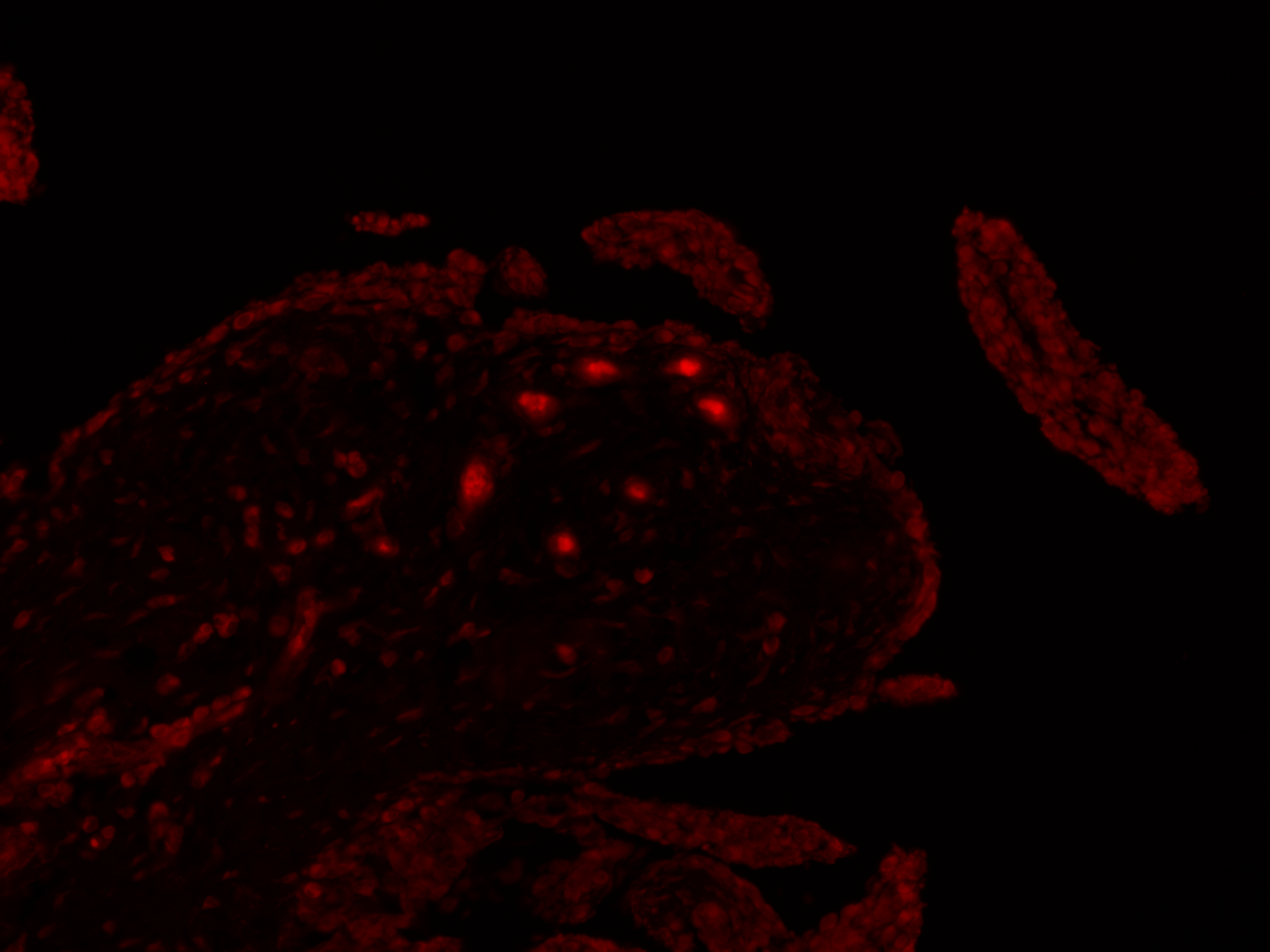

Supplement: S6 File — (ZIP) [file pone.0279584.s020.zip › S6 files/synovium IF/CD271 CD105 EBI3/RA/Alexa Fluor 568_EBI3.tif]

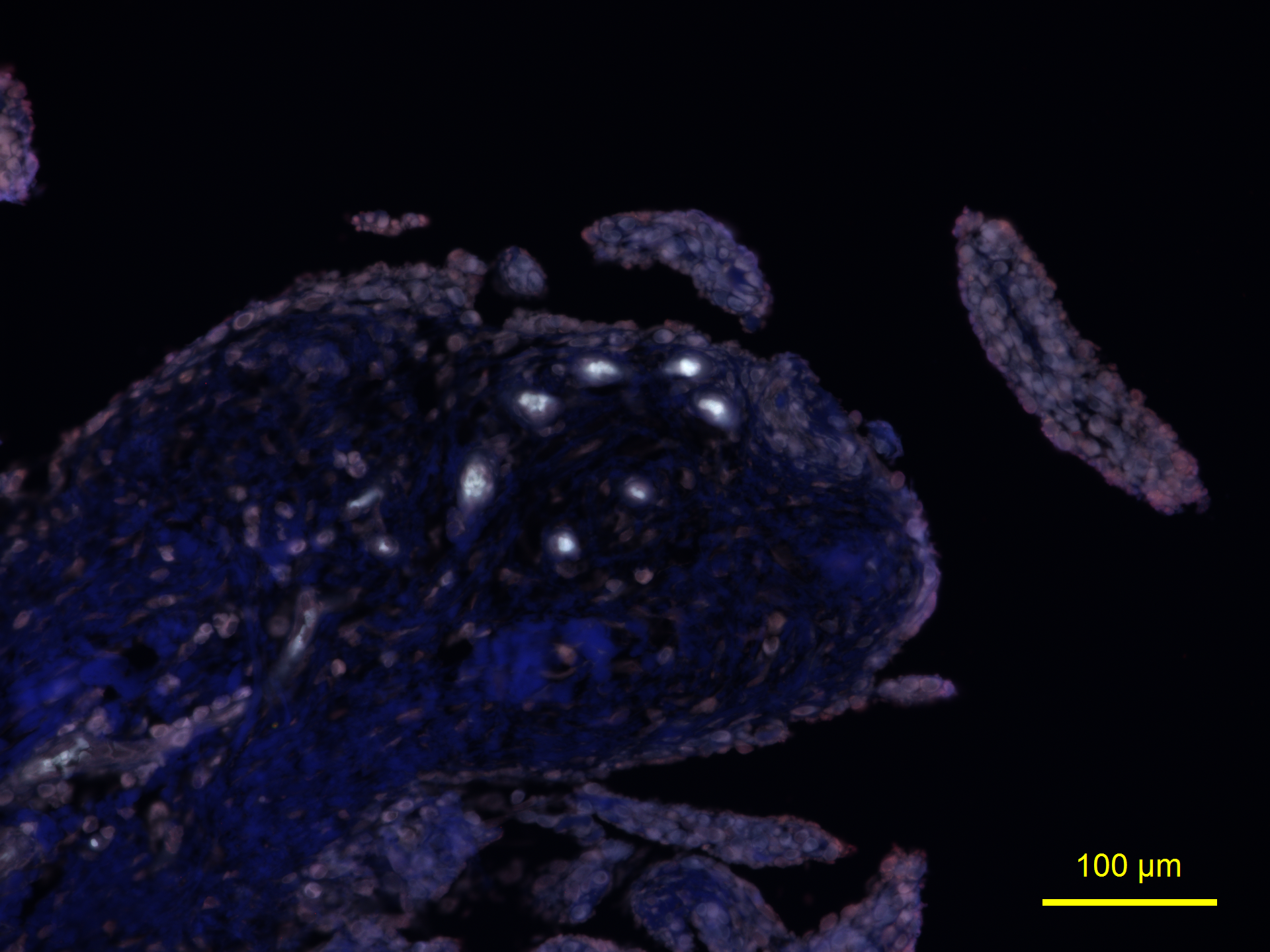

Supplement: S6 File — (ZIP) [file pone.0279584.s020.zip › S6 files/synovium IF/CD271 CD105 EBI3/RA/Image_Overlay.tif]

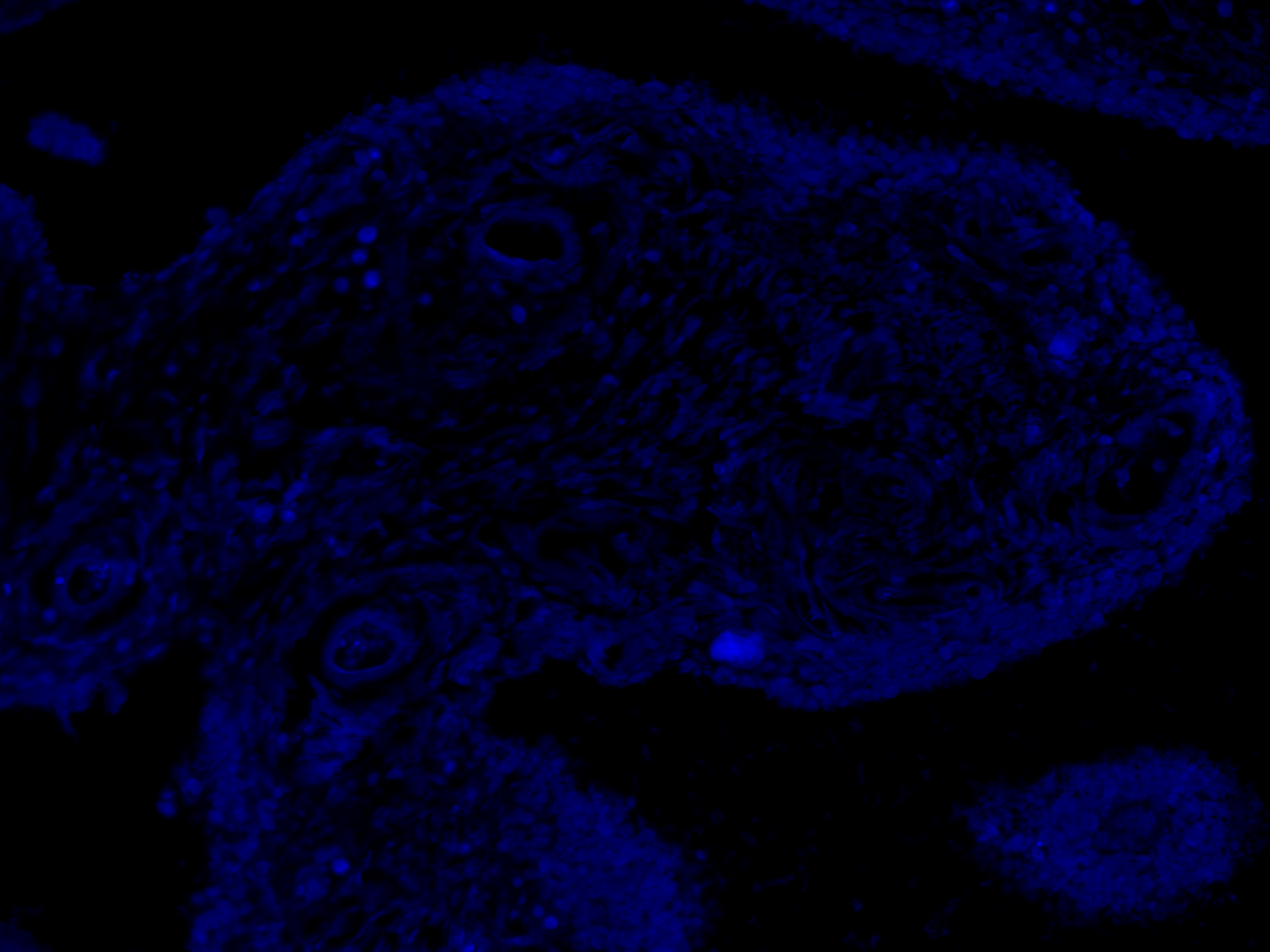

Supplement: S6 File — (ZIP) [file pone.0279584.s020.zip › S6 files/synovium IF/CD271 CD105 EBI3/OA/Image_CH3.tif]

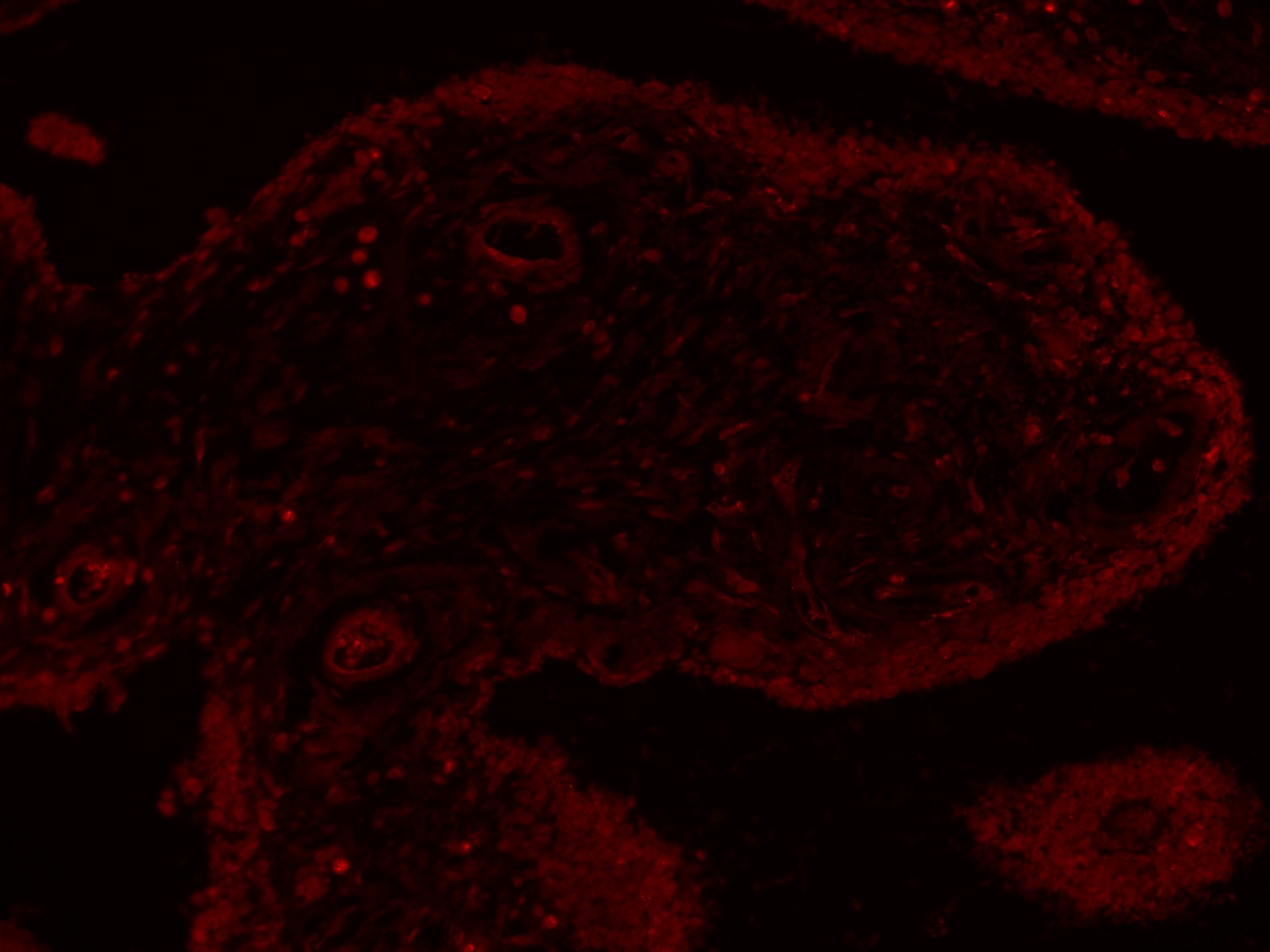

Supplement: S6 File — (ZIP) [file pone.0279584.s020.zip › S6 files/synovium IF/CD271 CD105 EBI3/OA/Image_CH2.tif]

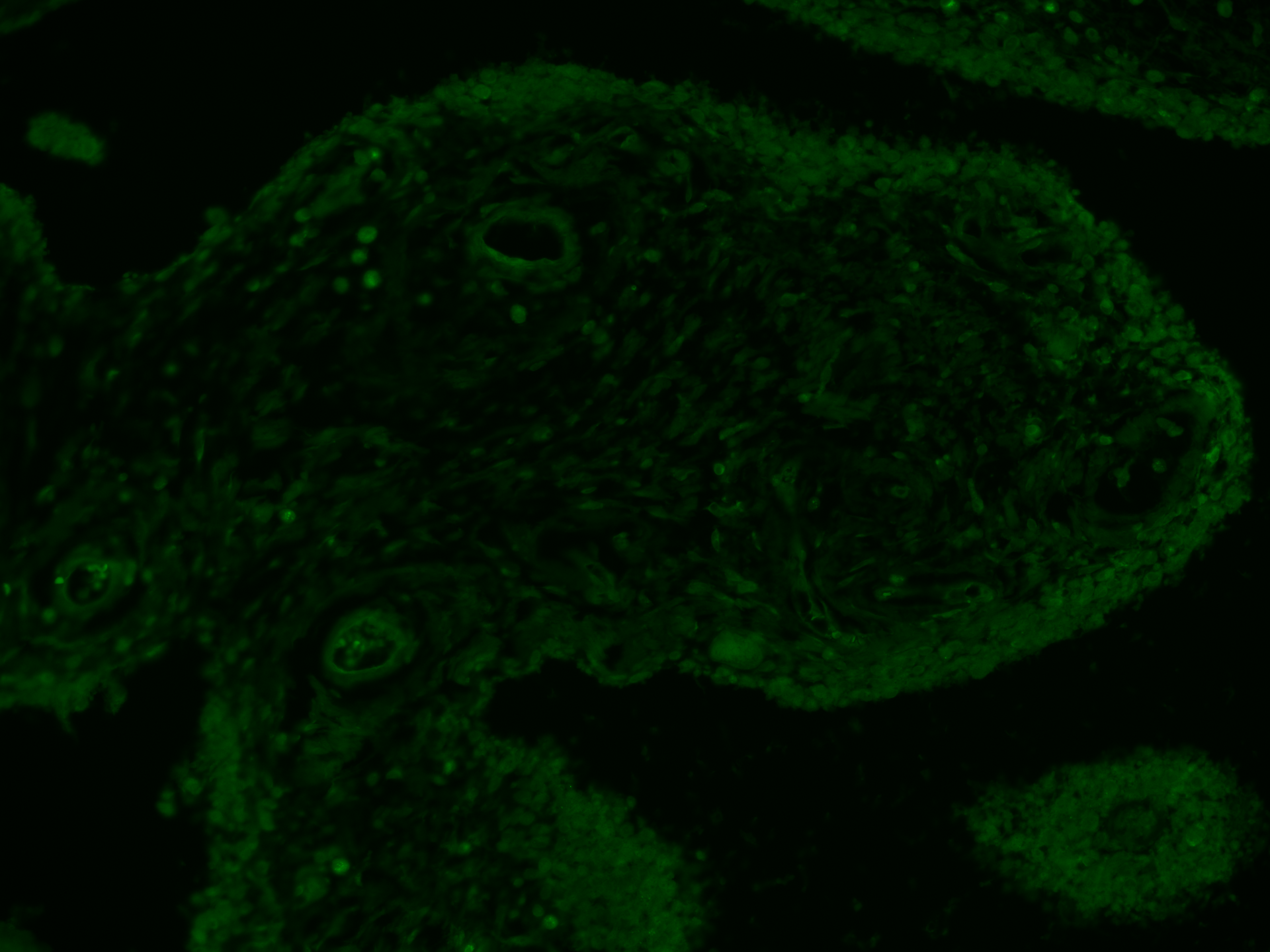

Supplement: S6 File — (ZIP) [file pone.0279584.s020.zip › S6 files/synovium IF/CD271 CD105 EBI3/OA/Image_CH1.tif]

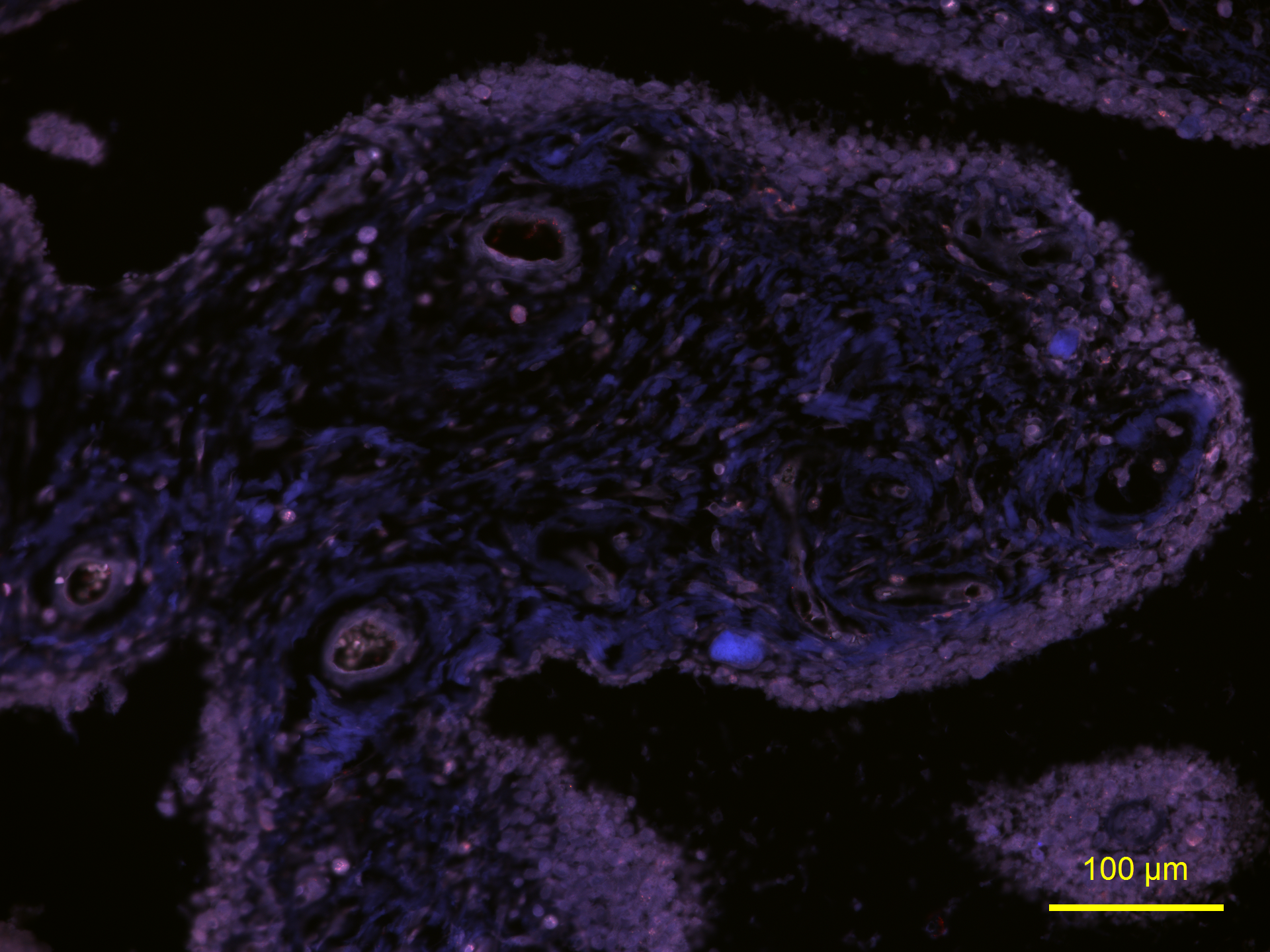

Supplement: S6 File — (ZIP) [file pone.0279584.s020.zip › S6 files/synovium IF/CD271 CD105 EBI3/OA/Image_Overlay bar.tif]

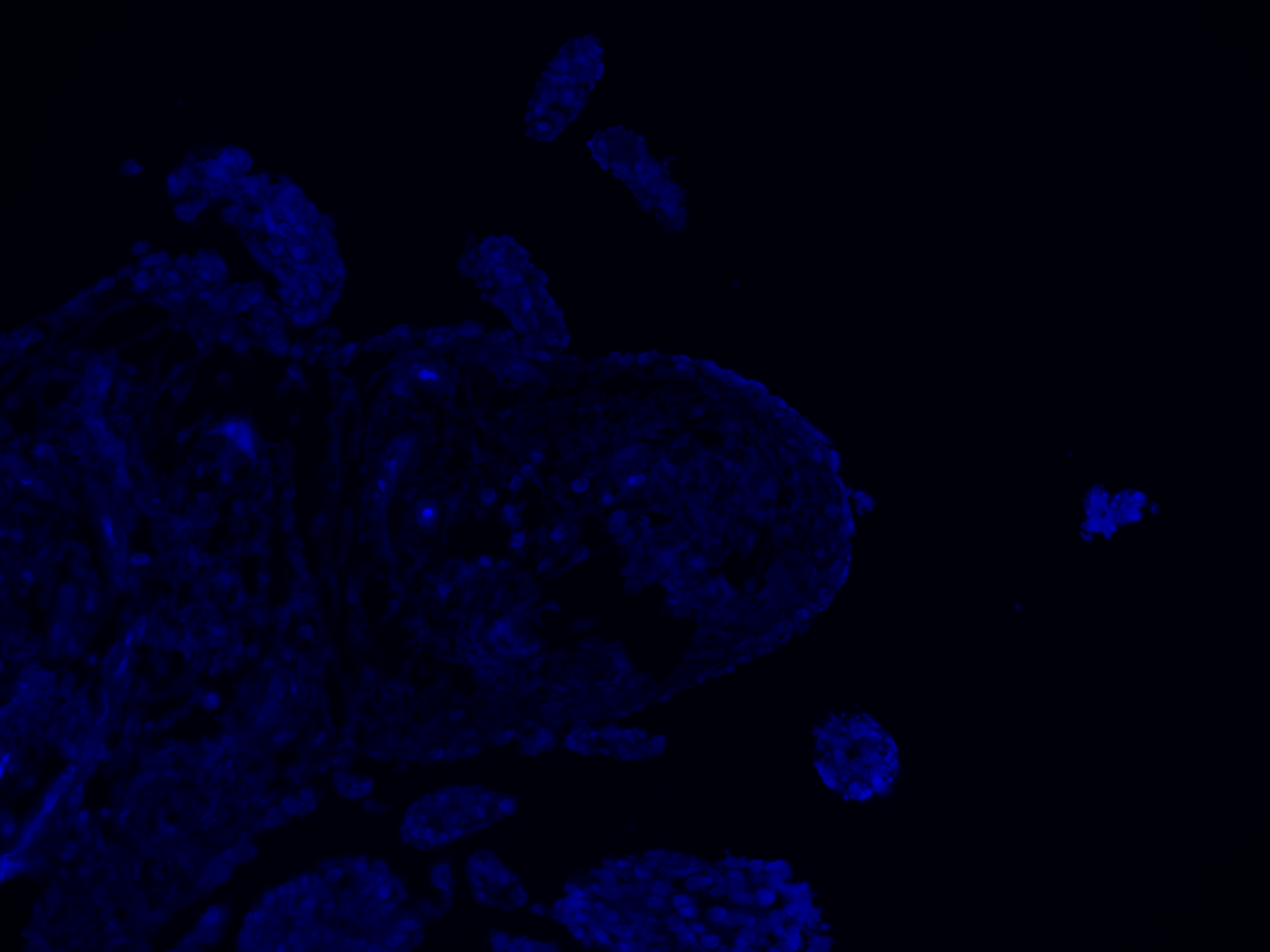

Supplement: S6 File — (ZIP) [file pone.0279584.s020.zip › S6 files/synovium IF/CD271 CD105 EBI3/RA/IgG/Image_CH3.tif]

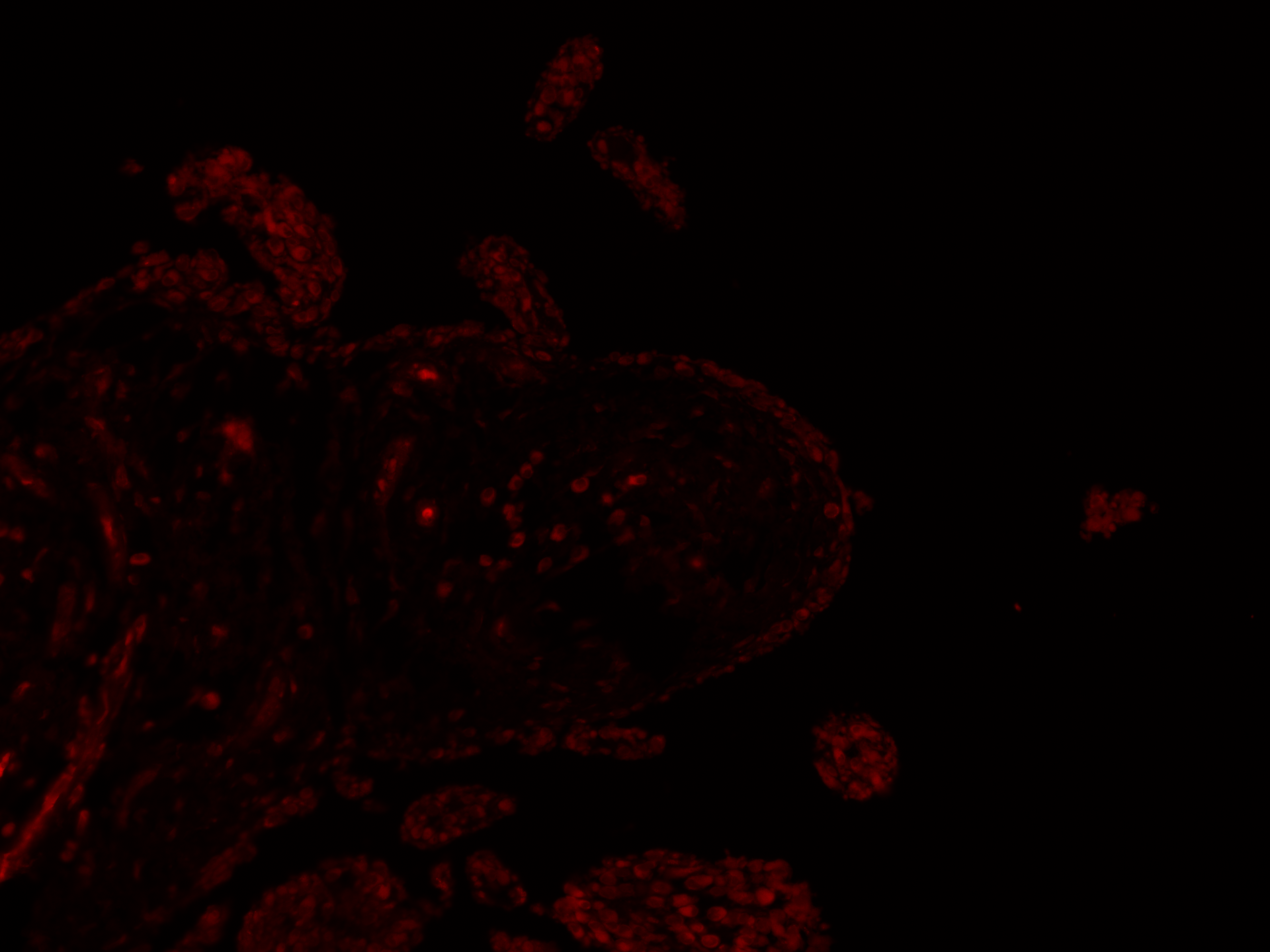

Supplement: S6 File — (ZIP) [file pone.0279584.s020.zip › S6 files/synovium IF/CD271 CD105 EBI3/RA/IgG/Image_CH2.tif]

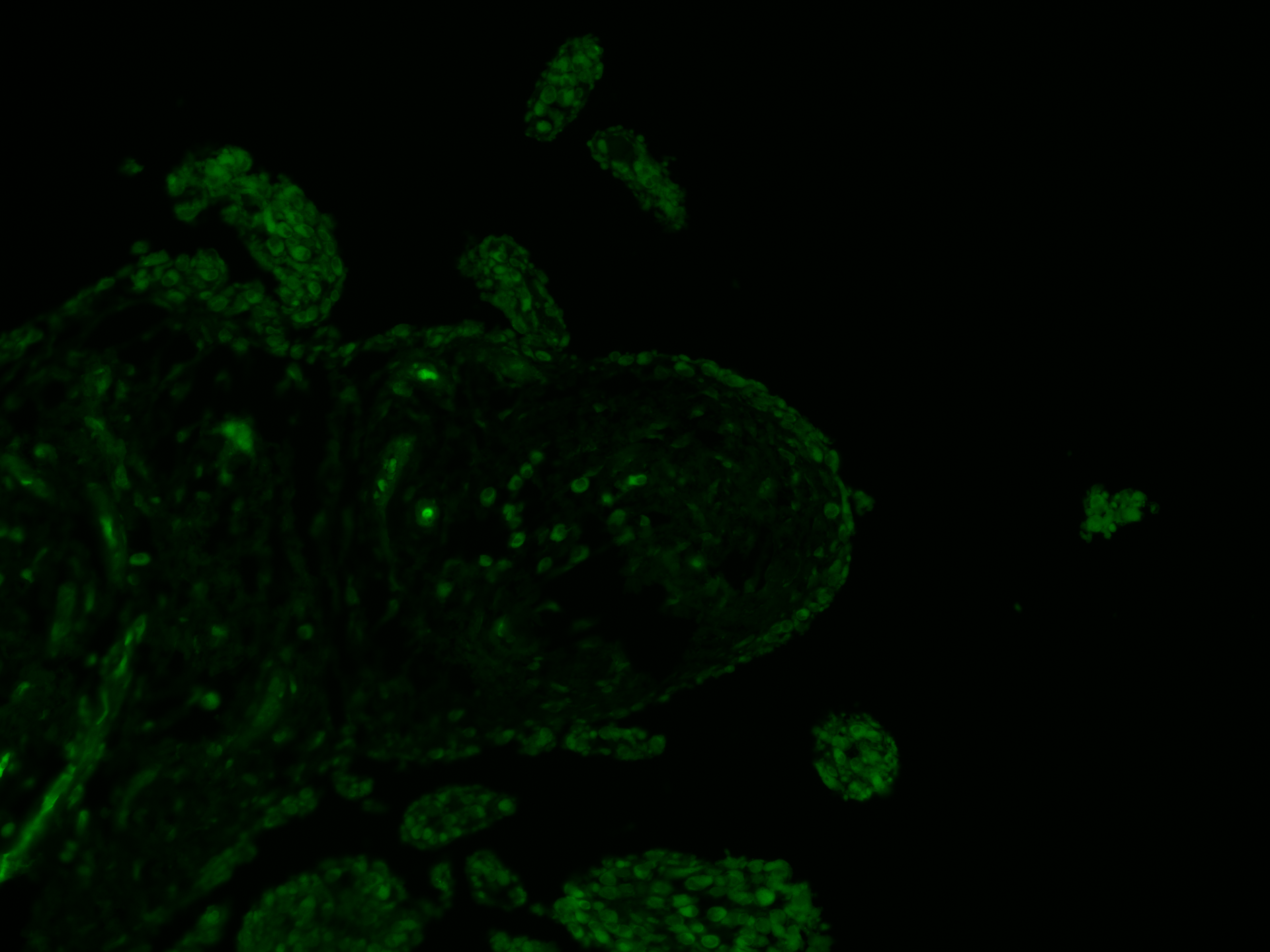

Supplement: S6 File — (ZIP) [file pone.0279584.s020.zip › S6 files/synovium IF/CD271 CD105 EBI3/RA/IgG/Image_CH1.tif]

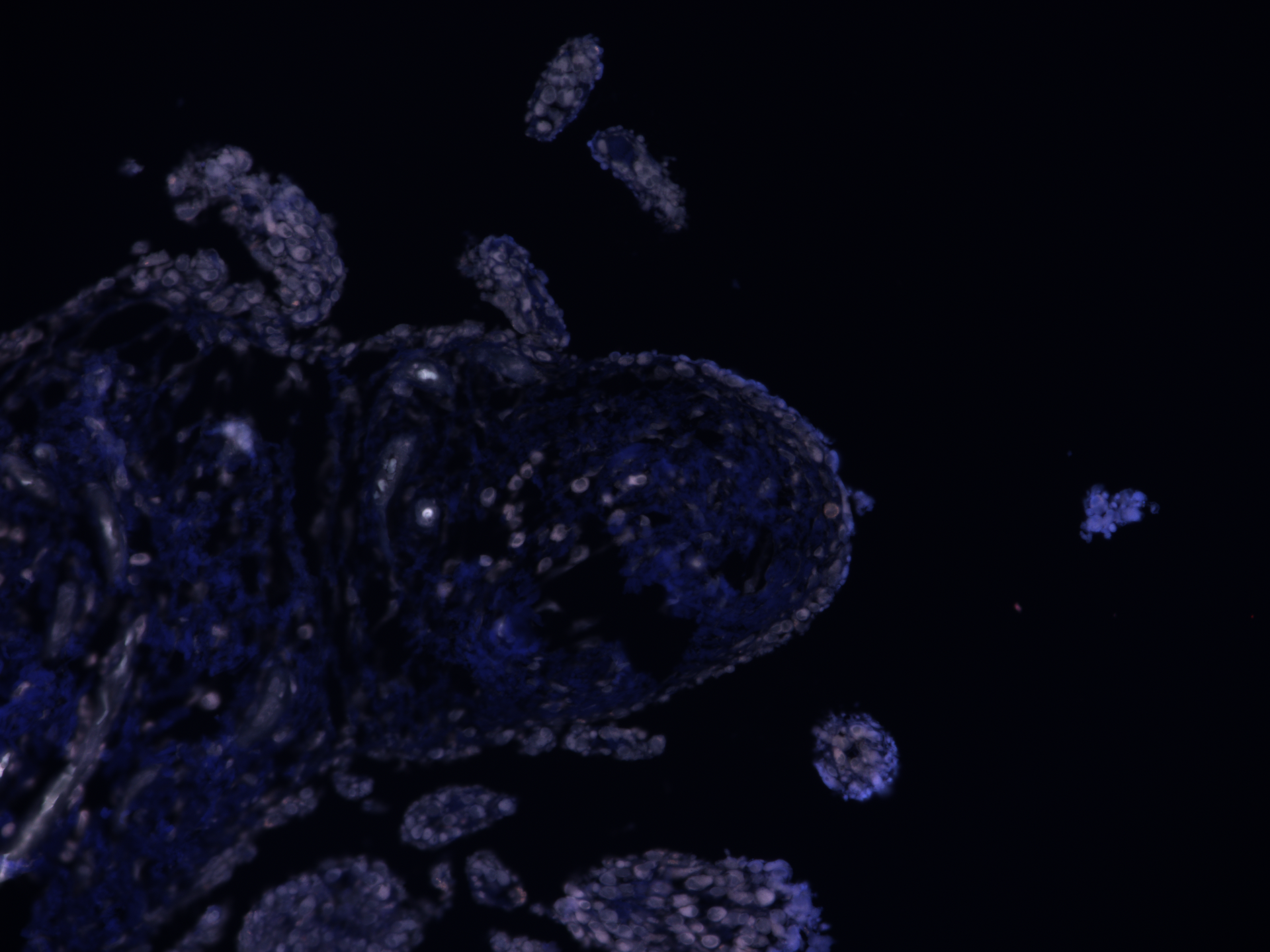

Supplement: S6 File — (ZIP) [file pone.0279584.s020.zip › S6 files/synovium IF/CD271 CD105 EBI3/RA/IgG/Image_Overlay.tif]

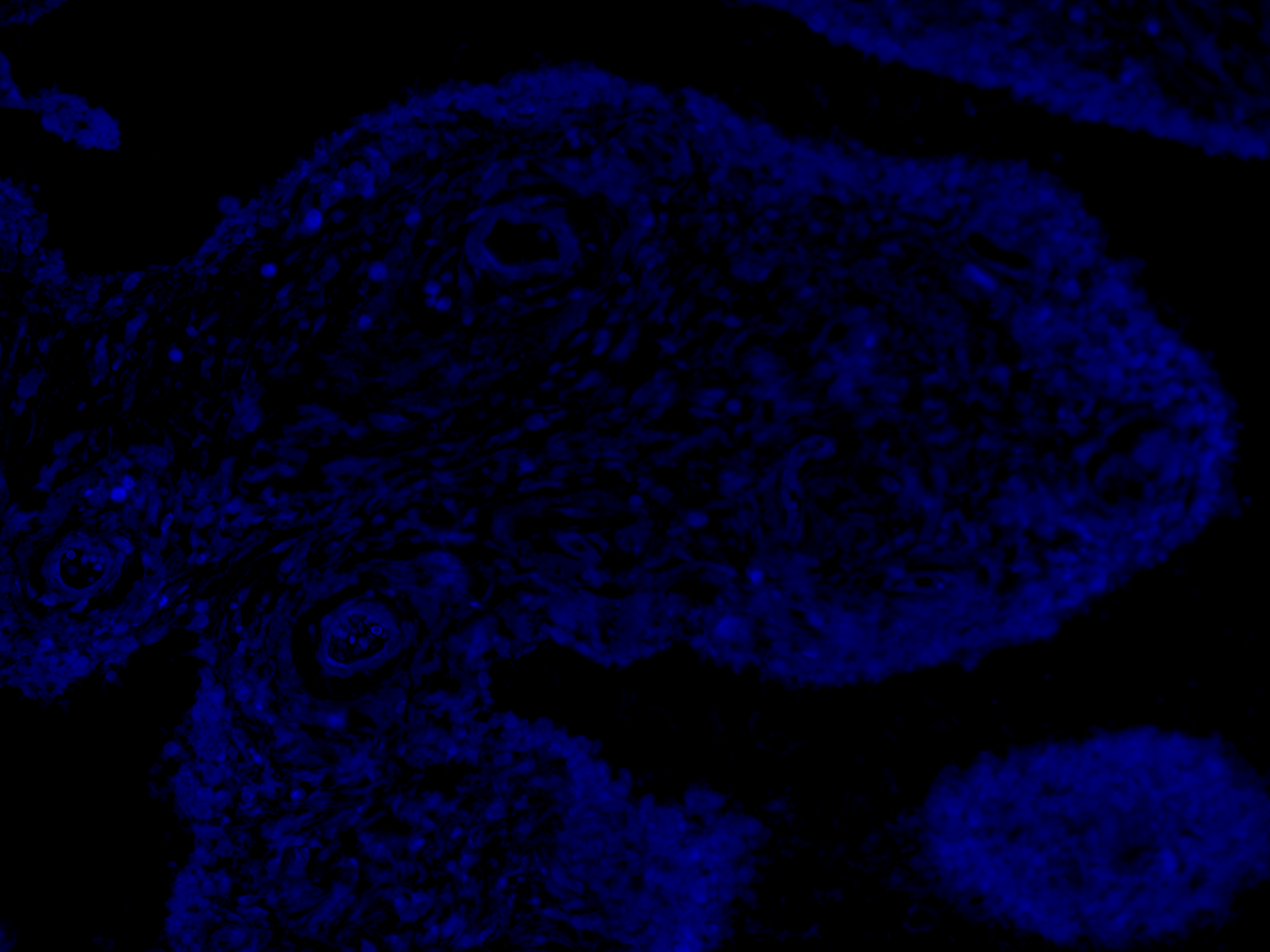

Supplement: S6 File — (ZIP) [file pone.0279584.s020.zip › S6 files/synovium IF/CD271 CD105 EBI3/OA/IgG/Image_CH3.tif]

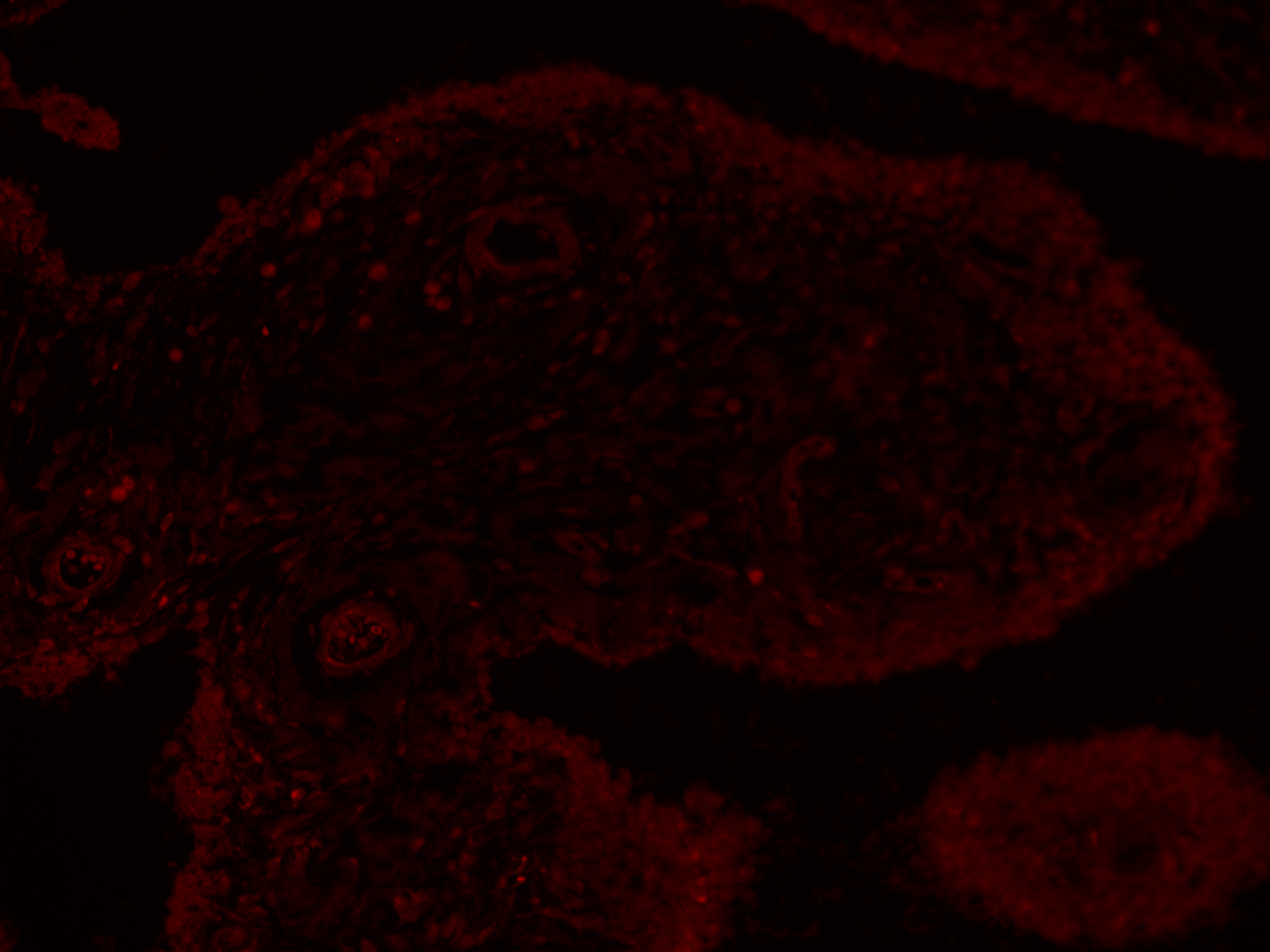

Supplement: S6 File — (ZIP) [file pone.0279584.s020.zip › S6 files/synovium IF/CD271 CD105 EBI3/OA/IgG/Image_CH2.tif]

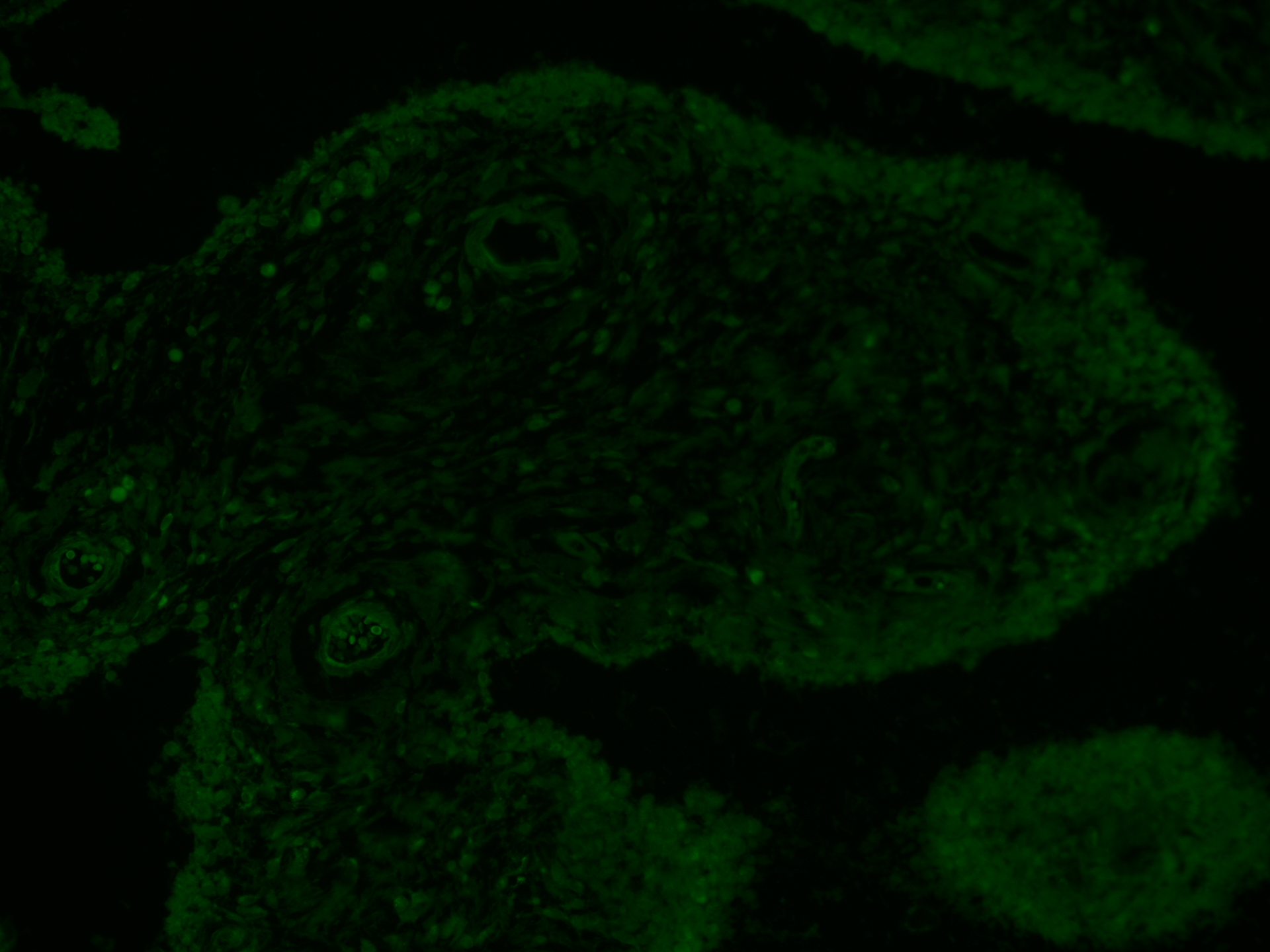

Supplement: S6 File — (ZIP) [file pone.0279584.s020.zip › S6 files/synovium IF/CD271 CD105 EBI3/OA/IgG/Image_CH1.tif]

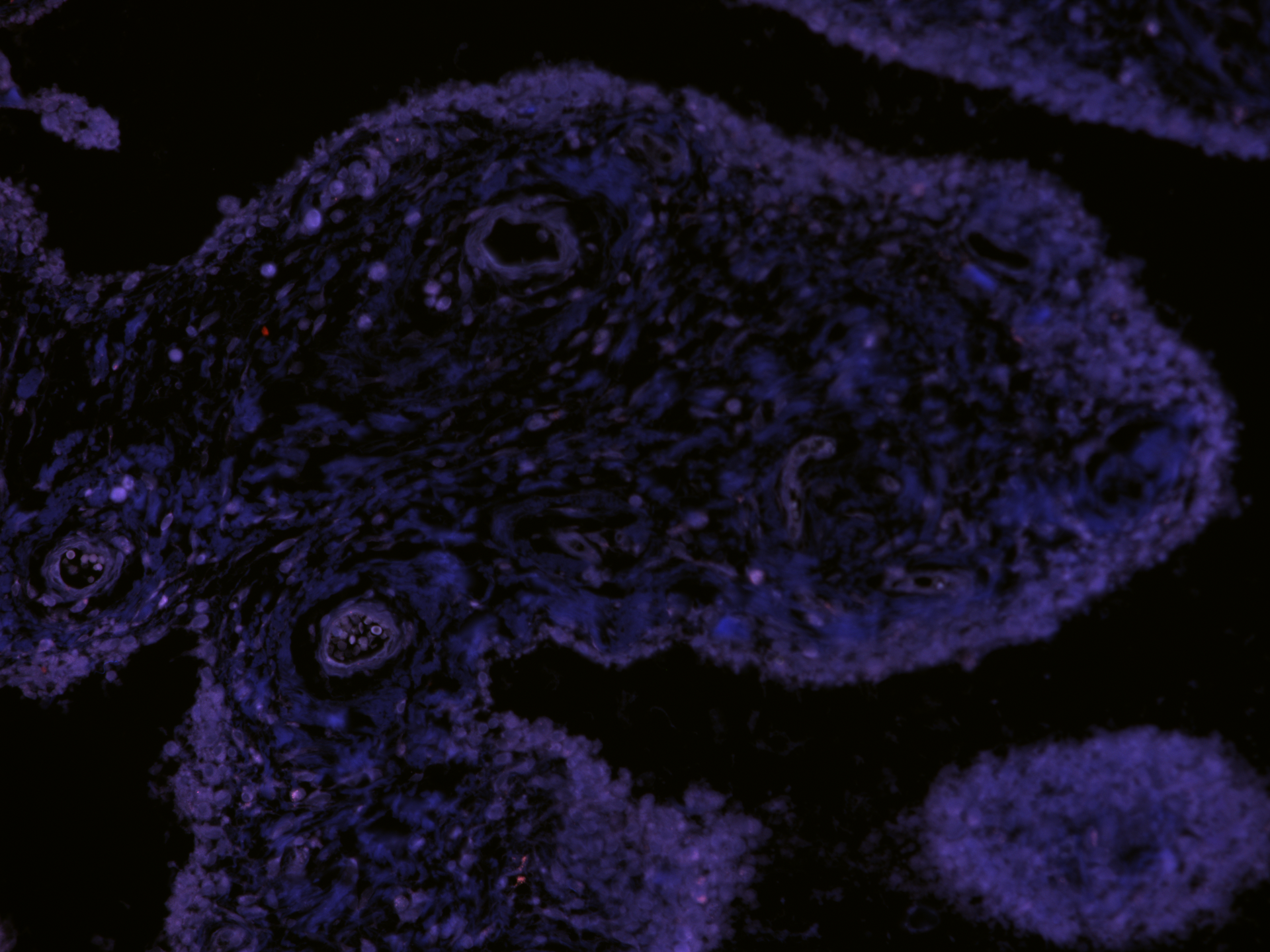

Supplement: S6 File — (ZIP) [file pone.0279584.s020.zip › S6 files/synovium IF/CD271 CD105 EBI3/OA/IgG/Image_Overlay.tif]
