# Supplementary material for: Epstein–Barr virus-induced gene 3 commits human mesenchymal stem cells to differentiate into chondrocytes via endoplasmic reticulum stress sensor
Source: PLoS One. 2022 Dec 22;17(12):e0279584. doi: 10.1371/journal.pone.0279584 (PMC9778607; doi:10.1371/journal.pone.0279584)
Supplement: S1 Table — (DOCX) [file pone.0279584.s013.docx]

**Supplemental Tables**

**S1 Table. Key resources table**

|  | **Source** | **Identifier** |
| --- | --- | --- |
| **Antibodies** |  |  |
| Anti-IL-35 p35 mouse monoclonal antibody | R&D Systems | Cat # MAB1570 |
| Anti-β-actin mouse monoclonal antibody | Sigma-Aldrich | Cat # A1978 |
| Anti-Calnexin mouse monoclonal antibody | Santa Cruz Biotechnology | Cat # sc-46669 |
| Anti-CD105 mouse monoclonal antibody | Santa Cruz Biotechnology | Cat # sc-18838 |
| Anti-ATF6 mouse monoclonal antibody | Abcam | Cat # ab122897 |
| Anti-phospho-IRE1 (S724) rabbit monoclonal antibody | BOSTER | Cat # P00683 |
| Anti-IRE1α rabbit monoclonal antibody | Cell Signalling Technology | Cat # 3294 |
| Anti-PERK rabbit monoclonal antibody | Cell Signalling Technology | Cat # 3192 |
| Anti-Phospho-NF-κB p65 (Ser536) (93H1) rabbit monoclonal antibody | Cell Signalling Technology | Cat # 3033 |
| Anti-NF-κB p65 mouse monoclonal antibody | Santa Cruz Biotechnology | Cat # sc-8008 |
| Anti-EBI3 rabbit polyclonal antibody | Proteintech | Cat # 12371-1-AP |
| Anti-type II collagen rabbit polyclonal antibody | LifeSpan BioSciences | Cat # LS-C18868 |
| Anti-phospho-PERK (Thr 981) rabbit polyclonal antibody | MyBioSource | Cat # MBS9416902 |
| Anti-IL-27 p27 goat polyclonal antibody | R&D Systems | Cat # AF2526 |
| Anti-p75 NGF Receptor / CD271 goat polyclonal antibody | GeneTex | Cat # GTX89556 |
| Anti-p62 (SQSTM1) rabbit polyclonal antibody | MBL | Cat # PM045 |
| Alexa Fluor 488- conjugated anti-mouse secondary antibody | Thermo Fisher Scientific | Cat # A28175 |
| Alexa Fluor 568-conjugated anti-rabbit secondary antibody | Thermo Fisher Scientific | Cat # A11036 |
| Alexa Fluor 350-conjugated anti-goat secondary antibody | Thermo Fisher Scientific | Cat # A21081 |
| Rhodamine-labeled anti-mouse IgG secondary antibody | Thermo Fisher Scientific | Cat # F31663 |
| FITC-labeled anti-rabbit IgG secondary antibody | Sigma-Aldrich | Cat # F9887 |
| ECL Anti-mouse IgG HRP-Linked Whole antibody | GE healthcare | Cat # NA931V |
| ECL Anti-Rabbit IgG HRP-Linked Whole antibody | GE healthcare | Cat # NA934V |
| **Chemicals, Recombinant Proteins, and medium** |  |  |
| 4-Phenylbutyric acid (4-PBA) | Selleck | Cat # S4125 |
| Tauroursodeoxycholic Acid (TUDCA) | Selleck | Cat # S3654 |
| Tunicamycin (Tm) | R&D Systems | Cat # 65-0865-14 |
| Dithiothreitol (DTT) | Nacalai tesque | Cat # 14130-41 |
| 4′,6-diamidino-2-phenylindole (DAPI) | Santa Cruz Biotechnology | Cat # sc-3598 |
| IL-1β, Human, Recombinant | ReliaTech GmbH | Cat # 400-002 |
| IL-6, Human, Recombinant | R&D Systems | Cat # 206-IL-010 |
| Soluble IL-6 Receptor (sIL-6R), Human, Recombinant | R&D Systems | Cat # 227-SR-025 |
| TNF-α, Human, Recombinant | PeproTech | Cat # 300-01A |
| IL-17A, Human, Recombinant | PeproTech | Cat # AF-200-17 |
| Mesenchymal Stem Cell Growth Medium | Takara | Cat # C-28009 |
| Mesenchymal Stem Cell Chondrogenic Differentiation Medium | Takara | Cat # C-28012 |
| 0.25w/v% Trypsin-1mmol/L EDTA･4Na Solution with Phenol Red | Wako | Cat # 209-16941 |
| OPTI-MEM I | Thermo Fisher Scientific | Cat # 31985062 |
| **Critical Commercial Assays** |  |  |
| Coomassie Brilliant Blue (CBB) | Wako | Cat # 299-50101 |
| Protease inhibitor cocktail | ITSI Biosciences | Cat # A-0014-20 |
| Pierce™ BCA Protein Assay Kit | Thermo Fisher Scientific | Cat # 23225 |
| BenchMark™ Pre-stained Protein Ladder | Invitrogen | Cat # 10748-010 |
| ECL Prime Western Blotting Detection Reagent | GE Healthcare | Cat # RPN 2236 |
| Safranin-O Staining Solution | Sigma-Aldrich | Cat # TMS-009-C |
| Proteinase K | Dako | Cat # S3020 |
| Can get Signal immunostain solution A | Toyobo | Cat # NKB-501 |
| Histofine® Simple StainTM Mouse MAX PO (R) | Nichirei Biosciences | Cat # 424141 |
| ProLong™ Diamond Antifade Mountant | Thermo Fisher Scientific | Cat # P36970 |
| RNeasy Mini kit | Qiagen | Cat # 74106 |
| High-Capacity cDNA Reverse Transcription Kit | Thermo Fisher Scientific | Cat # 4368814 |
| TaqMan Fast Universal PCR Master Mix | Thermo Fisher Scientific | Cat # 4366073 |
| Lipofectamine RNAiMAX Transfection Reagent | Thermo Fisher Scientific | Cat # 13778075 |
| Silencer™ Select Negative Control | Thermo Fisher Scientific | Cat # 4390843 |
| Lipofectamine™ 3000 Transfection Reagent | Thermo Fisher Scientific | Cat # L3000001 |
| Anti-V5-tag pAb-Agarose | MBL | Cat # PM003-8 |
