# Supplementary material for: Epstein–Barr virus-induced gene 3 commits human mesenchymal stem cells to differentiate into chondrocytes via endoplasmic reticulum stress sensor
Source: PLoS One. 2022 Dec 22;17(12):e0279584. doi: 10.1371/journal.pone.0279584 (PMC9778607; doi:10.1371/journal.pone.0279584)
Supplement: S2 Table — (DOCX) [file pone.0279584.s014.docx]

**S2 Table.** Quantitative reverse transcription (RT-q) PCR. TaqMan Gene Expression Assay (Applied Biosystems) primer/probe pairs were used as follows.

| **Gene** | **Assay ID** |
| --- | --- |
| *GAPDH* | Hs99999905_m1 |
| *EBI3* | Hs01057148_m1 |
| *SOX9* | Hs00165814_m1 |
| *COL2A1* | Hs00264051_m1 |
| *ACAN* | Hs00153936_m1 |
| *COL10A1* | Hs00166657_m1 |
| *IL-27 p28* | Hs00377366_m1 |
| *IL-35 p35* | Hs01073447_m1 |
| *RUNX2* | Hs01047973_m1 |
| *MMP1* | Hs00899658_m1 |
| *MMP3* | Hs00968305_m1 |
| *MMP13* | Hs00233992_m1 |
