# Supplementary material for: Epstein–Barr virus-induced gene 3 commits human mesenchymal stem cells to differentiate into chondrocytes via endoplasmic reticulum stress sensor
Source: PLoS One. 2022 Dec 22;17(12):e0279584. doi: 10.1371/journal.pone.0279584 (PMC9778607; doi:10.1371/journal.pone.0279584)
Supplement: S1 File — (ZIP) [file pone.0279584.s015.zip › S1 files/Figure 1 data.pdf]

EBI3

Day	0	2	4	7	14	21
	1	6.559483	9.766772	15.69741	1.562556	0.500645
	1	3.492871	6.11503	22.9727	2.290483	0.04817
	1	3.440171	9.568123	11.61798	2.798307	0.44817

p35

Day	0	2	4	7	14	21
	1	0.652581	0.906994	2.147936	1.540218	0.04815
	1.593452	1.022238	0.865428	1.32311	0.752463	0.102215
	1	0.741118	0.702224	1.653341	1.14778	0.078278

p28

Day	0	2	4	7	14	21
	0	0.246541	1.198558	0.839957	0.234899	0.834513
	0	0.115467	1.686745	0.692002	0.283875	1
	0	0.139541	1.251175	1.139115	0.148832	1
