# Supplementary material for: Epstein–Barr virus-induced gene 3 commits human mesenchymal stem cells to differentiate into chondrocytes via endoplasmic reticulum stress sensor
Source: PLoS One. 2022 Dec 22;17(12):e0279584. doi: 10.1371/journal.pone.0279584 (PMC9778607; doi:10.1371/journal.pone.0279584)
Supplement: S2 File — (ZIP) [file pone.0279584.s016.zip › S2 files/FIgure 2 data.pdf]

No transfection	Ctrl	si#1	si#2
1	1.833761	0.109975	0.055863
1	1.599163	0.248225	0.044918
1	1.479884	0.331234	0.062267

Wet weight

Ctrl	si#1	si#2
0.9	0.5	0.1
0.8	0.6	0.1
1.1	0.7	0.2
0.9	0.5	0.2

S-O IOD

Ctrl	si#1	si#2
2571.428	878.801	80.555
2099.521	581.225	203.823
1834.252	1053.884	399.416
1508.745	773.021	101.565

COL2 IOD

Ctrl	si#1	si#2
2523.318	1221.072	200.014
1833.544	950.347	456.112
1509.882	1207.226	580.181
2088.401	1887.558	710.441

PCR

SOX9			ACAN			COL2A1			COL10A1		
Ctrl	si#1	si#2	Ctrl	si#1	si#2	Ctrl	si#1	si#2	Ctrl	si#1	si#2
1	0.50639	0.491905	1	0.56505	0.25577	1	0.033155	3.56E-06	1	0.26414	0.115358
0.835822421	0.966852	0.559484	1.291004	0.17389	0.080659	1.285451	0.392981	0.000115	0.893212	0.510548	0.143962
1.2288321	0.899021	0.603346	0.849983	0.482904	0.068349	0.856603	0.032631	0.001502	1.159174	0.46272	0.789619

RUNX2			MMP1			MMP3			MMP13		
Ctrl	si#1	si#2	Ctrl	si#1	si#2	Ctrl	si#1	si#2	Ctrl	si#1	si#2
1	0.855207	1.35583	1	8.352255	74.96105	1	0.646504	0.17739	1	20.54421	31.2144
0.8466265	1.954385	2.133071	0.881268	11.39641	38.52576	1.256642	0.516522	0.24469	1.521415	23.89073	29.46324
1.1239451	1.388497	1.788165	1.032558	19.74829	55.3531	0.815564	0.664102	0.301164	1.344229	37.16521	53.5694

EBI3

Ctrl	si#1	si#2
0.8154895	8.88E-06	3.98E-06
1.1991634	0.015318	0.000143
