# Supplementary material for: Epstein–Barr virus-induced gene 3 commits human mesenchymal stem cells to differentiate into chondrocytes via endoplasmic reticulum stress sensor
Source: PLoS One. 2022 Dec 22;17(12):e0279584. doi: 10.1371/journal.pone.0279584 (PMC9778607; doi:10.1371/journal.pone.0279584)
Supplement: S3 File — (ZIP) [file pone.0279584.s017.zip › S3 files/Figure 3 data.pdf]

Wetweight

Mock	pEF6-*EBI3*-V5
0.5	0.3
0.7	0.2
0.8	0.2

EBI3

1	1157996.125
1	1753216.122
1	1518014.497

S-O IOD

Mock	pEF6-*EBI3*-V5
6173.341	49.774
3212.511	101.899
2455.454	80.335
2644.014	90.112

COL2 IOD

Mock	EF6-*EBI3*-V5
5497.002	341.76
3912.885	1136.982
2881.067	51.455
3042.114	120.884

PCR

SOX9

Mock	pEF6-*EBI3*-V5
1	3.824866056
1.174423	4.18920064
0.839974	1.528295159

ACAN

Mock	EF6-*EBI3*-V5
1	0.262274
0.944012	0.249705
1.124511	0.447384

COL2A1

Mock	EF6-*EBI3*-V5
1	0
0.851129	0.008773
1.196542	0.010152

COL10A1

Mock	pEF6-*EBI3*-V5
1	0.690323949
1.231269	0.094991706
0.855141	0.255572706

RUNX2

Mock	pEF6-*EBI3*-V5
1	2.390738
0.864148	1.469503
1.133413	1.999601

MMP1

Mock	EF6-*EBI3*-V5
1	14.57048
1.259521	21.50779
1.727783	30.64772

MMP3

Mock	EF6-*EBI3*-V5
1.216545	1.416408
0.996419	1.486909
0.871215	2.220262

MMP13

Mock	pEF6-*EBI3*-V5
1	5.303646
0.886694	4.135318
0.742457	1.618246
1	6.233429

EBI3

Mock	pEF6-*EBI3*-V5
1	4761156
1.318852	3142450.5
