# Supplementary material for: Epstein–Barr virus-induced gene 3 commits human mesenchymal stem cells to differentiate into chondrocytes via endoplasmic reticulum stress sensor
Source: PLoS One. 2022 Dec 22;17(12):e0279584. doi: 10.1371/journal.pone.0279584 (PMC9778607; doi:10.1371/journal.pone.0279584)
Supplement: S4 File — (ZIP) [file pone.0279584.s018.zip › S4 files/Figure 4 data.pdf]

PCR

EBI3

	NS		IL-6/sIL-6R		IL-1β		TNF-α		IL-17A	
6 hrs	1.407287	0.59966	1.146218	0.620382	53.56959	23.49848	2.878734	2.164933	1.77091	1.154526
24 hrs	0.935939	0.835705	0.816036	0.53461	55.39263	20.42414	2.389927	1.502714	1.035313	0.723823
72 hrs	1	1	0.596682	0.509702	25.24571	11.58978	1.176232	1.278948	1.469468	0.863527

IL-35p35

	NS		IL6/sIL6R		IL1b		TNFa		IL-17A	
6 hrs	10.04285	5.074751	9.43779	6.053569	10.93243	4.372546	11.44221	5.24381	11.50562	6.119878
24 hrs	3.283748	0.976502	2.874877	1.048285	1.689575	0.961207	2.327687	1.548285	2.89313	1.036538
72 hrs	1	1	1.176154	0.948529	1.259612	0.772852	1.892653	1.061795	0.856827	0.397242

IL-27p28

	NS		IL6/sIL6R		IL1b		TNFa		IL-17A	
6 hrs	0	0	0	1.430154	2.075783	2.917723	1.905619	0	2.220271	1.31718
24 hrs	0.909436	0.645833	0	0.795094	0.783962	1.611697	2.04101	5.000499	0	0.260173
72 hrs	1	1	0	3.135674	0	2.798181	1.147456	3.717387	1.560334	1.95097

wet weight

NS	IL-6/sIL-6R	IL-1β
0.8	1.2	0.2
1	1.4	0.3
0.8	1.1	0.2

S-O IOD

NS	IL-6/sIL-6R	IL-1β
820.509	1510.889	78.177
752.481	1210.605	92.522
1901.433	2310.412	100.245
817.402	901.113	75.888

COL2 IOD

NS	IL-6/sIL-6R	IL-1β
2155.492	3134.821	24.926
1553.017	2616.992	50.119
2077.801	2299.405	102.556

PCR

EBI3

NS	IL-6/sIL-6R	IL-1β
1	0.849371	590.4712
1.563744	5.466433	337.159
1.297883	1.562556	437.6445

SOX9

NS	IL-6/sIL-6R	IL-1β
1	0.783482	0.118064
0.899554	1.005885	0.447608
1.259564	0.966852	0.442852
1	1.123945	0.123165

ACAN

NS	IL-6/sIL-6R	IL-1β
1	2.608047	0.000608
1.662547	4.981519	0.000486
0.956488	2.798307	0.001715
1	3.492871	0.0128

COL2A1

NS	IL-6/sIL-6R	IL-1β
1	4.981519	0.000875
1.196567	7.880718	0
2.564112	2.390738	0
1	2.303646	0

COL10A1

NS	IL-6/sIL-6R	IL-1β
1	2.849953	0.023109
1.339597	5.988576	0.022652
1.2456	2.220262	0.011889
1	1.124511	0.012738

RUNX2

NS	IL-6/sIL-6R	IL-1β
1	0.602993	4.09807
1.124521	1.115963	3.399102
1.852254	0.996419	4.559486
1	1.231269	4.253062

MMP1

NS	IL-6/sIL-6R	IL-1β
1	0.240184	715.0491
2.045126	1.574911	835.4918
1.453783	1.257421	365.113
1	0.864177	188.4547

MMP3

NS	IL-6/sIL-6R	IL-1β
1	0.244526	2.527287
0.865459	1.24009	3.05921
1	0.855054	4.878358

MMP13

NS	IL-6/sIL-6R	IL-1β
1	0.609781	18.45833
0.962212	2.066596	11.28991
1.561174	2.098147	7.881373
1	1.520846	15.71287
