# Supplementary material for: Epstein–Barr virus-induced gene 3 commits human mesenchymal stem cells to differentiate into chondrocytes via endoplasmic reticulum stress sensor
Source: PLoS One. 2022 Dec 22;17(12):e0279584. doi: 10.1371/journal.pone.0279584 (PMC9778607; doi:10.1371/journal.pone.0279584)
Supplement: S5 File — (ZIP) [file pone.0279584.s019.zip › S5_C files/Figure 5 data.pdf]

WB

NS			IL-1b			IL-6/sIL6R		
Sic	#1	#2	Sic	#1	#2	Sic	#1	#2
1	0.528966	0.885192	4.02436	3.590298	2.566072	0.818322	1.5745	0.82027
0.722671	1.42048	0.854479	4.142804	4.392893	2.098147	2.018526	1.520846	1.12265
1.574175	1.031189	1.007002	3.088663	2.459952	2.420216	1.51767	2.480789	2.304279

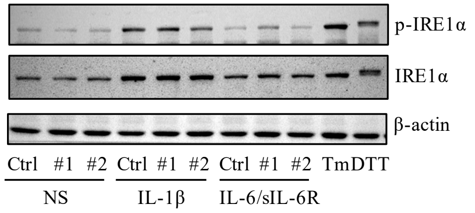

	p-IRE1		total	p-IRE1/IRE1			actin		pIRE1/actin
1	3163.426	1	6944.497	0.45553	1	1	13856.91	0.228292278	1
2	1914.912	2	6496.326	0.294768	0.647089	2	15857.33	0.120758822	0.528966
3	3121.669	3	8014.497	0.389503	0.855054	3	15447.5	0.202082512	0.885192
4	12517.62	4	15331.5	0.816464	1.79234	4	13624.91	0.918730264	4.02436
5	12853.62	5	16121.03	0.79732	1.750313	5	15682.08	0.819637225	3.590298
6	9599.205	6	16207.03	0.592286	1.300214	6	16386.08	0.585814499	2.566072
7	2825.376	7	10181.5	0.277501	0.609183	7	15123.79	0.186816664	0.818322
8	6066.912	8	12082.91	0.502107	1.102248	8	16878.5	0.359446223	1.5745
9	2943.255	9	9957.255	0.295589	0.64889	9	15717.38	0.18726122	0.82027

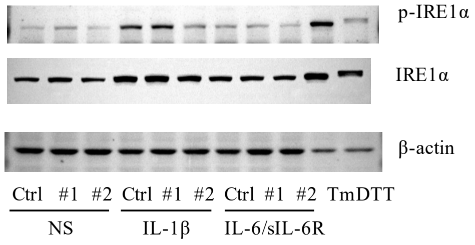

	p-IRE1		total				actin		pIRE1/actin
1	2468.205	1	8709.962	0.283377	0.622083	1	14960.62	0.164980139	0.722671
2	5315.74	2	11325.91	0.469343	1.030324	2	16392.21	0.324284622	1.42048
3	3314.205	3	9165.376	0.361601	0.793802	3	16989.74	0.195070966	0.854479
4	13611.33	4	15923.38	0.854802	1.876499	4	14391.79	0.945770193	4.142804
5	15452.62	5	16855.08	0.916793	2.012585	5	15408.5	1.002863485	4.392893
6	7770.205	6	14614.38	0.531682	1.167173	6	16222.03	0.478990827	2.098147
7	6489.083	7	12375.79	0.524337	1.151048	7	14081.79	0.460813789	2.018526
8	5953.912	8	11715.91	0.50819	1.115602	8	17148.5	0.347197308	1.520846
9	3881.719	9	10004.06	0.388014	0.851787	9	15145.67	0.256292343	1.12265

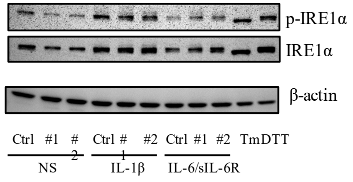

	p-IRE1		total				actin		p-IRE1/actin
1	6270.154	1	7455.012	0.359372	1	1	13912.38	#DIV/0!	1.574175
2	4036.569	2	5313.598	0.235413	0.522339	2	17146.79	#DIV/0!	1.031189
3	4174.012	3	6224.134	0.229891	0.510088	3	18156.5	#DIV/0!	1.007002
4	12066.57	4	10946.31	0.705118	1.564533	4	17112.84	#DIV/0!	3.088663
5	8730.891	5	10759.76	0.561588	1.246066	5	15546.79	#DIV/0!	2.459952
6	9427.184	6	12267.01	0.621004	1.3779	6	15180.55	#DIV/0!	2.720216
7	4805.619	7	6622.305	0.346472	0.768762	7	13870.13	#DIV/0!	1.51767
8	7431.255	8	7851.305	0.566345	1.256621	8	13121.43	#DIV/0!	2.480789
9	7214.962	9	8736.184	0.526049	1.167211	9	13715.38	#DIV/0!	2.304279
