# Supplementary material for: Epstein–Barr virus-induced gene 3 commits human mesenchymal stem cells to differentiate into chondrocytes via endoplasmic reticulum stress sensor
Source: PLoS One. 2022 Dec 22;17(12):e0279584. doi: 10.1371/journal.pone.0279584 (PMC9778607; doi:10.1371/journal.pone.0279584)
Supplement: S6 File — (ZIP) [file pone.0279584.s020.zip › S6 files/Supplemental figure data.pdf]

S1 A EBI3

	NS		IL-6/sIL-6R		IL-1β		TNF-α		IL-17A	
12 hrs	2.539937973	1.745687	2.613830566	1.491436	43.04980087	33.01474	21.56993866	20.67517	1.835692	3.444973
24 hrs	1.32357502	1.63209	2.251244783	0.771392	25.58511353	25.4427	17.04555702	11.9921	2.338078	2.374769
36 hrs	1	1	1.680692554	1.269952	16.75518799	21.7385	10.64937496	17.58131	0.836029	0.828594

IL-35p35

	NS		IL-6/sIL-6R		IL-1β		TNF-α		IL-17A	
12 hrs	3.701889515	2.202819	3.261806726	2.49779	4.352094173	2.740156	4.175610065	2.474496	3.590367	4.039144
24 hrs	1.911098957	2.109563	1.291175246	2.053322	2.4449718	3.597847	2.668337584	2.412286	1.414737	2.584075
36 hrs	1	1	1.233961225	0.771463	1.937757015	1.340833	1.889820337	1.924379	0.855934	0.901968

S1B

EBI3

0	0.1	0.3	1	3	10	100	IL-1b ng/ml
1			16.2598	20.12045	17.65451	15.21854	
1	1.758336	12.29684	14.39683	29.50996	11.16277	7.133678	
1	4.263423	14.56265	19.08011	33.55094	14.62899		

EBI3

0	1	10	100	TNFa ng/ml
1	4.958754	14.70964	36.35676	
1	1.775067	10.5852	21.439118	

1	0.88349855	1.508074	0.404019962	1.188743	0.95451114	1.712241	1.52465621	
0.96844	0.633635526	1.276906	0.640543079	1	0.9587441	1.420195	1.18851011	
1	0.552022633	1.076561	0.611943879	1.27964	1.154574916	1.50627	1.009179195	
−	−	+	+	−	−	+	+	IL-1β
+	−	+	−	−	−	−	−	PBS
−	+	−	+	−	−	−	−	4-PBA
−	−	−	−	+	−	+	−	DMSO
−	−	−	−	−	+	−	+	TUDCA

S4 C

NS		IL-1b	
Ctrl	4-PBA	Ctrl	4-PBA
0.620844	119.3958511	12.04552	455.7340088
4.328825	178.1486816	19.72408	544.0009766

NS		IL-1b	
Ctrl	TUDCA	Ctrl	TUDCA
1	0.720249832	13.10731	10.19477654
1.059855	0.525594294	10.94149	8.556694984
1.329965	1.082866073	16.83848	14.66830158

S8B WB

DMSO	DTT	Tm
1	0.993023	1.416224
1.035404	1.384927	1.811987
1.006178	2.266919	1.158215
1	1.269589553	1.569107

S9B

pNF-kB/NF-kB

NS	IL1b	NS+PBA	IL1b+PBA
1	2.146088723	1.889095	2.579120335
1.354695	2.516337216	1.905313	2.879676384

p62/actin

NS	IL1b	NS+PBA	IL1b+PBA
1	2.373874	0.728723599	0.920151
1.670677511	1.983347	0.948007805	1.241037

S11A

Day 0			Day 2			Day 4			Day7		
Ctrl	si#1	si#2	Ctrl	si#1	si#2	Ctrl	si#1	si#2	Ctrl	si#1	si#2
1	0.488758	0.135384	1	0.517117	0.169773	1	0.121529	0.027213	1	1.021663	0.545128
1.343816	0.518367	0.129226	0.772328	0.23722	0.283987	5.809892	0.375808	0.615877	3.400918	0.456675	0.211737
			2.135387	0.819716	0.053748	5.517169	0.680912	0.042081			

Day 14

Ctrl	si#1	si#2
1	0.507736	0.140179
1.609493	0.299984	0.277733

S11B

Day 0		Day 2		Day 4		Day 7		Day14	
Mock	pEF6-*EBI3*-V5	Mock	pEF6-*EBI3*-V5	Mock	pEF6-*EBI3*-V5	Mock	pEF6-*EBI3*-V5	Mock	EF6-*EBI3*-V5
1	540838	1	884564.125	1	643340.1875	1	32912.64453	1	324044
0.634621	237596.8594	1.949797	245955.0781	4.579029	105713.6484	0.046895	3520.81665	175.2469	244742.3
		1.295832	599430.6875	3.494084	265490.9063	0.477022	4398.879883		
